# A phosphoproteome atlas of human cell lines reveals the landscape of kinase activity

**DOI:** 10.1038/s41594-026-01877-6

**Published:** 2026-09-30

**Authors:** Claire Koenig, Hayoung Cho, Kristina B. Emdal, Ilaria Piga, Pierre Sabatier, Samuel Lozano-Juárez, Ana Martinez-Val, Jesper V. Olsen

**Affiliations:** 1 Novo Nordisk Foundation Center for Protein Research and Copenhagen Center for Glycocalyx Research, Department of Cellular and Molecular Medicine, Faculty of Health and Medical Sciences, University of Copenhagen, Copenhagen, Denmark; 2 Department of Paediatrics and Adolescent Medicine, Copenhagen University Hospital Rigshospitalet, Copenhagen, Denmark; 3 Cardio-Thoracic Translational Medicine Lab, Department of Surgical Sciences, Uppsala University, Uppsala, Sweden; 4 Cardiovascular Proteomics Laboratory, Centro Nacional de Investigaciones Cardiovasculares Carlos III, Madrid, Spain; 5 Centro de Investigación Biomédica en Red de Enfermedades Cardiovasculares, Madrid, Spain

**Keywords:** Mass spectrometry, Proteomics, Phosphorylation, Cell signalling, Kinases

## Abstract

Protein phosphorylation orchestrates cellular signaling and controls most biological processes, with its dysregulation driving diseases, notably cancer. Comprehensive, high-throughput phosphoproteomics remains limited by detection sensitivity, data completeness and computational bottlenecks, especially in low-input settings. Here we present a comprehensive empirical human phosphoproteome resource, regrouping over 200,000 class I phosphosites across 33 diverse human cell lines. We demonstrate that this spectral library dramatically improves single-shot phosphoproteomics with 30-fold faster data processing compared with library-free approaches and enhances confidence in phosphosite localization even from minimal sample input. Integrating proteome and phosphoproteome data, we develop a combined kinase activity score (Cscore), revealing cell line- and cancer-specific signaling vulnerabilities, many correlating with drug sensitivity. This resource accelerates deep and reproducible phosphoproteomics, enables the systematic mapping of cellular signaling networks and may empower precision oncology by highlighting actionable kinase targets in diverse cell states.

## Abstract (web summary)

Koenig et al. present a phosphoproteome atlas of 33 human cell lines covering >200,000 phosphosites, advancing cellular kinase activity scoring and drug sensitivity prediction, providing a resource for cell signaling insights and precision oncology.

## Main

Cellular signaling is dynamically regulated by thousands of coordinated site-specific protein phosphorylation events that determine how cells grow, respond to stimuli or promote disease. Protein phosphorylation is catalyzed by protein kinases and erased by protein phosphatases, and the cellular interplay between these enzymes is usually tightly controlled, making protein phosphorylation one of the most important post-translational modifications (PTMs). Phosphorylation-dependent mechanisms control diverse biological processes, and each cell type, with its unique function and regulatory circuitry, is expected to exhibit a distinct phosphoproteome. Beyond enhancing our understanding of cellular biology, global phosphoproteomics has demonstrated efficiency in understanding cancer biology, where dysregulated kinase signaling rewires essential communication networks and promotes aberrant cellular progression, providing actionable insights into therapeutic vulnerabilities^1–3^. Mass spectrometry (MS)-based phosphoproteomics has become the primary method for simultaneously identifying and quantifying protein phosphorylation events, with up to ~90,000 class 1 phosphosites detected in large-scale studies^4,5^, providing invaluable insights into the regulatory layers governing cellular behavior^6,7^. Extracting meaningful biological information can be facilitated with larger datasets providing greater coverage and completeness. While large-scale initiatives aimed at making large genomics, transcriptomics and proteomics datasets available across tissues or cancers, such as the Human Protein Atlas^8^, the Dependency Map^9,10^ or CPTAC^11^, phosphoproteomics profiling is often absent or limited to cancer material.

Despite rapid and substantial developments, accessing the full depth of the phosphoproteome remains challenging. Many phosphorylation events are highly dynamic and occur at very low stoichiometry, spanning across a wide abundance range, making their detection by liquid chromatography (LC)–tandem MS (MS/MS) challenging. Historically, quantifying 30,000 phosphosites required large input amounts and extensive offline fractionation^12^. Today, technical advances combining fast and sensitive LC–MS/MS have enabled comparable coverage in single-shot analyses with dramatically less material, including reports of ~30,000 phosphosites measured in 30-min gradients^13,14^. Unlike conventional bottom-up proteomics where most proteins are quantified by many peptide precursors, phosphoproteomics mainly relies on single precursor-level quantification and confident site localization, which greatly increases susceptibility to missing values, particularly in low-input settings.

While data-dependent acquisition was historically the method of choice for phosphoproteomics^15,16^, the advent of spectral-library-free data-independent acquisition (DIA) approaches have simplified experimental design and substantially increased coverage^17^. Yet, the rising scale of modern studies with automated sample processing workflows, combined with the high acquisition speed of modern MS instruments, creates a new bottleneck: searching and processing the resulting massive LC–MS/MS datasets^18^, demanding substantial computational resources and long search times.

Here, we leveraged state-of-the-art narrow-window DIA (nDIA) analysis^19^ to investigate the extent and regulation of the MS-detectable human phosphoproteome across a panel of 33 human cell lines representing the major organs, tissues and cancer cells in the body. We generated a deep phosphoproteome atlas to build an empirical spectral library, evaluate its impact on DIA phosphoproteomics, compute phosphorylation stoichiometry and develop a combined kinase activity score (Cscore) for identifying cell line-specific signaling dependencies.

## Results

### Analysis of 33 human cell lines results in exceptionally high coverage of the human proteome and phosphoproteome

#### Building of the human cell line panel and generation of the proteomics and phosphoproteomics profiles

**Fig. 1:**
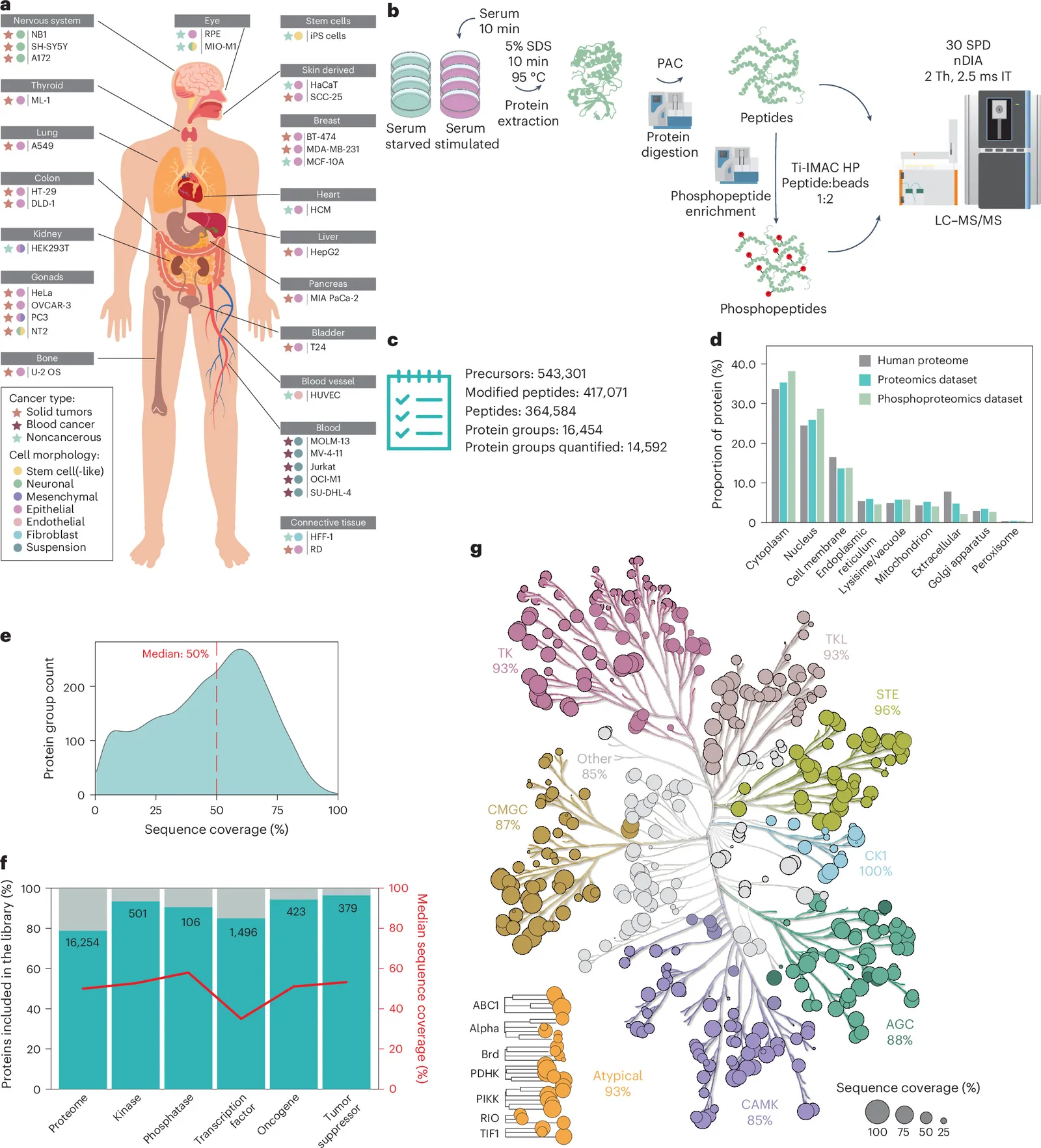
Overview of the cell line panel, experimental workflow and proteome coverage. **a**, Overview of the 33 human cell lines used to build the panel, organized according to their tissue of origin. **b**, Overview of the experimental workflow applied for the generation of the dataset. Cells were cultured, and half of the replicates were serum stimulated before lysis, followed by protein digestion, phosphopeptide enrichment and LC–MS/MS analysis. The generated raw files were searched per cell line and combined for the generation of (phospho)peptide libraries. **c**, Overview of the depth of the resulting peptide spectral library. **d**, Distribution of the cellular localization of the proteins using predicted localization with DeepLoc^75^ in the human proteome and the generated proteomics and phosphoproteomics datasets. **e**, Protein sequence coverage distribution over the 16,454 protein groups. **f**, Bar plot representing the percentage of proteins included in the peptide library over various protein families. The red line represents their respective median protein sequence coverage. **g**, Kinase map generated using KinMap^23^, where each dot on the kinase tree represents a kinase in the peptide library and the size of the dot represents the protein sequence coverage. The percentages on the plot display the fraction of kinases detected in the library for a given kinase family. Source data

To create a comprehensive experimental human phosphopeptide library representing phospho-signaling in living cells, we assembled a panel of human cell lines, spanning major lineages, cell types and signaling pathways (Fig. 1a). The panel included cancerous and noncancerous phenotypes, primary and immortalized cells, suspension and adherent types, including epithelial, mesenchymal, endothelial and fibroblast morphologies, while early developmental signaling was covered by embryonal cells and induced pluripotent stem (iPS) cells. The cell lines were profiled under unstimulated serum-starved and serum-stimulated conditions, to capture a broad range of regulatory phosphorylation events. In addition, increased phosphotyrosine coverage was achieved by broad-spectrum inhibition of phosphotyrosine protein phosphatases via pervanadate (PV) treatment^20,21^ of the colorectal cancer cell line DLD-1.

Both unenriched peptide mixtures representing the cell line proteomes and phosphopeptide-enriched samples were analyzed using an Evosep One LC system coupled to an Orbitrap Astral mass spectrometer operated in nDIA mode (Fig. 1b).

#### Deep proteome library covers up to 80% of the canonical human proteome

**Extended Data Fig. 1:**
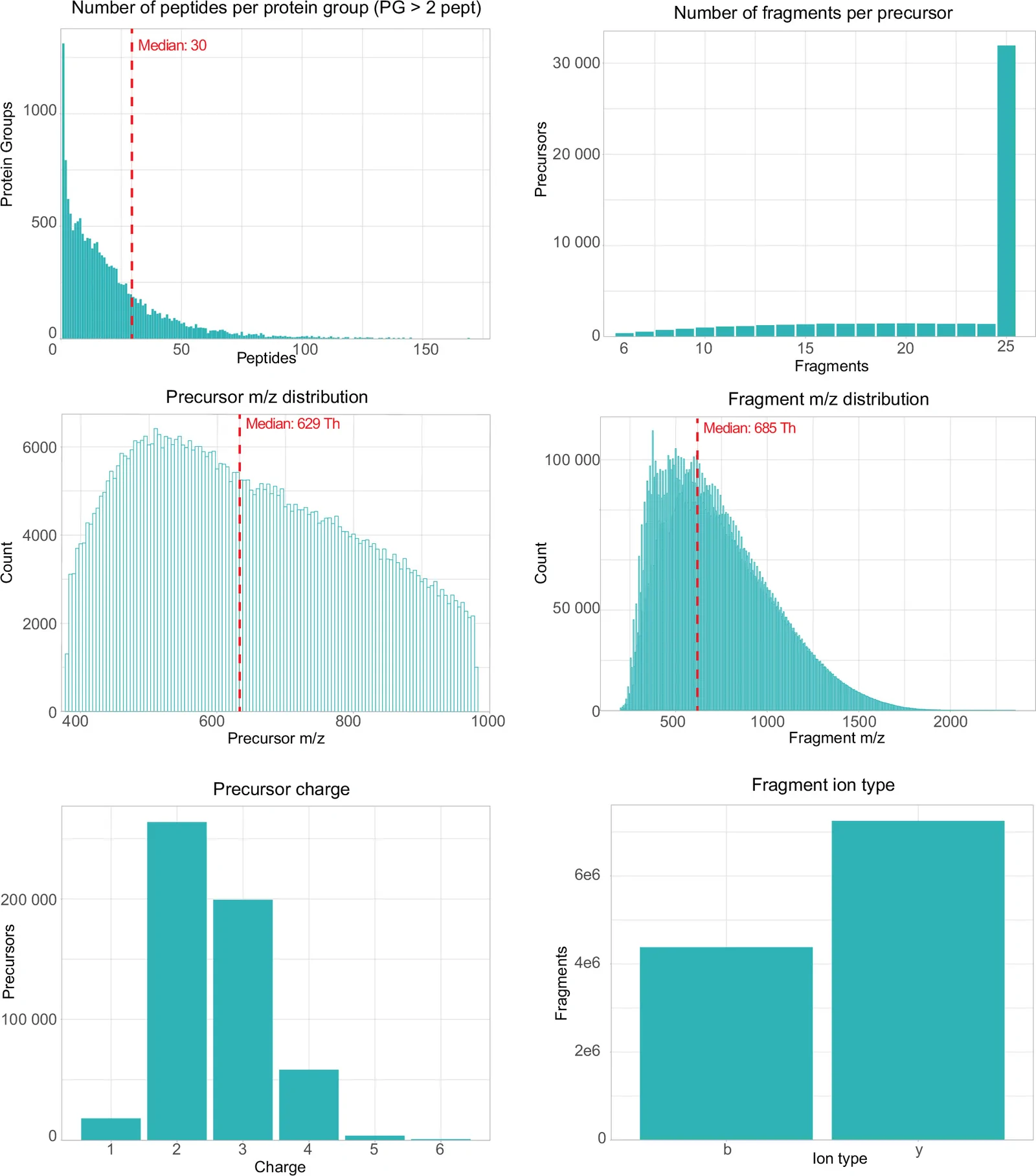
Characteristics of the peptide spectral library. Distributions of the number of peptides per protein groups, number of fragments per precursors, precursor and fragments mass-to-charge ratios, charge states and fragment ion type.

Using a 30 samples-per-day (SPD) LC gradient with the fastest scanning 200-Hz nDIA MS acquisition method, we quantified an average of 9,681 protein groups per file (8,863–10,495; Supplementary Fig. 1a). Collectively, this resulted in the generation of a spectral library comprising 543,301 peptide precursors, corresponding to 364,584 unique peptide sequences and 16,454 protein groups, of which 14,450 remained after filtering for quantified protein groups in at least three replicates of one cell line (Fig. 1c and Extended Data Fig. 1). The quantified proteome was predominantly localized to the cytoplasm and the nucleus, as assessed by DeepLoc predictions^22^ (Fig. 1d), with a distribution across cell compartments following the expected distribution of the human proteome. Overall, the dataset quantified a substantial proportion of the canonical human proteome (~80%), with a median sequence coverage of 50% (Fig. 1e). More than 90% of protein kinases^23^, protein phosphatases, transcription factors (TFs), oncogenes and tumor suppressors^24,25^ were identified with >50% median sequence coverage (Fig. 1f). However, TFs showed slightly lower median sequence coverage (35%), as TFs are generally lower in abundance compared with other proteins. Importantly, nearly the entire human kinome was covered with ~500 protein kinases, without any observable bias in protein detectability or sequence coverage across kinase families (Fig. 1g).

#### Deep phosphopeptide library reveals cell line-specific signaling

**Fig. 2:**
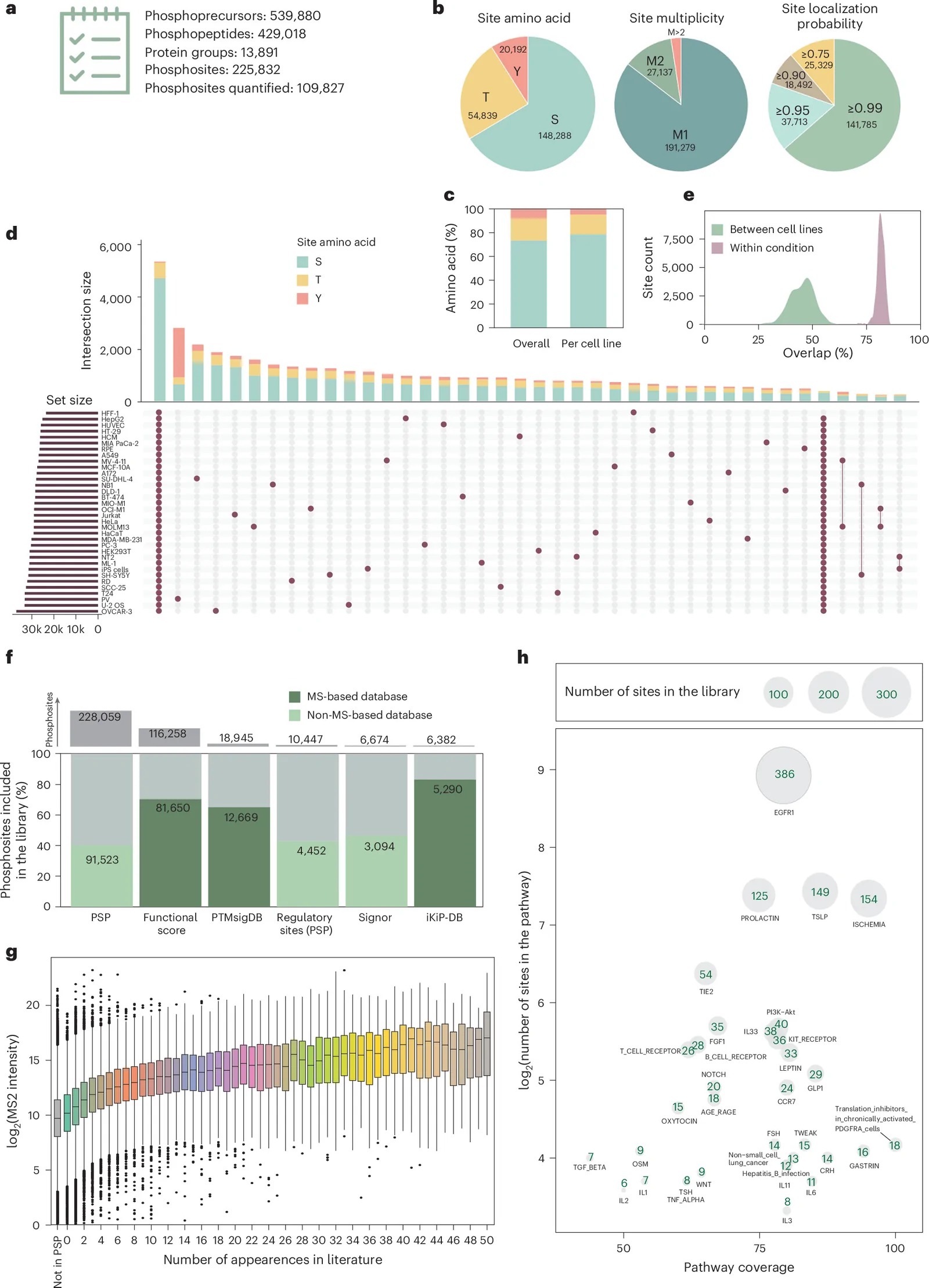
The deep phosphoproteomics library shows high coverage on public databases. **a**, Overview of the depth of the resulting phosphopeptide spectral library. **b**, Site amino acid, site multiplicity and site localization probability distributions over the generated dataset. **c**, Bar plot showing the different proportions of pS, pT and pY between the overall dataset and each cell line individually in the dataset filtered for consistently quantified phosphosites (that is, three-quarters valid value per condition). **d**, Overlap between quantified phosphosites across 33 human cell lines and PV-treated DLD-1 cells, shown as an Upset plot (34 conditions in total) using the dataset filtered for consistently quantified phosphosites. The color of the bars represents the relative proportion of each phosphorylated amino acid. **e**, Distribution of all pairwise overlaps in phosphosite identification across cell line and condition (that is, 408 overlaps representing technical variation) and across cell lines (that is, 561 overlaps representing biological variation). **f**, Bar plot representing the proportion of phosphosites overlapping between our empirical phosphopeptide spectral library and public databases. Darker bars represent the overlap with databases generated using MS. The top gray bars represent the total number of phosphosites in the database. **g**, Relation between the measured MS2 intensity of phosphosites and their number of appearances in MS-based literature according to PSP over the 91,523 phosphosites shared between PSP and our library. Box plots show the median (center line), the first and third quartiles (box limits) and the minimum and maximum values (whiskers). **h**, Coverage over curated signaling pathways from PTMSigDB^28^. The *y* axis represents the total size of the pathway, and the *x* axis displays the phosphosite coverage over the pathway with the library. The number of sites overlapping between the library and the pathway are shown in green. Pathways with at least four detected sites are displayed. Source data

**Extended Data Fig. 2:**
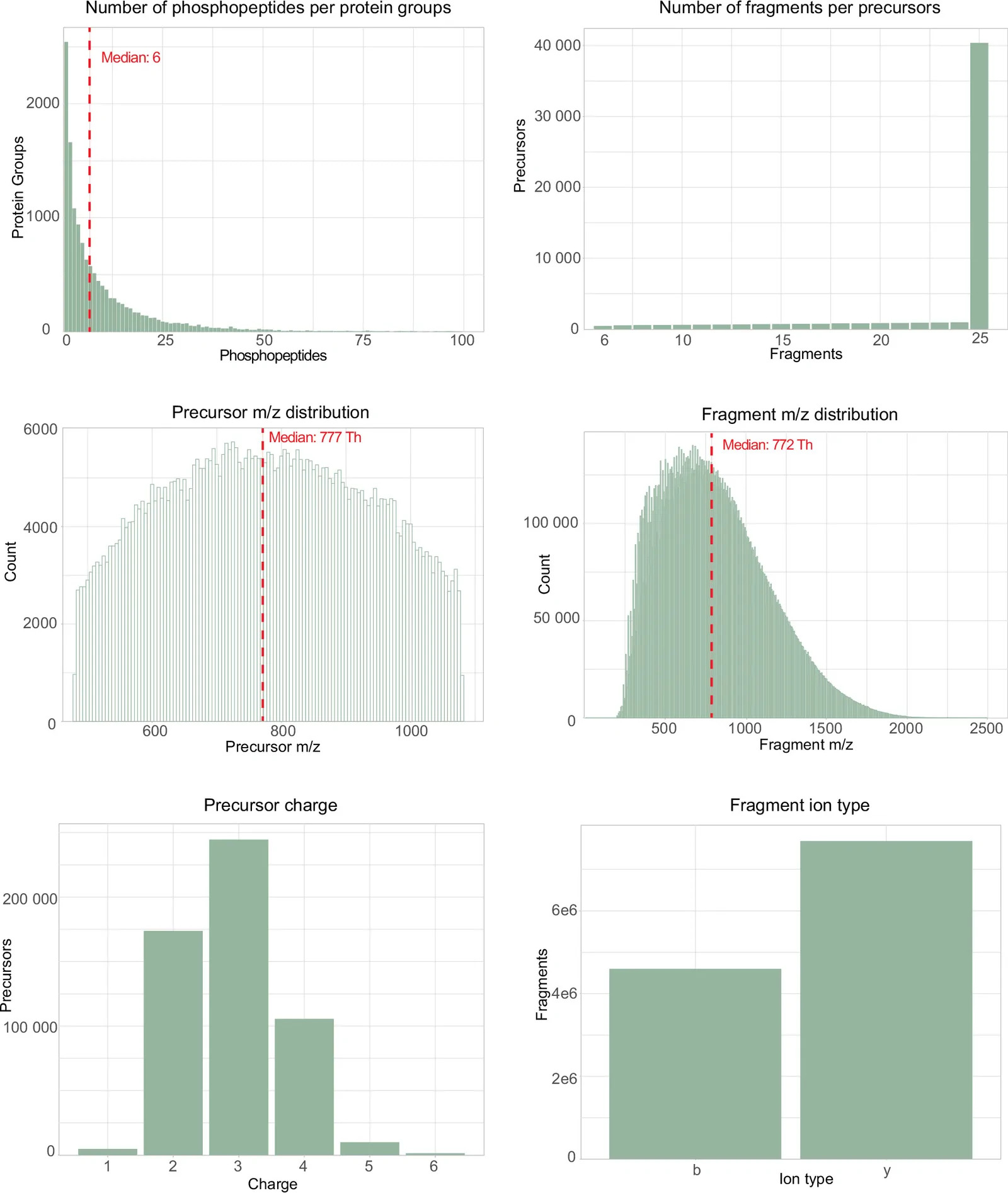
Characteristics of the phosphopeptide spectral library. Distributions of the number of phosphopeptides per protein groups, number of fragments per precursors, precursor and fragments mass-to-charge ratios, charge states and fragment ion type.

From the nDIA analysis of the phosphopeptide-enriched samples, we generated deep phosphoproteomes, with a mean of 30,446 class I phosphosites identified per file (22,702–39,219; Supplementary Fig. 1b). Altogether, the resulting phosphopeptide spectral library contained over 500,000 phosphopeptide precursors spanning 13,891 protein groups, representing 225,832 distinct phosphosites (Fig. 2a and Extended Data Fig. 2). The majority of sites were detected on singly phosphorylated peptides and were localized with high confidence^17^, with almost two-thirds assigned a site localization probability ≥0.99. Overall, the detected phosphosites consisted of 66% phosphoserine (pS), 25% phosphothreonine (pT) and 9% phosphotyrosine (pY) (Fig. 2b). While the proportion of pY is high in the combined library compared with the expected fraction of a few percent^15^, individual cell lines were composed of 81.5% pS, 15% pT and 3.5% pY after filtering for confidently and consistently quantified sites (Fig. 2c). Interestingly, we find that pY and pT proportions in the combined dataset were consistently higher at the expense of pS compared with individual cell line data.

The largest intersection across cell lines corresponded to phosphosites shared across all cell lines, reflecting a core signaling set of phosphosites with low pY content (1%) (Fig. 2d). By contrast, the majority of additional intersections were unique to individual cell lines rather than shared among subsets. The largest unique intersection corresponded to the PV-treated DLD-1, where over two-thirds of those unique sites were pY. The proportion of pY in the further one-cell-line intersections was high, with an average of 11%.

While unique sites per cell line were biased toward the lower intensity range compared with shared sites (Fig. 2d and Supplementary Fig. 2), consistent with an increased detection of low-abundant phosphosites, including pY, the pairwise overlap distribution between sites within technical replicates was higher compared with the pairwise overlap of sites quantified across cell lines (Fig. 2e). This points toward genuine cell line-specific signaling with tyrosine phospho-signaling driven functionality.

#### The dataset shows a high overlap with phosphosites reported in public databases

**Extended Data Fig. 3:**
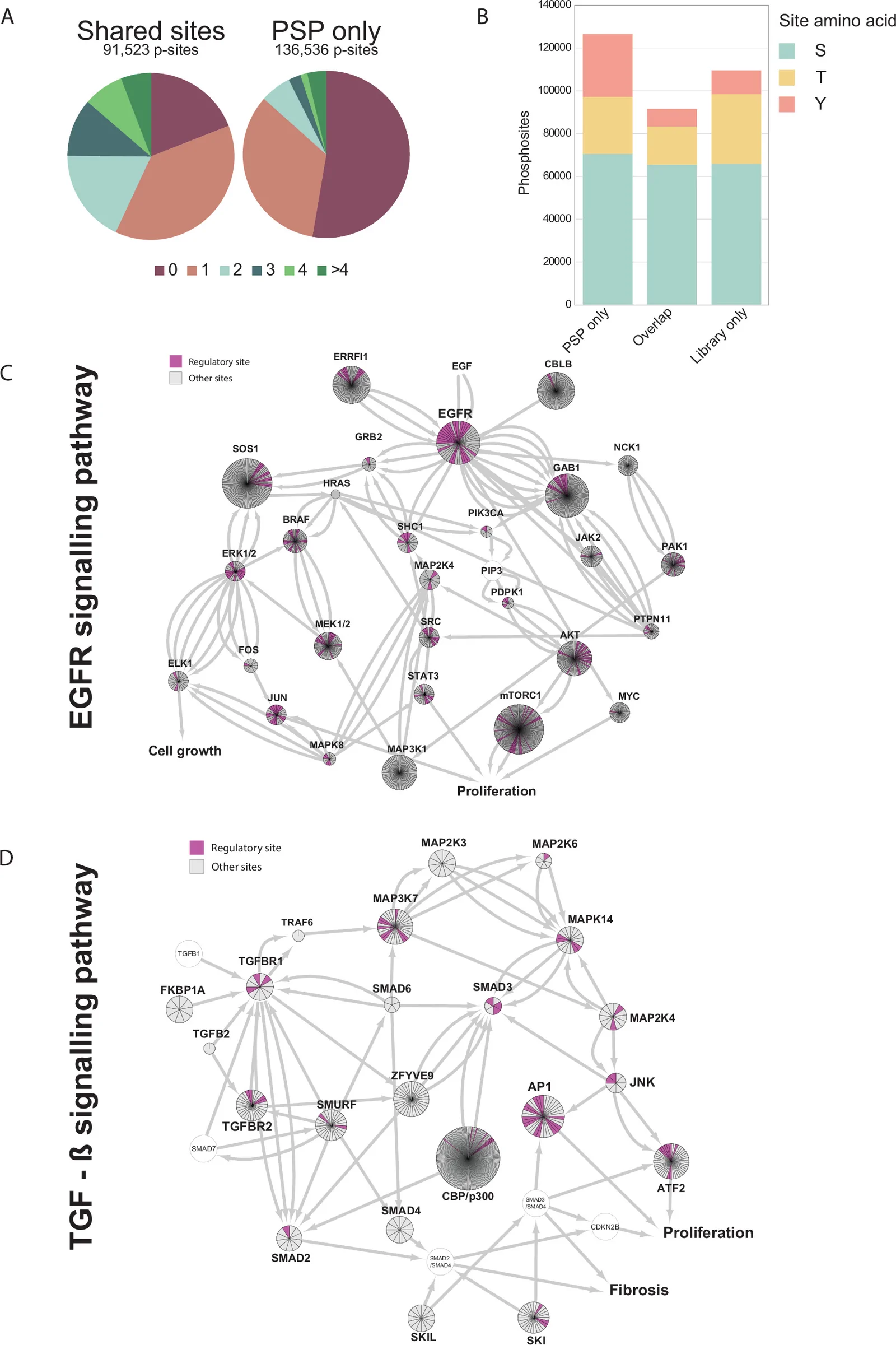
The empirical phosphopeptide library shows high phosphosite coverage of PSP and curated pathways from PTMSig DB. **A**- Venn diagrams showing the proportion of sites with 0, 1, 2, 3, 4 or above 4 references in the literature using MS-based techniques in i) the overlap between the library and PSP, and in ii) the sites unique to PSP. **B**- Phosphosite count overlapping between the library and PSP, unique to PSP and unique to the library, with their respective proportion of pS, pT and pY. **C**- Phosphosite coverage of the EGFR signaling pathway represented as a network. Each slice on the genes represents a phosphosite covered in the library on the given gene. Pink sites are known regulatory sites from PSP. **D**- Phosphosite coverage of the TGF-beta signaling pathway represented as a network. Each slice on the genes represents a phosphosite covered in the library on the given gene. Pink sites are known regulatory sites from PSP. Source data

To benchmark the depth of our dataset and assess coverage of our phosphosite detection, we compared our dataset with (1) PhosphoSite Plus (PSP)^26^, the largest PTM database with >200,000 phosphosites; (2) the manually curated regulatory sites available in PSP; (3) Signor^27^, a database containing experimentally validated causal interactions; (4) PTMsigDB, a site-specific curated MS-based database^28^; (5) the functional score dataset, generated from MS-based publically available data^4^; and (6) the iKiP-DB regrouping curated in vitro kinase reaction-based kinase–substrate relationships^29^. Over 60% of detected sites overlapped with MS-based databases, whereas coverage decreased to ~40% overlap for non-MS-derived resources (Fig. 2f). In total, 81,650 phosphosites overlapped with the functional score dataset and 91,523 with PSP. Among the sites exclusively in PSP, more than half lacked MS-based evidence in literature and showed an enrichment for pY (Extended Data Fig. 3a). Many of these sites represent pY-specific phosphoproteomics datasets based on low-resolution ion trap MS/MS data probably corresponding to very low-intensity events^30,31^, sites intrinsically difficult to detect and reproduce by MS or potential artifacts. Conversely, the fraction without prior MS evidence drops substantially when focusing on the detected sites in our dataset overlapping with PSP (Extended Data Fig. 3b). As site intensity correlates with the number of literature reports in PSP (Fig. 2g), deep and sensitive phosphoproteomics enabled the detection of low-intensity sites lacking previous literature evidence.

Using curated PTMsigDB signatures, we assessed signaling pathway coverage (Fig. 2h). Almost all pathways displayed ≥50% coverage, meaning that more than half of their curated phosphosites were detectable in our spectral library. Coverage generally correlated with pathway size, reaching 386 sites for extensively studied signaling pathways such as EGFR, while only 7 sites were covered for the TGF-β pathway, with a coverage slightly below 50%. As both EGF and TGF-β ligands are present in serum^32^, and their respective receptors are expressed in the proteome, this difference probably reflects incomplete TGF-β annotation rather than ligand or receptor availability. At the gene level, both pathways displayed similar levels of phosphosite coverage, including the presence of known regulatory sites (Extended Data Fig. 3c) supporting broad pathway representation across the diverse cell-line panel.

### Deep experimental spectral library increases the depth of phosphoproteomes

**Fig. 3:**
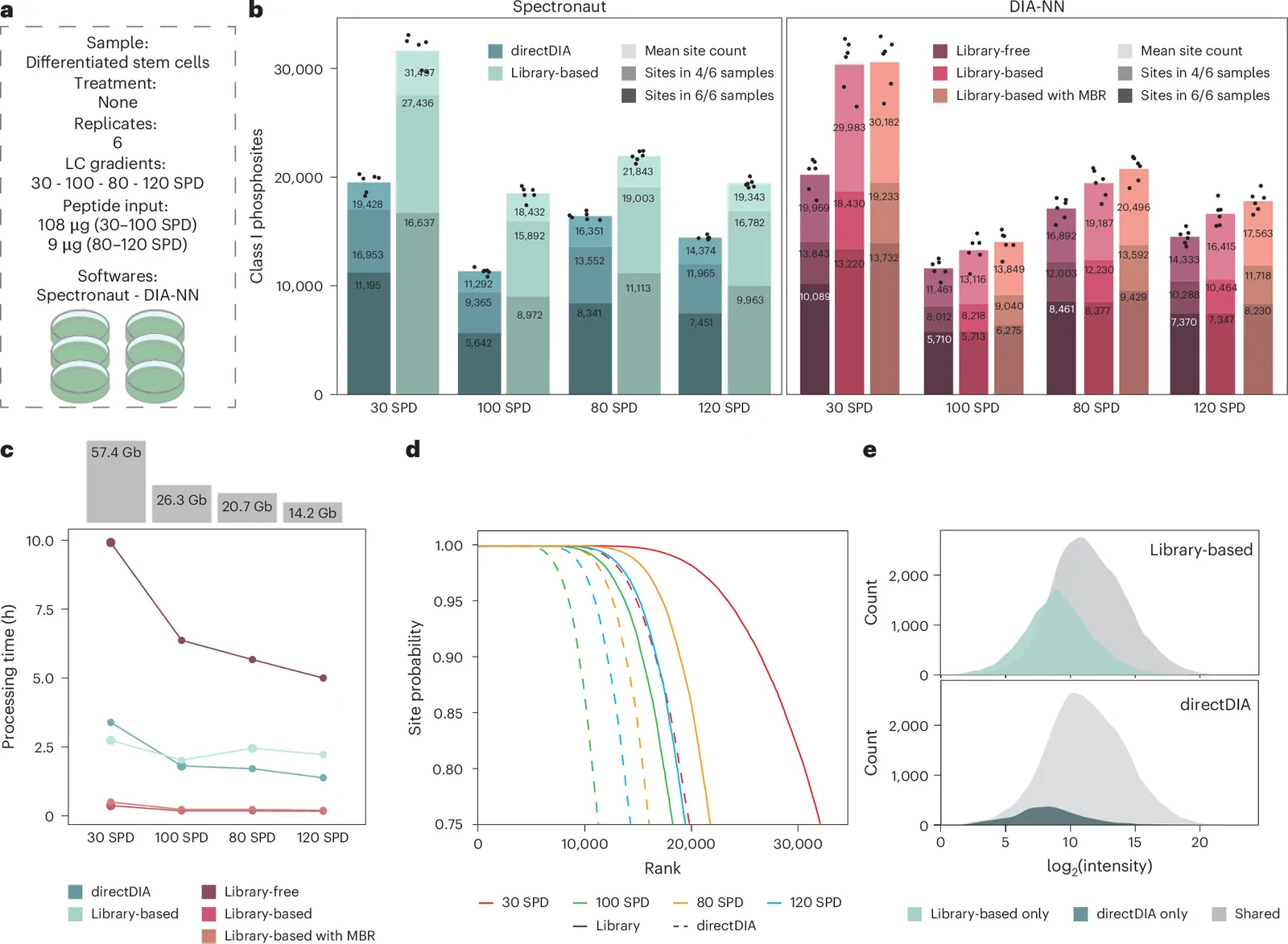
The library-based approach increases phosphosite coverage while reducing computational search time. **a**, Overview of the experimental design where differentiated stem cells were analyzed on different LC gradients. **b**, Class 1 phosphosite coverage on four different gradients (30, 100, 80 and 120 SPD). The dots represent the site count per replicate. The lightest bar displays the mean site count across the six replicates, the second bar displays the site count after filtering for 4/6 valid values and the darkest bar shows the site count with full completeness across the replicates. **c**, Line plot displaying the processing time of the searches carried out. The gray bars represent the total file size of the six replicates searched. **d**, Rank plot of the quantified phosphosites based on their localization probabilities using Spectronaut. Each color stands for a different LC gradient, while the full lines represent search outputs using the phosphopeptide spectral library, and the dashed line displays the search results from the directDIA approach. **e**, Intensity distribution plots using the 30 SPD experiment searched with Spectronaut. Source data

To evaluate the utility of our large-scale empirical spectral phosphopeptide library, we benchmarked its performance on independent samples. We generated proteome and phosphoproteome datasets from a heterogeneous pool of cells derived from an uncontrolled differentiation of iPS cells, therefore not represented in the library. Six technical replicates were analyzed by nDIA across four chromatographic conditions: high-flow gradients of 30 SPD (matching the library conditions) and 100 SPD, as well as low-flow whisper zoom gradients of 80 SPD and 120 SPD (Fig. 3a). All datasets were searched in both Spectronaut and DIA-NN, comparing empirical spectral library-based searches with the corresponding library-free approaches.

**Extended Data Fig. 4:**
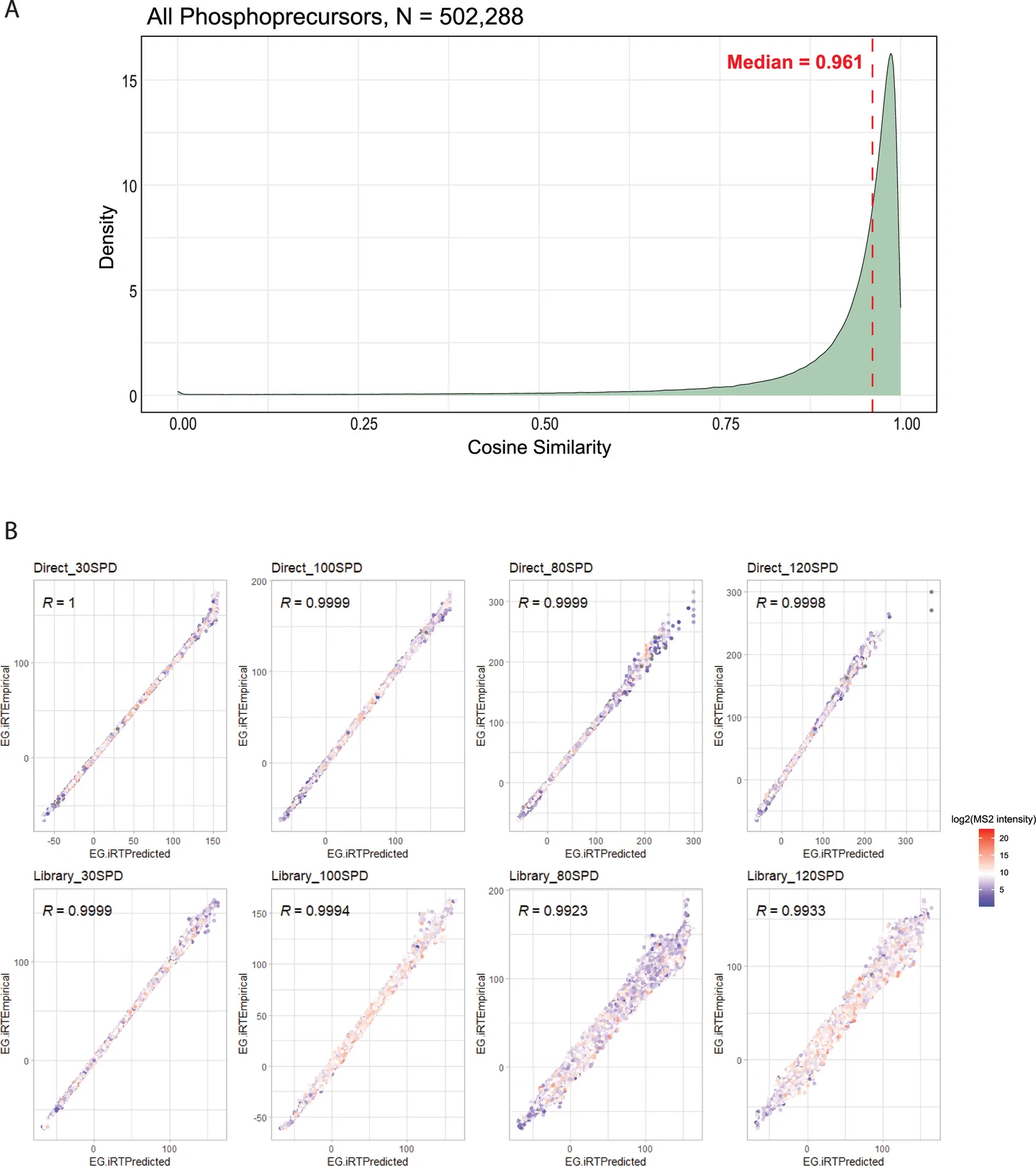
Spectral predictions and retention time predictions of phosphopeptides show high correlation with the empirical phosphopeptide library. **A**- Density plot displaying the cosine correlation spectral angle between the intensities of fragment pairs per precursor between both spectral libraries. The median spectral angle across all 502,288 phosphoprecursors is displayed in red. **B**- Retention time correlation between measured retention times and predicted retention times. In the case of directDIA, the predicted retention times are generated during the Pulsar search on the data itself. In the library-based approach, the predicted retention times are indexed retention times from the library, which was generated using 30 SPD. Spearman correlation coefficients were calculated and are displayed on the plot. The color represents the log2 MS2 intensity.

The use of the empirical phosphopeptide library increased phosphoproteome depth compared with library-free approaches regardless of gradient length and search engines, both before and after filtering of the dataset for completeness (Fig. 3b). In Spectronaut, of the use of our deep experimental spectral library led to a 60% increase in quantified phosphosites under high-flow LC conditions and a 40% increase under low-flow LC conditions when requiring ≥4/6 valid values. In DIA-NN, the empirical library mainly improved the median number of quantified phosphosites, whereas the gain after filtering for completeness was minor (1–2.5%). The close agreement between DIA-NN empirical and in silico searches probably reflects the similarity of the underlying search strategies and the high quality of the spectral predictions for phosphoproteomics displaying high similarity with empirical spectra (Extended Data Fig. 4a).

The largest gains from the empirical library were obtained when the LC gradients matched those used for library generation (up to 30% increase), probably owing to improved retention time alignment (Extended Data Fig. 4b). Nevertheless, when applied across different LC gradients, retention time correlations remained high (>0.99), supporting the broader applicability of the library. Overall, Spectronaut provided more phosphosites quantified compared with DIA-NN, which can be attributed to inherently different search strategies and site localization algorithms but also to different FDR controls and cutoff metrics.

We compared library-based and library-free computational search times in Spectronaut and DIA-NN (Fig. 3c). In DIA-NN, the empirical library resulted in a drastic reduction in search time, correlating with dataset size. Processing time for the six 30 SPD files decreased from 9 h and 55 min to just 22 min. Across all gradients, search time decreased by ~30-fold on average, reflecting the reduction in spectral library size from 46,686,794 precursors in the in silico-generated phosphopeptide library to 539,880 in the empirical spectral library. In Spectronaut, library use slightly increased processing time for small datasets but became beneficial as the dataset size increased. This benefit might be more pronounced with larger datasets, where more files would enable the generation of a larger, more comprehensive Pulsar spectral library, probably reducing the identification gap between library-based and directDIA. Overall, the empirical phosphopeptide library improved both quantification depth and computational efficiency across search strategies.

The empirical spectral library increased depth primarily through well-localized phosphosites (Fig. 3d). Using 30 SPD, directDIA, with a site localization score of 0.75, quantified approximately as many phosphosites as at the library-based search with a cutoff of 0.98, consistent with more confident site localization from high-quality spectra. This suggests that a key limitation of the directDIA approach is the difficulty in confidently localizing PTM sites. Furthermore, unique sites quantified using the empirical library tend to be of low abundance (Fig. 3e). Therefore, we can hypothesize that library-based searches could enhance phosphosite identification and localization in experiments with limited input amounts.

### The library provides increased depth in sensitive phosphoproteomics experiments

**Fig. 4:**
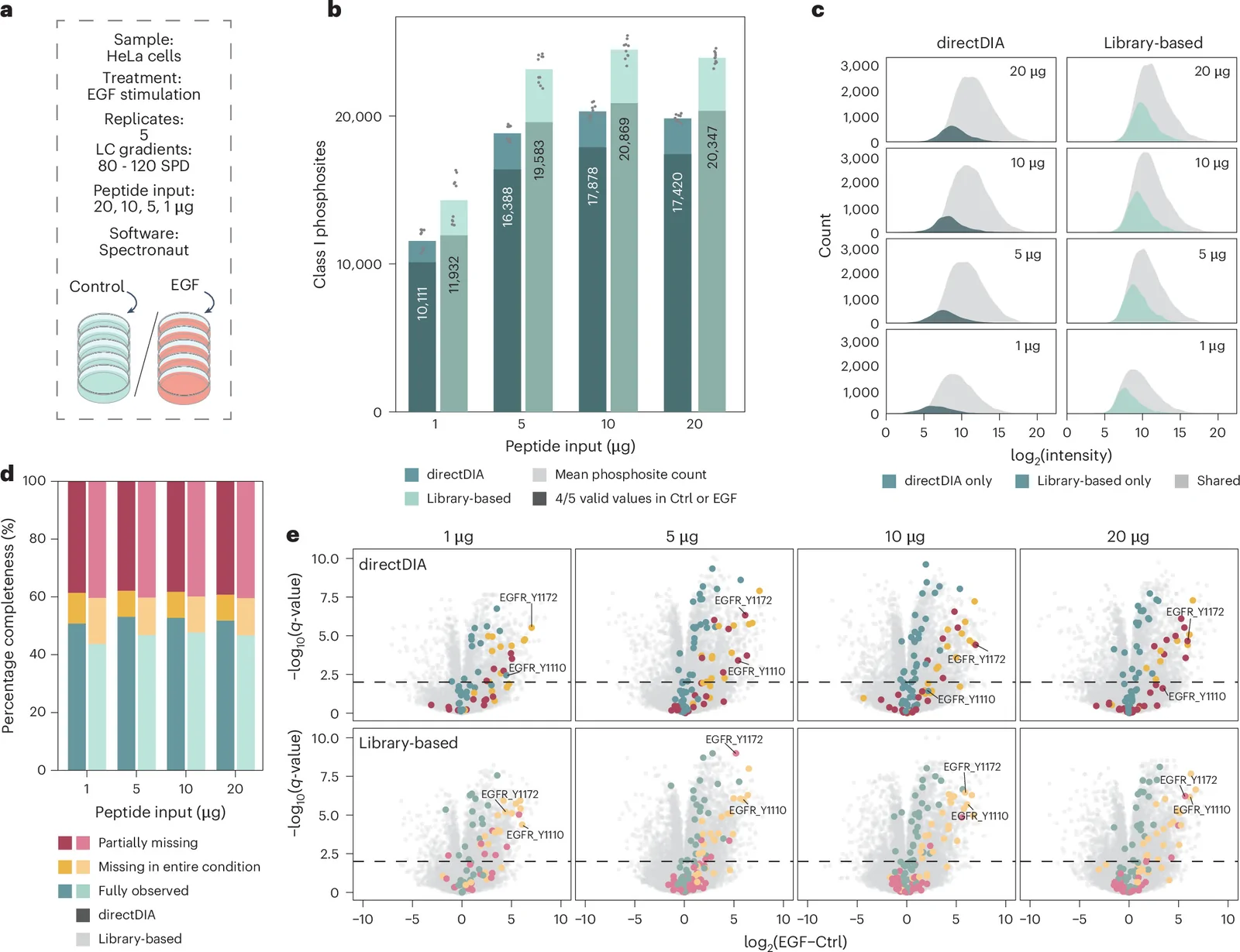
Using a comprehensive phosphopeptide spectral library improves the depth in sensitive phosphoproteomics. **a**, Overview of the experimental design of the EGF-stimulation scale down on HeLa cells. **b**, Class 1 phosphosite coverage on peptide input amount ranging from 1 to 20 μg. The dots represent the site count per replicate. The lightest bar displays the mean site count across the five replicates in the control and EGF-stimulated conditions (*N* = 10), and the darkest bar shows the site count after valid value filtering for 4/5 valid values in the control or stimulated experiments. The number displayed corresponds to the site count after filtering. **c**, Intensity distribution through the scale down. **d**, Distribution of the type of data missingness during the scale down in directDIA and using the spectral library. Fully observed values are phosphosites with valid values in all replicates in the control and EGF-stimulated experiments (*N* = 10). Missing in an entire condition is defined by zero or one valid value in the control or the EGF-stimulated experiment and four or five valid values in the other condition. All other phosphosites are annotated as partially missing. **e**, Volcano plots across the scale down for both search approaches tested. Left censored imputation was followed by statistical analysis using a two-sided Student´s *t*-test with Benjamini–Hochberg FDR correction. Color points represent phosphosites of the canonical EGFR pathway. Each color represents the type of missingness assigned to the site in the given experiment (see legend in **d**). Horizontal dotted black lines represent a cutoff at 1% FDR. Source data

**Extended Data Fig. 5:**
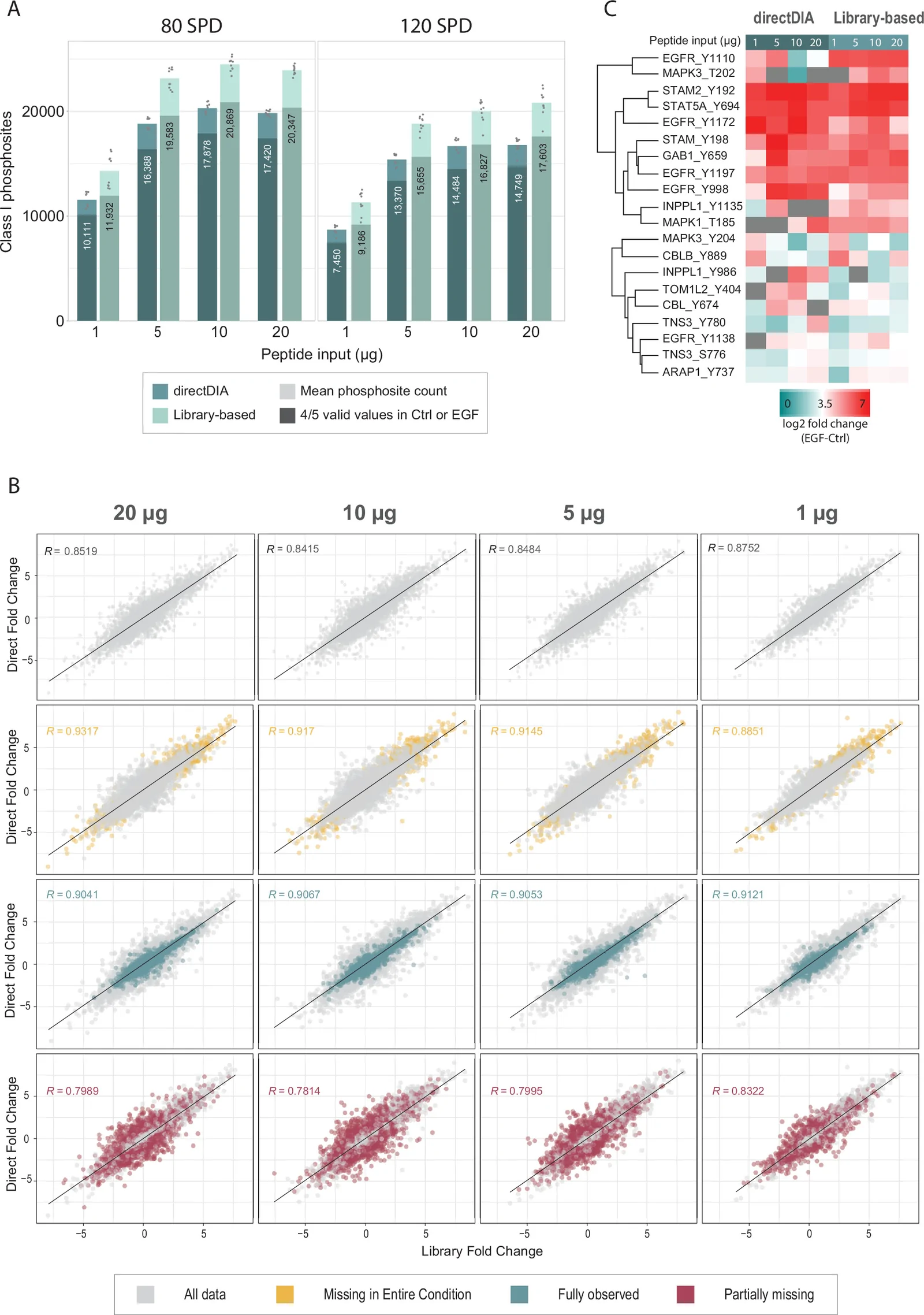
Performance of the spectral library approach compared to directDIA in a scale down. **A**- Class 1 phosphosite coverage on peptide input amount ranging from 1 to 20 μg, using the 80 SPD and the 120 SPD gradients. The dots represent the site count per replicate. The lightest bar displays the mean site count across the 5 replicates in the control and EGF stimulated conditions (N = 10), and the darkest bar shows the site count after valid value filtering for ⅘ valid values in the control or stimulated experiments. The number displayed corresponds to the site count after filtering. **B**- Heatmap of the main upregulated phosphosites in the EGFR canonical pathway. **C**- Correlation between the calculated fold changes between the EGF stimulated condition and the control, in directDIA and using the empirical spectral library. Spearman correlation coefficient is calculated per input amount and per phosphosite population: missing in entire condition, fully observed or partially missing.

Next, we assessed the potential of the empirical phosphopeptide spectral library to improve coverage of low-input phosphoproteomics experiments. We analyzed EGF-stimulated HeLa cells using peptide input amounts ranging from 1 to 20 μg followed by Ti-IMAC-enrichment and analyzed the resulting phosphopeptide mixtures by nDIA with the 80-SPD and 120-SPD whisper-zoom LC gradients to maximize sensitivity^33^ (Fig. 4a). Across all input amounts, library-based searches yielded deeper phosphoproteome coverage, both in terms of mean number of phosphosites per condition and number of phosphosites consistently quantified, with an average increase of 18% across all conditions (Fig. 4b and Extended Data Fig. 5a). Notably, coverage saturated at 10 μg peptides on the 80-SPD gradient, with consistent quantification of over 20,000 class 1 phosphosites.

In the scale-down experiment, unique phosphosites in both library-based and library-free searches were generally of low abundance, with a greater number of unique sites detected using the library (Fig. 4c). In directDIA, the intensities of unique phosphosites followed a normal distribution extending beyond the range of shared values. Quantification was largely consistent between approaches, with a Spearman correlation of ~0.85 (Extended Data Fig. 5b), although differences in the fragments used for quantification may explain minor variation.

**Extended Data Fig. 6:**
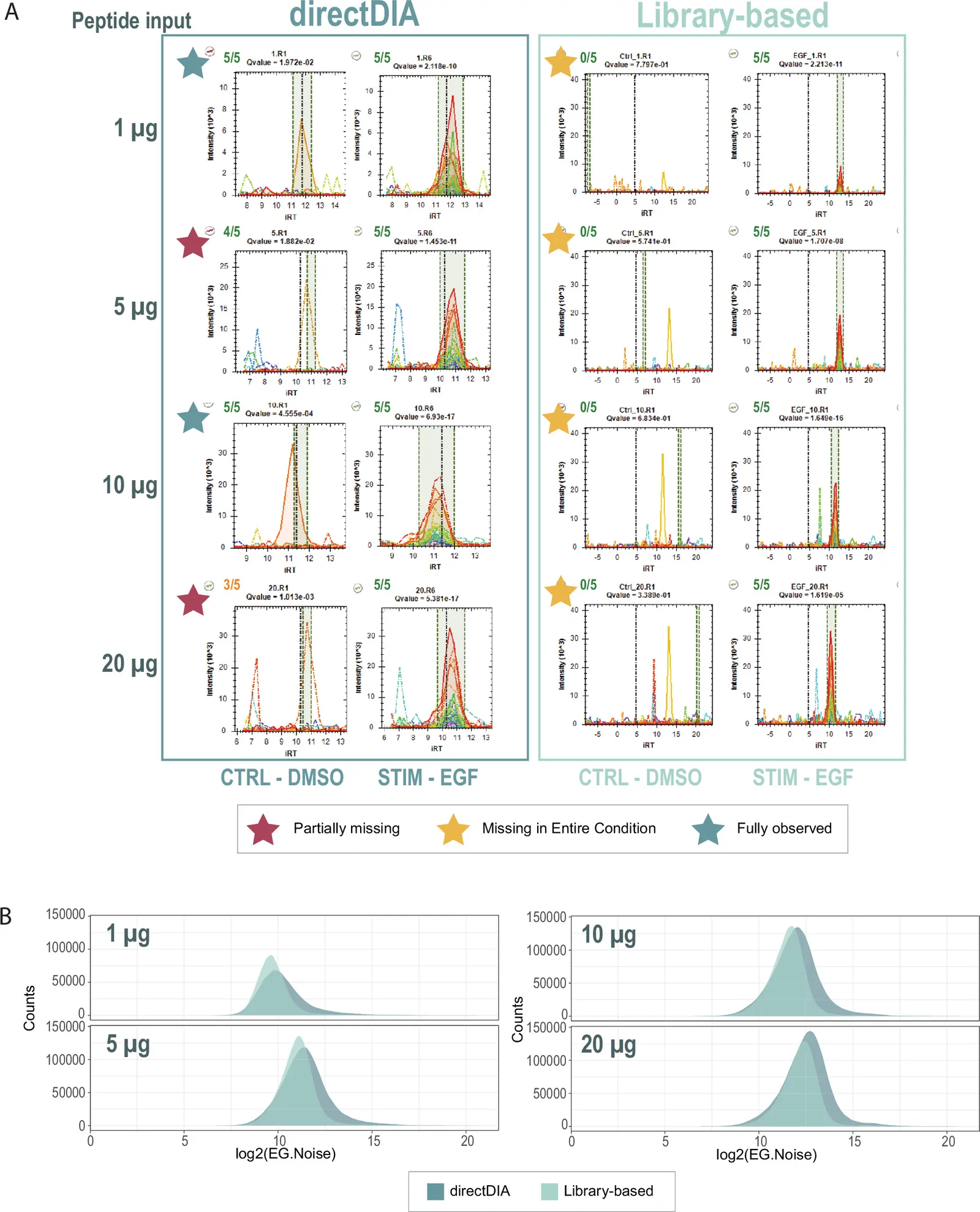
DirectDIA results in the integration to noisier signals compared to a library-based approach. **A**- MS2 traces of the main precursor covering EGFR_Y1110 in both the control and EGF stimulated conditions searched with directDIA or our spectral library. The colored stars represent the type of missingness of EGFR_Y1110 (partially missing, missing in an entire condition or fully observed). EGFR_Y1110 is an activation site and should only be detected phosphorylated after EGF stimulation. The green value represents the number of replicate out of 5 in which the site was quantified. **B** - Distribution of the log2 transformed estimated XIC noise level, calculated as the average sum XIC intensity outside of the peak boundary, between directDIA and the library-based approach. While the noise level scales with the input amount, the estimated noise level distribution is wider and spreading on the higher range in directDIA vs. library-based.

DirectDIA showed greater apparent data completeness (Fig. 4d), whereas library-based searches detected more sites considered missing in a full condition, in line with EGF-stimulated cells. While both approaches successfully identified canonical EGFR activation sites (Fig. 4e and Extended Data Fig. 5c), the library-based searches provided more consistent detection of activation sites at lower input amounts. For example, the EGFR autophosphorylation site Y1110 was either fully or partially observed in directDIA, whereas the library-based search detected the site in all stimulated replicates and none of the controls. This suggests that higher completeness in directDIA reflects noisier integration or differences in cutoff strategies in low-input phosphoproteomics, with overall lower scoring precursors leading to lower score cutoffs (Extended Data Fig. 6).

Overall, this analysis showed that the empirical spectral library enhances the depth and site localization confidence in low-input phosphoproteomics experiments and provides a rapid alternative when sample-specific deep libraries cannot be generated.

### Site stoichiometry calculation draws a clear picture of early signaling events on serum stimulation

Determining not only the relative quantification but also the occupancy or fractional stoichiometry of a phosphosite is highly informative^34^, as it integrates both phosphorylated and unphosphorylated forms and therefore offers a complementary context allowing for biological interpretation, functional analysis and systems-level modeling. We therefore aimed to calculate phosphosite stoichiometry^16,35–38^. Stoichiometry calculations from phosphoproteomics require high depth and data completeness, with both the phosphorylated and nonphosphorylated forms of a peptide detected in control and stimulated conditions

**Fig. 5:**
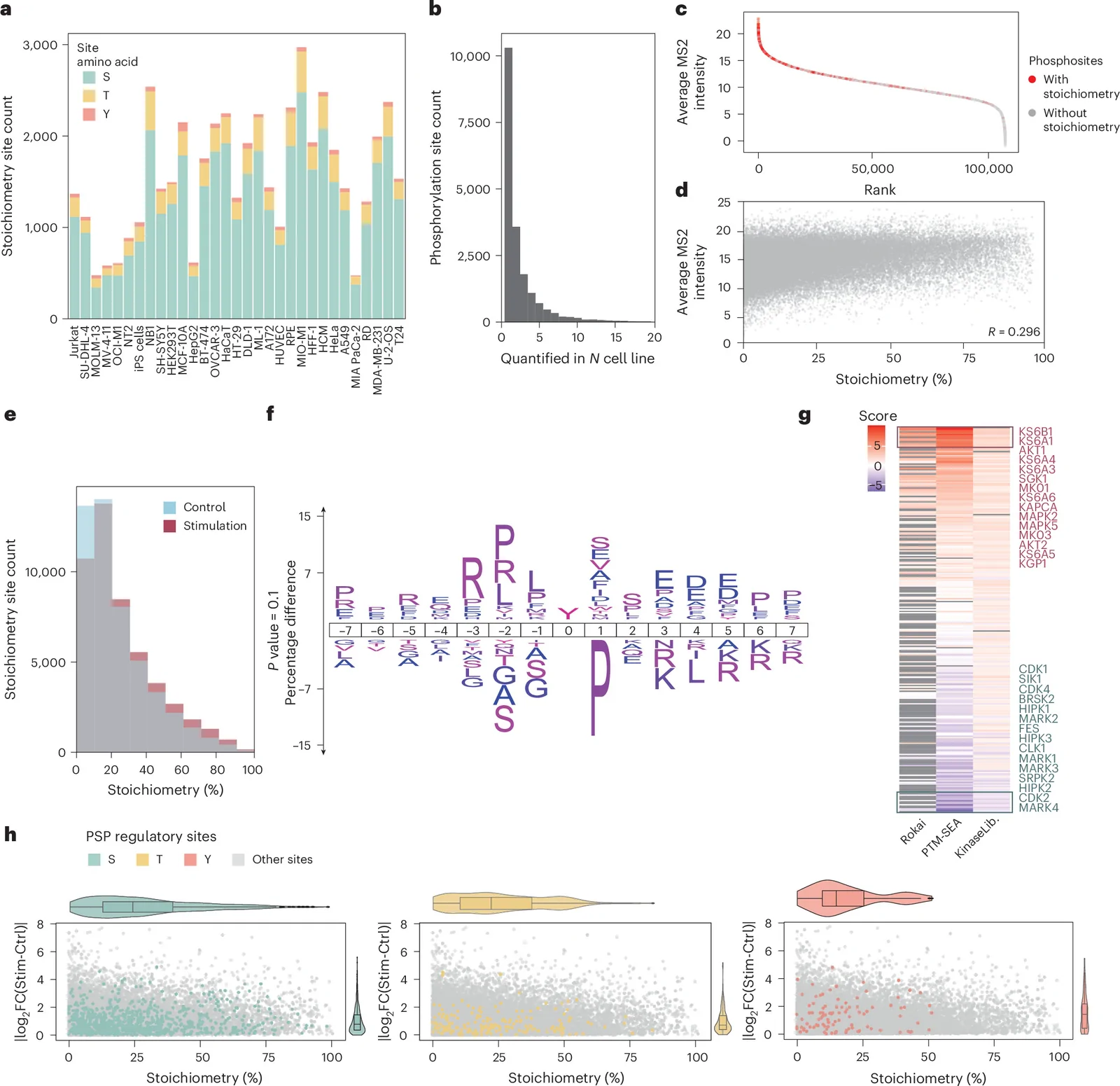
Stoichiometry calculation enables a clear overview of early signaling events on serum stimulation. **a**, Overview of the stoichiometry site count per cell line in the serum-stimulated condition relative to the control. **b**, Histogram representing the overlap between calculated sites across cell lines, where the *x* axis represents the number of cell lines across which a given site had its stoichiometry calculated. **c**, Rank plot of the measured average MS2 intensity across all cell lines per phosphosite. Sites where stoichiometry could be calculated are displayed in red. **d**, Correlation between the average MS2 intensity per cell line and the corresponding calculated site stoichiometry for the given cell line with Spearman correlation coefficient displayed on the graph. **e**, Distribution of the computed stoichiometry values in the control and serum stimulated conditions. **f**, Comparison of the phosphorylated sites (top plot) and the dephosphorylated sites (bottom plot) on serum stimulation. The statistics were generated by IceLogo^76^ using a position specific two-sided *t*-test. **g**, Heat map of the kinase activity analysis computed using Rokai, PTM-SEA using the modified iKiP-DB and the Kinase Library. Scores were calculated on the basis of the difference between the stoichiometry in the serum-stimulated condition and the control. Gray squares represent kinases where the activity could not be inferred. Kinases displayed in red represent the top 15 most activated kinases and the ones in blue represent the top 15 most inactivated kinases. **h**, Absolute value of the intensity-based FC between serum stimulation and control as a function of the stoichiometry value in the stimulated condition (pS: *n* = 15,443, pT: *n* = 3,109, pY: *n* = 826). Known regulatory sites from PSP were colored on the basis of the modified amino acid. Violin plots represent the overall distribution of the stoichiometry (horizontal violins) or the absolute values of the intensity-based FCs (vertical violins) for the regulatory sites (pS: *n* = 903, pT: *n* = 196, pY: *n* = 103). Box plots show the median (center line), the first and third quartiles (box limits) and the minimum and maximum values (whiskers). Source data

**Extended Data Fig. 7:**
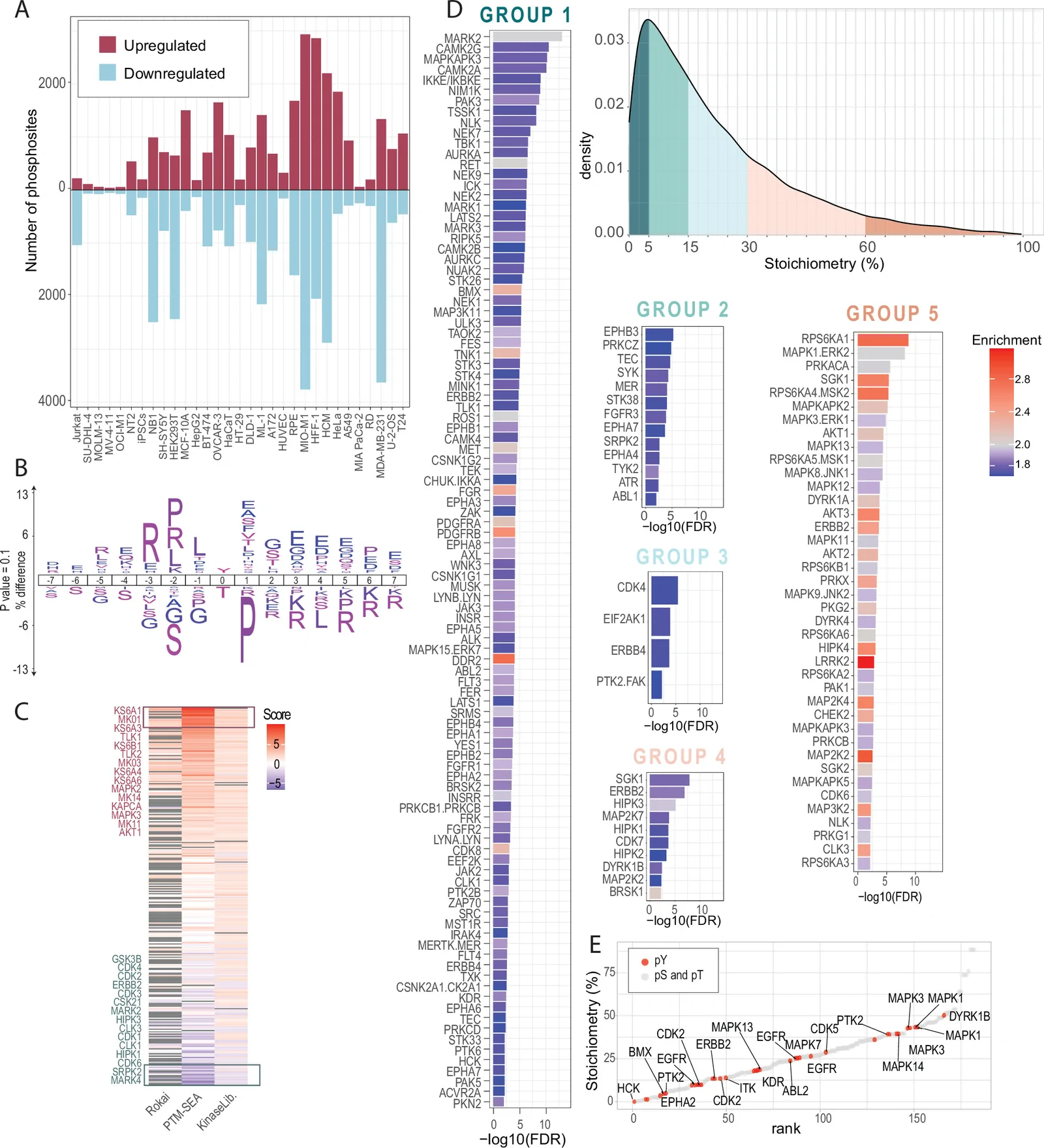
Site stoichiometry analysis recapitulates intensity-based results while providing absolute phosphorylation levels and revealing whether kinases drive high- or low-stoichiometry substrate phosphorylation. **A**- Up- and down-regulated phosphosite count per cell line upon serum stimulation using site based intensity values. Sites with Benjamini Hochberg corrected FDR values below 1% after limma statistical testing were considered statistically significantly regulated. **B**- Motif analysis of the sequences around significantly up-regulated (top) and down-regulated (bottom) sites. The statistics were generated by IceLogo using a position specific two-sided t-test. **C**- Kinase activity analysis summary using site intensities of regulated sites, using Rokai, PTM-SEA with the modified iKiP-DB and the Kinase Library. Kinases scored in 2 out of 3 tools are displayed in the heatmap. **D**- Overall distribution of calculated site stoichiometries in the stimulated conditions divided into stoichiometry bins. Group 1 spans up to 5%, group 2 ranges from 5 to 15%, group 3 from 15 to 30%, group 4 from 30 to 60% and group 5 above 60%. Sites were annotated with the modified iKiP-DB kinase substrate relationships and over representation analysis using Fisher exact test was carried out per group. Displayed results are filtered for 1% FDR, enrichment scores > 1.7 and at least 7 substrates for a kinase were required per group. **E**- Rank plot of the stoichiometries calculated in the stimulated condition in 182 activation sites. pY are labelled in red. Source data

Stoichiometry was calculated for 31 cell lines, excluding SCC-25 and PC-3, which showed mycoplasma contamination, reflecting the effect of serum stimulation^16^. On average, stoichiometry was calculated for 1,580 phosphosites per cell line spanning a wide range from 474 to 2,970 sites, reflecting variability in cell stimulation efficiency and responsiveness (Extended Data Fig. 7a). In total, stoichiometry was determined for 19,378 sites encompassing 15,443 pS (80%), 3,109 pT (16%) and 826 pY (4%) (Fig. 5a). The stoichiometry for a majority of sites was cell-line specific (Fig. 5b), probably owing to missingness arising from the requirement to match phosphoproteome and proteome data at the modified peptide level, where most stoichiometry-quantified sites corresponded to phosphopeptide with high MS2 intensities (Fig. 5c). Across all cell lines, stoichiometry and phosphopeptide intensity showed only a weak positive correlation, indicating that the two metrics capture distinct aspects of the data (Fig. 5d).

Occupancies were generally low (<20%) and increased on serum stimulation, with a distribution shifted toward higher occupancy values (Fig. 5e). Phosphosites with a positive fold change (FC, phosphorylated) were enriched in basophilic sequence motifs with an overrepresentation of arginine in positions -3 and -2, characteristic for substrates of kinases from the AGC family, including PKA, PKB or S6K (Fig. 5f). A moderate enrichment in tyrosine phosphorylation was also observed. By contrast, dephosphorylation motifs were enriched in proline-directed phosphosites with proline at position +1, typically targeted by MAPKs and CDKs, indicating phosphatase-mediated deactivation of these pathways. Kinase–substrate enrichment analysis was performed on stoichiometry FCs between stimulation and control (Fig. 5g). The top 15 most serum-activated kinases identified by three independent kinase inference algorithms included ribosomal protein kinases (KS6B1 and KS6A1/3/4/5/6), PKB (AKT1/2) and MAPK (MAPK1/2/3/5). Conversely, the most strongly deactivated kinases implicated by substrate dephosphorylation were MARK family members responsible for the phosphorylation of microtubule-associated proteins, promoting microtubule dynamics^39–41^. Moreover, cell cycle-regulating CDKs (CDK1/2/4) were also among the most inactivated kinases.

While performing this analysis on intensity-based FCs led to similar conclusions (Extended Data Fig. 7a–c), stoichiometry provides an absolute measure of site occupancy, serving as a resource for the basal phosphorylation state of sites within a cell. iKiP-DB kinase–substrate relationships and overrepresentation analysis showed that most kinases, including tyrosine and Aurora kinases, phosphorylate substrates to low occupancy, whereas MAPK, RSK and CDK substrates could reach higher occupancies (Extended Data Fig. 7d). Nevertheless, substrates of a given kinase could still span multiple stoichiometry bins. Focusing on regulatory sites from PSP, pS sites showed the highest occupancies while pY sites showed the lowest despite displaying the largest FCs (Fig. 5h), confirming that only a small fraction of pY in a protein is needed to drive crucial downstream signaling. This also supports that regulatory pY has very low baseline occupancy, which explains why antibodies are often needed for specific enrichment of tyrosine-phosphorylated peptides for global profiling by LC–MS^42^. This deep phosphoproteome resource serves as a baseline for understanding site-specific occupancy threshold activating signaling events, especially with site stoichiometries calculated for 182 activation sites and 1,202 regulatory sites (Extended Data Fig. 7e).

### Cscore calculation allows for kinase activity landscapes across cell lines

**Extended Data Fig. 8:**
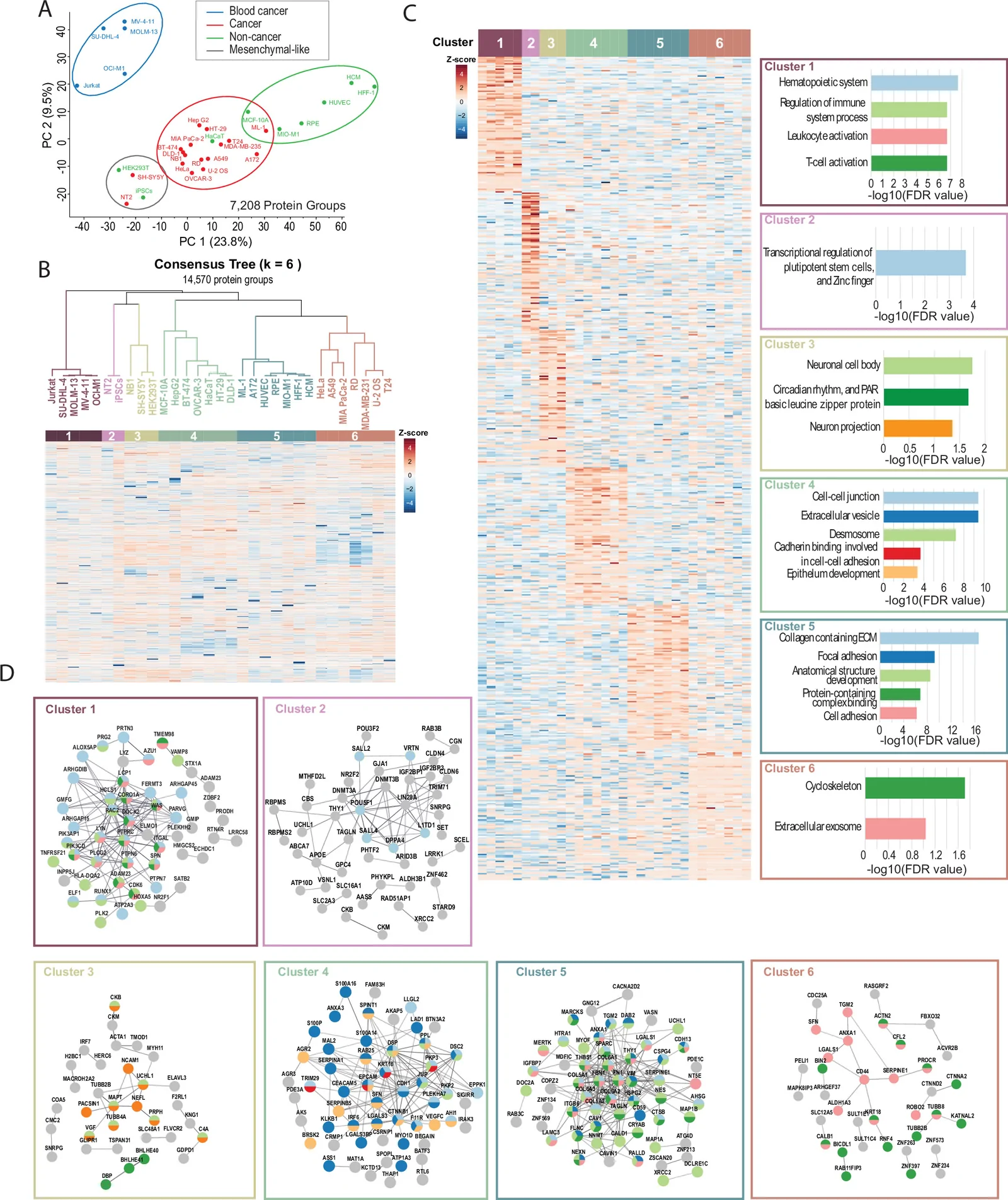
Functional analysis across cell lines shows distinct clusters. **A**- PCA plot based on the proteome data filtered for full completeness. **B**- Cell line clustering using consensus clustering on 14,570 quantified protein groups. Missing values were imputed using KNN. **C**- Heat map with the top 100 protein groups per cluster that were the most differentially overexpressed compared to the background of all protein groups. The results on the gene set enrichment of those proteins were carried out on Cytoscape. **D**- Network analysis of the top 100 protein groups per cluster. Color coded proteins on the network are involved in the corresponding color coded pathway or GO term in panel **C**. Source data

Functional proteome analysis across cell lines revealed six distinct lineage-associated clusters, separating blood cancer, pluripotent, neuronal, noncancerous and cancer cell lines with low or high epithelial phenotypes into discrete groups (Extended Data Fig. 8). We next attempted to infer kinase activity patterns, essential to translate phosphosite-level changes into functional signaling insights by identifying the kinases underlying the observed dynamics^43–45^. This is particularly relevant in cancer research, where aberrant kinase activities often drive tumor progression and represent promising therapeutic targets^46^.

**Fig. 6:**
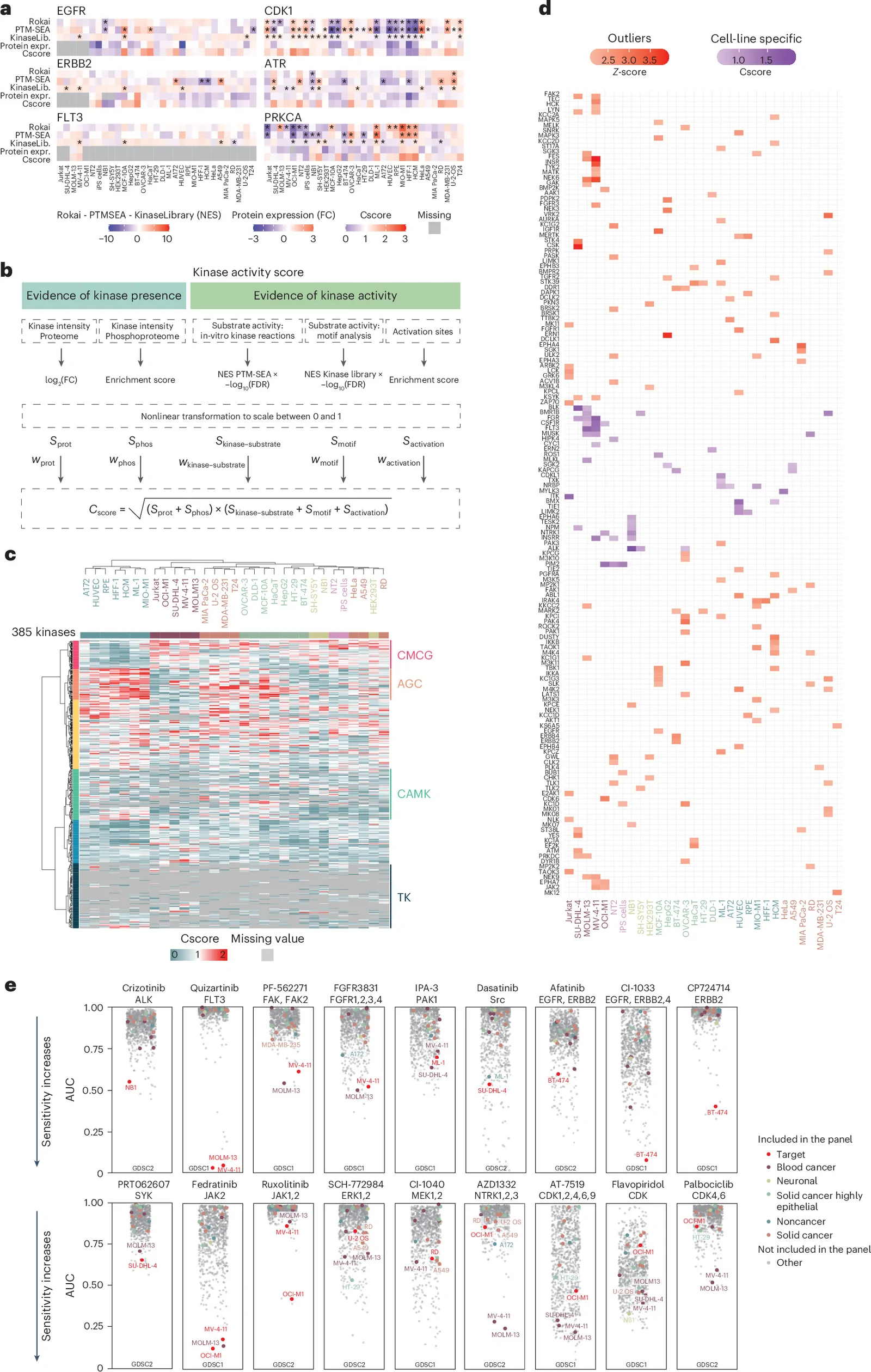
A combined kinase score allows to identify cell line-specific kinase activity landscapes. **a**, Heat maps displaying the inferred kinase activity calculated across cell lines with RoKai, PTM-SEA using the iKiP-DB, the Kinase Library, the kinase protein expression and the Cscore for six kinases, including three tyrosine kinases (left). The stars represent statistical significance at 5% FDR. **b**, Flow chart of the Cscore. **c**, Heat map of the Cscore across cell lines. **d**, Heat map displaying the kinase–cell line associations. The red squares represent associations established by identifying kinase activity outliers, defined as cell lines with activity outside of the normal distribution of that given kinase across all cell lines within a 95% confidence interval (that is, *Z*-score >2). Purple squares display associations defined by cell line-specific kinase activity, where Cscore is computed in four cell lines or less. **e**, Drug-sensitivity screen data from GDSC1 or GDSC2 over different kinase inhibitors. Each red dot represents a kinase–cell line association. Colored dots represent cell lines in the panel on which the kinase inhibitor was tested, and gray dots show other cell lines in the drug test panel. The name of the kinase inhibitor is on the top row, and the targeted kinase(s) are on the bottom row. Source data

Kinase activity analysis typically relies on the detection of phosphosites on downstream substrates using established kinase–substrate relationships or kinase-specific motif patterns^28,47–53^. These approaches remain challenging for closely related kinases, particularly tyrosine kinases, that share a large proportion of their downstream substrates^49,54^. In such cases, computational kinase activity inference analysis tools such as Rokai^51^, PTM-SEA^28,29^ or the Kinase Library^54^ often lack resolution, resulting in false positive signals (Fig. 6a). All three assigned EGFR activity to blood cancer cell lines despite absent EGFR protein expression in nonepithelial cells, in some cases even assigning positive activity, implying higher EGFR activity compared with the background of all cell lines. Likewise, ERBB2, known to be expressed in epithelial cells with a high protein abundance level in ERBB2-positive breast cancer cell lines such as BT-474, obtained activity scores in blood cancer cell lines and failed to identify significantly higher ERBB2 activity in BT-474. Finally, FLT3, expressed in hematopoietic progenitor cells and constitutively active in MOLM-13 and MV-4-11 owing to a FLT3 internal tandem duplication mutation^55^, did not show higher kinase activity in those cell lines. (Fig. 6a).

**Extended Data Fig. 9:**
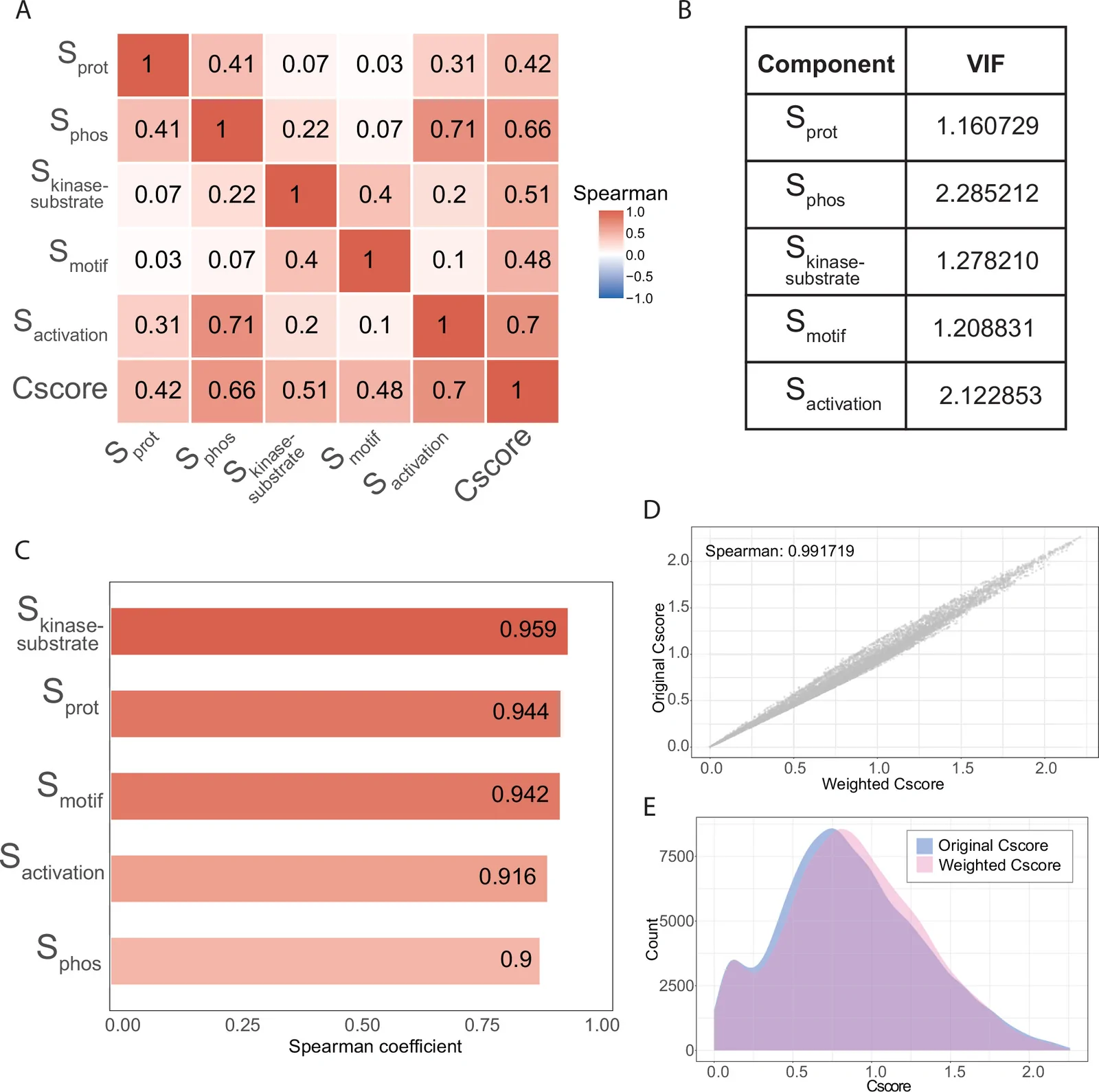
Validation of the independence of the Cscore components. **A** - Pairwise spearman correlation coefficients between each components of the score across all cell lines and kinases. **B** - Variance Inflation Ratio (VIF) computed per score component for the detection of multicolinearities. **C** - Spearman correlation coefficient between the original Cscore and a newly computer score excluding one component at a time. **D** - Correlation plot between the weighted and unweighted Cscores. **E** - Cscore distribution for the original and weighted Cscores.

To fully exploit the depth of our dataset and obtain a comprehensive overview of kinase activities across the cell lines, we developed and applied a combined kinase activity score (Cscore) by integrating information from the paired pan-proteome and phosphoproteome (Fig. 6b). Our Cscore combines evidence of kinase presence at the proteome and phosphoproteome levels, with evidence of the kinase activity derived from three complementary elements. Two of these scores are based on substrate phosphorylation and are directly computed through pre-existing tools: one using established kinase–substrate relationships from in vitro kinase reaction experiments^56^ complemented with PSP annotations^28,29^ (Supplementary Fig. 3) and the other based on sequence motif analysis^54^. The third activity score takes into account the quantification of activation sites on the kinase. All components of the Cscore were considered independent. However, the phosphoproteomics kinase expression level and the activation site levels showed higher correlation (Extended Data Fig. 9a–c). Consequently, the relative contribution of the components to the Cscore was corrected with weights applied on the basis of their correlations (Extended Data Fig. 9d,e). In addition, a SKEW factor^53^ was calculated representing the relative contribution of the presence or the activity components in the Cscore, which was efficient for detecting kinases that were expressed but inactive following kinase inhibition (Supplementary Information 1–3).

Altogether, we computed kinase activity scores for 385 kinases across 31 human cell lines (Fig. 6c). The Cscore provides improved resolution for tyrosine kinases, with missing values appropriately assigned to kinases not expressed. Importantly, the Cscore correctly scores the directionality of a kinase activity state in individual cell lines, as demonstrated with the zero activity score of both EGFR and ERBB2 in suspension cells and the high activity score of ERBB2 in BT-474 (Fig. 6a). For non-tyrosine kinases, the Cscores agreed with previously assessed kinase activity using existing tools in both scale and directionality. For example, CDK1 and ATR showed higher activity in cancer cell lines compared with noncancerous cell lines.

### Cscore provides insights into cell sensitivities and therapeutic options

We next attempted to identify kinase-specific therapeutic vulnerabilities, defined as key proteins for a cell´s growth and survival and validate these hypotheses using large-scale drug screening data from the Genomics of Drug Sensitivity in Cancer (GDSC) project^57,58^. We hypothesized that cell lines with high activity of a given kinase would be more sensitive to inhibitors targeting that kinase^59^.

Cell line-specific sensitivities were investigated through kinase–cell line pairs in which a kinase in a given cell line either showed activity substantially higher than its activity distribution across all other cell lines, or it was considered cell line-specific when it was active in four or fewer cell lines, resulting in 210 kinase–cell line associations (Fig. 6d). We evaluated cancer cell lines and kinases with available and specific small molecule ATP-mimetic inhibitors and compared the corresponding area under the curve (AUC) values from the GDSC dataset.

Several of the identified kinase–cell line associations matched known drug sensitivities (Fig. 6e). For instance, NB1 (neuroblastoma) with a high ALK activity score exhibited high sensitivity to ALK inhibition, and cell lines with active FLT3 (MOLM-13 and MV-4-11 cell lines) were sensitive to FLT3 inhibitors. Moreover, MV-4-11 cells responded to FAK and FGFR inhibition, ML-1 cells to PAK1 inhibition and SU-DHL-4 cells to Src and SYK inhibition. Notably, high activities of ERBB2 and ERBB4 were identified as outliers in BT-474 cells. BT-474 cells were sensitive to Afatinib, an EGFR and ERBB2 inhibitor. When treated with CI-1033, which additionally targets ERBB4, BT-474 cells showed increased sensitivity. By contrast, selective inhibition of ERBB2 reduced overall sensitivity but improved specificity, with only a few cell lines, including BT-474, showing a response. Similarly, MAPK pathway inhibitors (ERK, MEK and JNK) showed strong effects in U-2 OS and RD cell lines, which exhibited among the lowest AUCs.

**Extended Data Fig. 10:**
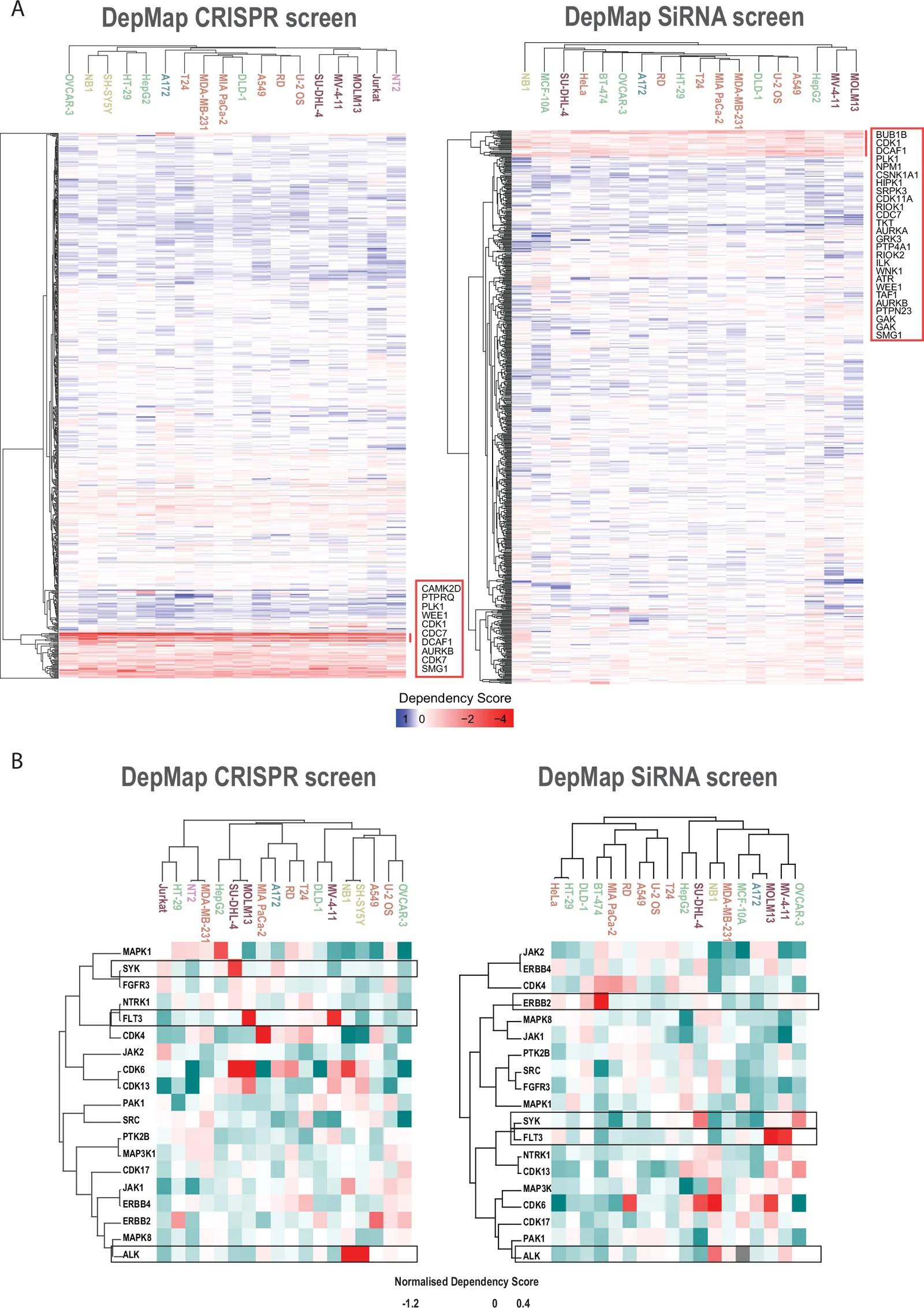
Validation of the functional vulnerabilities. **A**- Dependency scores from DepMap (CRISPR screens on the left and SiRNA screens on the right) show that all cell lines are highly dependent on the same kinases for survival. **B**- Dependency scores from DepMap (CRISPR screens on the left and SiRNA screens on the right) after median subtraction shows higher dependencies for some of the hypothesized associations. Source data

Some kinase–drug relationships were less straightforward. Although OCI-M1 cells showed high NTRK1 activity, NTRK inhibitors were more effective in other cell lines, including MOLM-13, MV-4-11, A172 and A549, and showed limited sensitivity in OCI-M1. Predicted CDK6 sensitivity in the OCI-M1 cell line was also not selectively validated. Broad-spectrum CDK inhibitors affected multiple cell lines, whereas highly specific CDK4/6 inhibitors had limited effects and nonselectively affected OCI-M1 cell viability. This highlights both the challenges of interpreting drug screens, particularly when drug selectivity is not well described and off-target effects can interfere with the results^60^, and inferring accurate kinase activity through phosphosite abundance changes. Overall, while most cell lines depend on core survival kinases such as PLK1, WEE1 and CDK1, DepMap data revealed differential kinase dependencies by siRNA knockdown or CRISPR knockout that enabled validation of cell line-specific vulnerabilities, including ALK in NB1, FLT3 in MOLM-13 and MV-4-11, ERBB2 in BT-474 and SYK in SU-DHL-4 (Extended Data Fig. 10).

These findings support selective inhibitors as one approach for validating Cscore-predicted vulnerabilities. Additional potential targets were identified, but lacked experimental validation. The MIA PaCa-2 cell line displayed highly specific activity of EPHA3 and EPHA4 kinases, implicated in angiogenesis. Inhibiting ephrin kinases could potentially limit pancreatic cancer cell growth, consistent with reports that high ephrin expression correlates with poorer prognosis^61^ and that EphA3 inhibition reduces tumor growth and progression in pancreatic cancer^62^. Similarly, ERN1 was an extreme outlier in the HepG2 cell line. Given its role in modulating tumor progression in colorectal cancer^63^, it could represent a potential therapeutic target in hepatic cancer.

## Discussion

In this study, we generated deep proteome and phosphoproteome profiles across a panel of 33 human cell lines representing the major tissue types. With an average of 9,700 protein groups and ~30,000 class I phosphosites quantified per cell line, this dataset constitutes a unique community resource, which can be directly used as a high-quality experimental spectral library. Notably, the proteome library combines high depth and sequence coverage, with over 16,000 protein groups detected and a median sequence coverage of 50%. Previous large-scale studies reported up to ~17,000 protein-coding genes using diverse approaches such as complementary proteases or extensive offline peptide fractionation^64,65^. By contrast, our dataset was generated with tryptic peptides using single-shot LC–MS/MS analysis. The remaining gap to the ~20,000 human canonical proteins may reflect limits in acquisition depth, experimental design or genuinely undetectable regions of the human proteome. With more than 200 different cell types in the human body, our dataset will also lack cell type-specific proteins from cells that were not part of our panel. For example, the mammalian genome encodes up to 1,000 odorant receptors, which is a large family of G-protein-coupled receptors (GPCRs) found almost exclusively on the cilial membrane of olfactory sensory neurons in the olfactory epithelium^66^. As we did not profile this cell type, GPCRs were not covered in our dataset. Moreover, the searches were performed against the canonical reference proteome, and we anticipate the dataset to be able to further reveal cell line-specific protein isoforms or mutations^67,68^.

Our phosphoproteomics profiling achieved unprecedented coverage within a single unified experiment, with >225,000 unique phosphosites detected across all cell lines and >109,827 phosphosites consistently and confidently quantified. Approximately ~90,000 of these sites overlapped with PSP, including phosphosites without previous MS evidence. High coverage of curated signaling pathways^27,28^, and inclusion of both cancerous and noncancerous cell lines, enable comparison of diverse signaling states. While our deep empirical spectral phosphopeptide library demonstrated extensive completeness, phosphorylation events were highly tissue and context specific. Therefore, phosphosites unique to tissues not included in our panel, or present on the noncannonical human proteome, or phosphosites requiring specific activations would not be detectable. In addition, accurate false discovery rate (FDR) estimation in phosphoproteomics remains challenging, in part because the full extent of the human phosphoproteome remains unknown, and comprehensive ground-truth datasets for benchmarking search tools are lacking. This limitation is becoming increasingly important as the field moves toward larger-scale studies, with the use of merged datasets, making estimation of a global FDR both more difficult and more critical. While the use of a spectral library for DIA-based phosphoproteomics was the standard approach before the emergence of directDIA^17^, our results showed that our highly complete empirical phosphopeptide spectral library can still substantially improve DIA-based phosphoproteomics, without the need for fractionation^17^. In both Spectronaut and DIA-NN, the empirical library outperformed the respective library-free approaches. DIA-NN’s library-free workflow relies on a large in silico-generated spectral library from a FASTA file, reducing it after a first-pass search, followed by feature extraction^69^. Replacing this large theoretical search space with our empirically derived library dramatically narrows the initial search space, which accounts for the modest but consistent gains in identification depth and the striking ~30-fold reduction in computational processing time. Spectronaut’s directDIA, by contrast, builds a library directly from the DIA data through an initial data-dependent acquisition-like search, and its depth therefore depends strongly on the quality and number of input files available for confident identification and localization of phosphopeptides^17,70^. In this context, applying our empirical library resulted in a substantial increase in identifications but did not reduce processing time. Given the nature of the directDIA workflow, benchmarking on a larger dataset would probably reduce the identification gap and better highlight the processing-time advantages of an empirical library.

Importantly, the empirical library improved performance in high-sensitivity phosphoproteomics experiments, allowing for an average gain of 18% phosphosites. directDIA showed apparent higher data completeness, including quantifications of sites entirely absent from the unstimulated condition but quantified in all the EGF-stimulated conditions when searched using our experimental library, such as phosphosites on kinase activation loops. This suggests that low-input performance is limited by the MS/MS quality available for confident identification and localization, while noisier integration or suboptimal score thresholds may inflate apparent completeness as input decreases^71^. Using the empirical library alleviates these issues by supplying high-quality reference spectra, thereby reducing the reliance on low-quality MS/MS. Combining the library with recent ultralow input workflows^72–74^ may further extend phosphosproteome coverage in small cell populations.

Phosphorylation site stoichiometry adds a crucial quantitative dimension to large-scale phosphoproteomics data. While phosphorylation stoichiometry provides a direct, biologically meaningful measure of signaling activity, allowing to prioritize therapeutic targets or build more accurate models of cellular signaling networks, its functional interpretation should be considered alongside other regulatory layers such as proteoform diversity, subcellular localization and protein complex context. We estimated the stoichiometry for the dynamically changing sites on 10 min of serum stimulation, which allowed for the calculation of the fractional site occupancy for more than 19,000 phosphosites in total. These results provide resolution beyond relative abundance-based phosphoproteomics serving as a resource for basal cellular phosphorylation levels.

Although stoichiometry offers a valuable complementary perspective on phosphorylation dynamics and has become more feasible with increasing data depth and completeness, the approach remains limited by data sparsity and measurement precision. Each stoichiometry value accumulates the error of four independent intensity measurements (phosphorylated and nonphosphorylated forms in control and stimulated samples), reducing the sensitivity for identifying small occupancy changes. Consequently, low-occupancy sites that double on stimulation may remain within measurement uncertainty, while mid-occupancy sites appear dynamic and lead to an occupancy calculation. As most sites exhibited low occupancy, unbiased and precise stoichiometry calculation remains a challenge. Focusing on stoichiometry at a smaller scale and with emphasis on precision, through targeted approaches for instance^38^, would allow to more precisely unveil crucial information such as site occupancy required for functional activation.

To systematically map kinase activities, we developed a combined kinase activity score, the Cscore, optimized to discriminate among kinases with shared substrates. Cscores were calculated for 385 kinases across 31 cell lines, highlighting cell line-specific kinase activities. Importantly, because the Cscore integrates multiple independent lines of evidence for both kinase presence and activity, relying on empirical kinase–substrate relationships and predicted activation sites, the level of annotation of a kinase should have minimal effect on the Cscore, especially while comparing kinases across samples. Within a sample, however, a kinase with fewer known or MS-detectable substrates could lead to lower Cscores. We further correlated these kinase-activity profiles with publicly available drug-sensitivity data and validated cell line–kinase vulnerabilities in several cell lines by their kinase inhibitor responses. Although the validation is limited because many kinase–cell line combinations are not druggable, lack a selective inhibitor or are absent from the GDSC dataset^58^, we demonstrated sensitivity on several well-established and undemonstrated associations, suggesting that further vulnerabilities could be demonstrated via drug screens. In addition to kinase activity being an indirect readout, the limited specificity of kinase inhibitors complicated direct validation, as off-target effects are common and often uncharacterized. Although specificity profiles have been reported for many kinase inhibitors^60^, they rarely overlap with available cell viability datasets. Nonetheless, this resource can support rational drug testing strategies and combination-therapy design by enabling prioritization of the most active kinases in each cell line.

Overall, we provide a versatile resource that can serve as a high-quality experimental spectral library, support data exploration and mining, guide targeted MS assay development, inform drug-sensitivity studies and help identify new therapeutic vulnerabilities.

## Methods

### Cell culture

All cell lines were cultured at 37 °C in a humidified incubator with 5% CO_2_, and all media were supplemented with 10% fetal bovine serum and 100 μg ml^−1^ penicillin–streptomycin (Invitrogen), unless specified otherwise.

A172 (CRL-1620), A549 (CCL-185), DLD-1 (CCL-221), HaCaT (CVCL_0038), HEK293T (CRL-3216), HeLa (CRL-2), HepG2 (HB-8065), HFF-1 (SCRC-1041), HT-29 (HTB-38), MDA-MB-231 (HTB-26), MIA PaCa-2 (CRL-1420), MIO-M1 (CVCL_0433), ML-1 (ACC 464), NT2 (CRL-1973), RD (CCL-136), RPE (CRL-4002), SH-SY5Y (CRL-2266), T24 (HTB-4) and U-2 OS (HTB-96) were cultured in DMEM (Gibco). BT-474 (HTB-20), Jurkat (TIB-152), MOLM-13 (ACC 554), NB1 (CVCL_1440) and OVCAR-3 (HTB-161) were cultured in Roswell Park Memorial Institute medium (RPMI; Gibco). SCC-25 (CRL-1628) and PC-3 (CRL-1435) were cultured in DMEM-F12 (Gibco). MV-4-11 (CRL-9591) and OCI-M1 (ACC 529) were cultured in IMDM (Gibco). SU-DHL-4 (CRL-2957) was cultured in RPMI with American Type Culture Collection (ATCC) modification (Gibco). MCF-10A (CRL-10317) was cultured in DMEM/F12 with glutaMAX (Gibco) supplemented with 5% horse serum (Gibco), 1% (wt/vol) nonessential amino acids (Gibco), 0.01 mg ml^−1^ insulin (Sigma-Aldrich), 100 ng ml^−1^ cholera toxin (Sigma-Aldrich), 500 ng ml^−1^ hydrocortison (Sigma-Aldrich) and 20 ng ml^−1^ EGF (Peprotech). HUVEC was cultured in Endothelial Cell Growth Media (EGM-2) (Lonza) supplemented as provided by the manufacturer. HCM (C-12810) was cultured in Myocyte Growth Media (PromoCell) supplemented as recommended by the manufacturer. Human iPS cells hi12^77^ (CRL-2429) were expended on laminin521-coated dishes in E8 medium (Gibco) supplemented with 0.1% bovine serum albumin (BSA).

iPS-cell differentiation into EBs was carried out by dissociated hi12 cells using TrypLE Express for 5 min. TrypLE was removed, and the cells were rinsed once with fresh E8 medium supplemented with 0.1% BSA, scraped, resuspended in fresh medium with 10 μM Y-27632 (ROCK inhibitor) and cultured in suspension in low-adhesion flasks. After a week, embryoid bodies were plated on gelatin-coated dishes in DMEM medium supplemented with 20% (vol/vol) fetal bovine serum, 2 mM L-glutamine and 1% (wt/vol) nonessential amino acids. The medium was replaced with fresh medium every 3 days.

### Cell lysis and quantification

All 33 cell lines were cultured until 80% confluence in p10 cell culture dishes in eight replicates. After 48 h of culture without media change, the cells were serum starved for 4 h. Half of the dishes were serum stimulated for 10 min with the addition of 10% fetal bovine serum (FBS), while the remaining dishes remained unstimulated. The stimulation protocols were adapted for cell lines sensitive to starvation or grown without FBS. Starvation of MOLM-13, MV-4-11 and OCI-M1 was performed for 2 h in medium containing 0.01% BSA. SU-DHL-4 cells were not starved, and stimulation was carried out with the addition of an additional 10% of FBS. iPS cells were cultured in serum-free E8 media, and starvation was carried out using an E8 medium without the E8 supplements (containing growth factors) for 4 h. iPS cells were stimulated for 10 min by changing the media back to the E8 medium containing E8 supplements. In addition to the serum-stimulated DLD-1 cells, 12 other p10 dishes of DLD-1 were treated with PV for 15 min in quadruplicates using either dimethyl sulfoxide (DMSO) or 0.5 mM or 1 mM of PV. A 100-mM PV solution was prepared by adding in equal volumes 100 mM sodium orthovanadate and 100 mM 30% H_2_O_2_ in 20 mM HEPES at pH 7.5 (ref. ^21^). All cells were washed twice with ice-cold PBS containing phosphatase inhibitors (2 mM sodium orthovanadate, 1 mM beta-glycerophosphate and 1 mM sodium fluoride). The cells were lysed with the addition of 200 μl boiling lysis buffer (5% SDS, 100 mM Tris pH 8.5, 5 mM tris(2-carboxyethyl)phosphine hydrochloride (TCEP) and 10 mM chloroacetamide (CAA)). The cells were scraped off the plate, and the resulting lysates were heated for 10 min at 95 °C and sonicated using a probe sonicator (Sonic dismembrator, Fisherbrand) with two pulses of 5 s at 40% amplitude. Protein concentrations were determined by bicinchoninic acid (BCA) method (Pierce).

### Digestion and desalting

A total of 600 μg protein per sample was digested overnight using protein aggregation capture (PAC)-based digestion^78^, implemented on a KingFisher Flex platform^79^. Digestion was carried out on hydroxyl beads (ReSyn Bioscience) at a protein-to-beads ratio of 1:2, with endoproteinase LysC (FUJIFILM Wako Pure Chemical Corporation) and trypsin (Sigma-Aldrich) in an enzyme-to-protein ratio of 1:500 and 1:250, respectively. Samples were then acidified to a final concentration of 1% formic acid (FA). The equivalent of 750 ng peptides was aliquoted for proteome analysis. The resulting peptides were desalted on C_18_ Sep-Pak 96-well plates (40 mg sorbent, Waters).

### Phosphopeptide enrichment

Phosphopeptide enrichment was carried out on a KingFisher Flex robot^79,80^ using Ti-IMAC HP magnetic beads (ReSyn Bioscience) at a peptide-to-beads ratio of 1:2. C_18_-bound peptides were directly eluted from the Sep-pak to the KingFisher plate using 75 μl 80% acetonitrile (ACN)^81^ and completed with 200 μl concentrated loading buffer (80% ACN, 8% trifluoroacetic acid (TFA), 0.6 M glycolic acid). In brief, the magnetic beads were added to 500 μl ACN in plate 2; washed with 500 μl loading buffer (80% ACN, 5% TFA, 0.1 M glycolic acid) in plate 3; added to the peptide samples in plate 4; washed in plate 5 (500 μl loading buffer), plate 6 (500 μl washing buffer 2: 80% ACN, 5% TFA) and plate 7 (500 μl washing buffer 3: 10% ACN, 0.2% TFA); and eluted in 200 μl 1% ammonia in plate 8. The enriched phosphopeptides were acidified with 40 μl 10% TFA and loaded on Evotips, according to the manufacturer’s protocol for subsequent LC–MS/MS analysis.

### EGF stimulation and low-input analysis

HeLa cells for EGF stimulation were cultured until 70% confluence and serum starved overnight. Cells from two conditions—untreated control and stimulated with 20 nM EGF (Peprotech) for 8 min—were washed twice with ice-cold PBS and lysed and quantified as described above. Proteins from each condition were bulk digested using PAC, and the peptide concentration for each condition was determined using a nanodrop spectrophotometer (NanoDrop 2000C, Thermo Fisher Scientific) at 280 nm. In total, 1 μg, 5 μg, 10 μg to 20 μg peptides were enriched for phosphopeptides as described above in five replicates for each condition. The enriched phosphopeptides were acidified with 10% FA and loaded on Evotips, according to the manufacturer´s protocol for subsequent LC–MS/MS analysis.

### LC–MS/MS

All samples were analyzed on the Evosep One system (Evosep Biosystems, Denmark) coupled to an Orbitrap Astral mass spectrometer (Thermo Fisher Scientific, Germany) either using the standard or whisper zoom Evosep gradients.

- Standard gradients (30 and 100 SPD) were used with commercial Evosep columns (30 SPD with EV1137: 15 cm × 150 μm, 1.5 μm; 100 SPD with EV1109: 8 cm × 150 μm, 1.5 μm) connected to a stainless steel emitter (30 μm inner diameter; Evosep, EV1086), with temperature set to 40 °C through an externally controlled butterfly heater (MSWil, Cat#PST-ES-BPH-20). The columns were interfaced to the mass spectrometer using an EASY-Spray source
- Whisper zoom gradients (80 and 120 SPD) were used with Aurora Rapid columns (5 cm × 75 μm, 1.7 μm) interfaced to the mass spectrometer using a Nanospray Flex source, with column temperature maintained at 50 °C

The Orbitrap Astral mass spectrometer was operated in positive mode, with a spray voltage set at 2.0 kV (1.8 kV for whisper zoom gradients), funnel radio-frequency level at 40 and heated capillary temperature at 275 °C. All files were acquired using nDIA^19^ with a full scan resolution of 240,000 with a normalized automatic gain control (AGC) target of 500%.

For proteome analysis, the Orbitrap operated across a mass-to-charge ratio range from 380 to 980 *m*/*z* with a maximum injection time set at 3 ms, while it operated over a range from 480 to 1,080 *m*/*z* with a maximum injection time set at 30 ms for phosphoproteomics analysis. Tandem mass spectra were generated across the respective mass range set for MS1 analysis with a scan range from 150 to 2,000 *m*/*z*. Peptide fragmentation was carried out using higher-energy collisional dissociation with a normalized collision energy set at 25%. The normalized AGC target was set at 500%. Different methods with specific isolation window sizes and maximum injection times were used on the basis of the chromatographic gradient used. In total, 30 SPD were used with 2-Th isolation windows and 2.5-ms maximum injection time; 100 SPD were used with 4-Th isolation windows and 2.5-ms maximum injection time, 80 SPD were used with 4-Th isolation windows and 6-ms maximum injection time and 120 SPD were used with 4-Th isolation windows and either 3-ms or 6-ms maximum injection time.

### Data searching

#### Spectronaut

All raw files were searched in Spectronaut version 19 (Biognosys) against the human protein reference database from Uniprot (2022, 20,598 entries) supplemented with a contaminant database from Spectronaut (246 entries). Each cell line was searched separately using directDIA, and the resulting SNEs were combined using the ‘combine SNE’ function. Libraries were generated from the search archives, therefore using the parameters set in the Pulsar search.

Cysteine carbamidomethylation was set as a fixed modification, and N-terminal acetylation and methionine oxidation were set as variable modifications, with a maximum number of variable modifications kept at five, and a maximum number of missed cleavages kept at two. For phosphoproteomics, serine, threonine and tyrosine phosphorylation were added as variable modifications. Cross-run normalization was allowed, with a modification filter on the phosphoproteomics files, where only phosphorylated peptides were used for normalization. The maximum and minimum number of fragments per peptide were set to 25 and 6, respectively. Fragment ion selection was set to evidence based.

For phosphoproteomics analysis, the PTM localization filter was checked, with a threshold of 0.75.

Library-based searches were performed using the same parameters as directDIA.

#### DIA-NN

All raw files were searched on DIA-NN 2-1. An in silico phosphopeptide spectral library was generated using DIA-NN with the human reference database from Uniprot (2022, 20,598 entries) supplemented with a contaminant database from Spectronaut (246 entries). Two missed cleavages were allowed with a maximum of three variable modifications per precursor, including N-terminal methionine excision; cysteine carbamidomethylation and serine; threonine and tyrosine phosphorylation. The peptide length range and precursor charge range were kept to default, while the precursor and fragment mass ranges were adapted to the MS method parameters: 480–1,080 *m*/*z* for the precursors and 150–2,000 *m*/*z* for the fragments.

Empirical spectral library-based and in silico spectral library-based searches were carried out in DIA-NN using the abovementioned parameters. Proteoform scoring was used for phosphoproteomics data.

Fragmentation spectra from the experimental library generated with Spectronaut were compared with the fragmentation profiles obtained from the in silico predicted spectral library using DIA-NN. The cosine correlation spectral angle was measured per phosphoprecursor between the intensities of equivalent fragments. Cosine correlation was calculated using the ‘matchms’^82,83^ library in python.

### Data analysis

#### Data processing

The phosphosite table was exported from Spectronaut, using Spectronaut´s default filtering parameters, filtered for localization probability >0.75 and entries without gene information were filtered out. Cell lines were split into different tables and transformed into PhosphoExperiment (ppe) objects using the PhosR package^84^. Each cell line was filtered for valid values, allowing to conserve phosphosites quantified in three out of four replicates in at least one condition (Ctrl and/or Stim) before proceeding with two-step imputation: (1) site- and condition-specific imputation (scImpute), imputing sites with ≥50% valid values in one condition, sampled from the empirical normal distribution from the same condition, followed by (2) tail-based imputation (ptImpute), imputing remaining missing values with ones from the lower end of the phosphosite intensity normal distribution. All cell lines were joined into a unique table and normalized through mean centering. FCs were calculated per site and per cell line against a background composed of the mean intensity across all cell lines. If a site was unique to a cell line, a positive FC of 1.5 was applied.

Proteome data were analyzed on the protein-group level; each cell line was split into different tables and filtered for valid values, allowing protein groups with at least three valid values out of four in at least one condition. No imputation step was carried out. The tables were joined into a unique table and all intensities were normalized through mean centering. FCs were calculated per protein group per cell line against a background composed of the mean intensity across all cell lines.

#### DIA-NN counts and filtering

For benchmarking DIA-NN and Spectronaut on differentiated stem cells, DIA-NN´s outputs were processed to match Spectronaut´s. Phosphosites were defined with a localization probability ≥0.75 and included multiplicity.

The Parquet report was opened, and entries without gene information were filtered out. The following filters were applied: PG.Q.Value ≤0.05, Global.PG.Q.Value ≤0.01, Lib.PG.Q.Value ≤0.01 (when match between runs was on), Quantity.Quality ≥0.5 and PG.MaxLFQ.Quality ≥0.7 according to the software’s guidelines. The table was further filtered to only keep phosphopeptides. Phosphopeptides were annotated and collapsed per site and per multiplicity before being filtered on the basis of localization probability with a cutoff at 0.75.

#### Stoichiometry calculation

Site stoichiometry was calculated from the raw phosphoproteomics and raw corresponding proteomics datasets (that is, nonfiltered and non-normalized), following the approach of Olsen et al.^16^. Precursor intensities were first collapsed to obtain peptide-sequence-level intensities. Phosphopeptide precursor intensities were then grouped by peptide sequence and by the position of the phosphorylated residue within the protein sequence. Phosphoproteomics and proteomics tables were merged on the basis of protein groups and peptide sequence, resulting in protein-, peptide- and site-sequence-level intensities required for stoichiometry estimation.

Data from the serum-stimulated and control conditions were preprocessed separately. Phosphorylation-site sequences with fewer than two valid values per cell line were removed in each dataset independently. For each phosphorylation-site sequence, the median intensity and standard deviation were computed in both conditions, and the two tables were subsequently merged. Entries lacking median intensities in either the phosphoproteome or proteome were discarded. To retain only sites exhibiting measurable changes, we kept rows where the median intensity in the stimulated condition fell outside the interval defined by the control median ±3× the control standard deviation.

Three ratios (*x*, *y* and *z*) were calculated as the non-log-transformed stimulated/control intensity ratios at the site/peptide-sequence, peptide-sequence and protein levels, respectively. As no global protein abundance changes were expected, entries with protein-level ratios outside the range 0.8 < *z* < 1.2 were removed. The following proportions of phosphopeptide to nonphosphopeptide were calculated:

$$\mathrm{Proportion}\,\mathrm{in}\,\mathrm{control}:A=\frac{z-y}{x-z},$$

$$\mathrm{Proportion}\,\mathrm{in}\,\mathrm{stimulation}:\,B=\frac{x(z-y)}{y(x-z)}.$$

Stoichiometries were then calculated as *A*/(*A* + 1) or *B*/(*B* + 1), depending on the condition. Further filtering of the data was required. When no regulation occurred, *x* and *y* ratios were nearly identical (that is, abs(*x* − *y*) <0.01), which produced extreme stoichiometry values. These cases were labeled as ‘not changing’ and removed. In addition, A and B must be positive to yield valid stoichiometries, as this assumes that the total amount of a given peptide (phosphorylated plus nonmodified) remains constant between conditions. Entries where this assumption was not respected were labeled as ‘breaking the assumption’ and excluded.

Site intensity-based statistical analysis was performed per cell line using differential expression analysis with the limma package between the control and serum stimulated conditions. Sites with Benjamini–Hochberg-corrected *P* values below 1% were considered statistically significantly regulated.

#### Kinase score calculation

##### Kinase activity scores

Unfiltered in vitro kinase reaction relationships were downloaded^29,56^ and reprocessed to avoid the first filtering applied in the iKiP-DB. Kinase–substrate relationships were combined with PSP annotation and filtered to exclude phosphosites that were substrates for over 20 kinases. Kinases with fewer than five substrates were filtered out. The modified iKiP-DB contains kinase–substrate relationships for 369 kinases.

Kinase activity inference was carried out using the modified iKiP database with the PTM-SEA algorithm for ssGSEA^28^. The default parameters were used (that is, sample normalization type as rank, weight at 0.75, statistics using ‘area under RES’, the output score coming from the normalized enrichment score, number of permutation set at 1,000 and correlation type being *Z*-score) except for the minimum overlap, which was decreased to 5.

Motif enrichment analysis was carried out using the Kinase Library^54^. Serine/threonine and tyrosine were processed separately for each cell line. For carrying out multiple comparisons, the publicly available code of the Kinase Library was used in Python. Default parameters were used (that is, percentile rank as enrichment metric and an enrichment threshold of 15 for serine/threonine and 8 for tyrosine).

Activation loop sites were identified on the basis of an activation loop database from the Phomics algorithm. In brief, activation loops were identified in kinases present in the human proteome database on the basis of their amino acid sequence. The activation loop domain is defined between ‘DFG’ and ‘APE’ amino acid sequences or homologs. All serine, threonine and tyrosine amino acids in those loops were compiled and complemented with known activation sites from PSP to generate an ‘activation’ database containing activation sites over 327 kinases.

Kinase activity was inferred using Rokai using the combined PSP and Signor kinase–substrate dataset, with *Z*-scores used as a metric.

##### Cscore calculation

A kinase activity score (Cscore) was compiled using the proteome and phosphoproteome datasets. All calculations were performed on log_2_FCs of one cell line against the mean of all cell lines.

The Cscore is composed of two entities combined as a geometric mean. The first term represents the evidence of the presence of the kinase at the proteome and phosphoproteome levels, while the second term represents the evidence of kinase activity on the phosphoproteome level.

The first score describes the kinase intensity in the proteome data. The score originates from the log_2_FC of the intensity of the kinase in a given cell line and the average intensity of that given kinase across all cell lines. The second score represents the intensity of the kinase on the phosphoproteome. The score was computed as an enrichment-type score, where the mean log_2_FCs of all sites on a given kinase were subtracted to the mean log_2_FCs of all sites in the cell line. The difference was then multiplied by the square root of the number of sites quantified in the kinase.

$$\begin{array}{l}\mathrm{Score}=(\mathrm{mean}\,{\mathrm{log}}_{2}\mathrm{FC}\,\mathrm{of}\,\mathrm{sites}\,\mathrm{on}\,\mathrm{kinase}\\-\mathrm{mean}\,{\mathrm{log}}_{2}\mathrm{FC}\,\mathrm{all}\,\mathrm{sites})\times \sqrt{\mathrm{number}\,\mathrm{of}\,\mathrm{sites}\,\mathrm{on}\,\mathrm{kinase}}.\end{array}$$

Two substrate activity scores were computed. The first one takes into account kinase–substrate relationships on the basis of in vitro kinase reactions established through the use of a modified iKiP database and the PTM-SEA algorithm. The resulting score originated from the product of the resulting normalized enrichment score (NES) with the −log_10_FDR. Likewise, the second substrate activity score is based on motif enrichment analysis using the Kinase Library. The resulting score originates from the product of the NES with the −log_10_FDR. Finally, the activation sites score was computed as an enrichment-type score, where the log_2_FC of all activated sites was subtracted with the log_2_FC of all sites and subsequently multiplied by the square root of the number of activation sites quantified.

$$\begin{array}{l}\mathrm{Score}=(\mathrm{mean}\,{\mathrm{log}}_{2}\mathrm{FC}\,\mathrm{of}\,\mathrm{activation}\,\mathrm{sites}\\-\mathrm{mean}\,{\mathrm{log}}_{2}\mathrm{FC}\,\mathrm{all}\,\mathrm{sites})\times \sqrt{\mathrm{number}\,\mathrm{of}\,\mathrm{activation}\,\mathrm{sites}}.\end{array}$$

All five scores underwent sigmoid transformation to scale them from 0 to 1 without compressing the scores.

$$\mathrm{Scaled}\,\mathrm{score}=\frac{1}{1+{{\rm{e}}}^{-\mathrm{Score}}}.$$

Weights were applied dynamically to the five score components on the basis of their redundancy to reduce the contribution of redundant features to the Cscore. A correlation matrix based on pairwise complete observations is generated and a weight was defined on the basis of the sum of the absolute values of the correlation of a given component with all the other components successively and computed as follows:

$$wi=\frac{1}{1+{\sum }_{i\ne j}|\mathrm{cor}(i,j)|}.$$

Each weight was normalized so that their sum equals 1,

$$wi=\frac{wi}{\sum \mathrm{all}\,\mathrm{weights}}.$$

Each score component was weighted by multiplying the component score by its respective weighing factor before combining the score components into the Cscore as shown below.

Finally, all scores were combined as follows:

$$\mathrm{Cscore}=\sqrt{({S}_{\mathrm{prot}}+{S}_{\mathrm{phos}})\times ({S}_{\mathrm{kinase}-\mathrm{substrate}}+{S}_{\mathrm{motif}}+{S}_{\mathrm{activation}})}.$$

The SKEW factor was calulated representing the relative contribution of both sides of the product in the Cscore calculation^53^. In our case, it represents the relative contribution of the presence (*S*_presence_) or the activity (*S*_activity_) of a kinase,

$$\mathrm{SKEW}=\frac{2}{{\rm\pi }}\arctan \left(\frac{{S}_{\mathrm{presence}}}{{S}_{\mathrm{activity}}}\right).$$

Cell line-specific vulnerabilities were identified with the calculation of a *Z*-score for each kinase across all cell lines. Kinase–cell line pairs with a *Z*-score >2 (that is, activity >median + 2 standard deviations) were considered outliers, while kinases with a kinase activity score >0.5 detected in four or fewer cell lines were classified as unique to those lines.

### Reporting summary

Further information on research design is available in the Nature Portfolio Reporting Summary linked to this article.

## Online content

Any methods, additional references, Nature Portfolio reporting summaries, source data, extended data, supplementary information, acknowledgements, peer review information; details of author contributions and competing interests; and statements of data and code availability are available at https://doi.org/10.1038/s41594-026-01877-6.

## Supplementary information

Supplementary Information Supplementary Figs. 1–4 and Information 1–3.

Reporting Summary

Peer Review File

## Source data

Source Data Fig. 1 Statistical source data.

Source Data Fig. 2 Statistical source data.

Source Data Fig. 3 Statistical source data.

Source Data Fig. 4 Statistical source data.

Source Data Fig. 5 Statistical source data.

Source Data Fig. 6 Statistical source data.

Source Data Extended Data Fig. 3 Statistical source data.

Source Data Extended Data Fig. 7 Statistical source data.

Source Data Extended Data Fig. 8 Statistical source data.

Source Data Extended Data Fig. 10 Statistical source data.

## Data Availability

The mass spectrometry proteomics data are available via the ProteomeXchange Consortium at http://proteomecentral.proteomexchange.org via the PRIDE^85^ partner repository with the dataset identifier PXD068583. Experimental spectral libraries, quantitative proteomics and phosphoproteomics tables, site stoichiometries table, the Cscore table, the list of human activation sites and the modified version of the iKiP-DB are available via Zenodo at https://doi.org/10.5281/zenodo.21469258 (ref. ^86^). Data can be visualized through our web interface at https://phosphoproteomicsatlas.github.io/. Tables visualized through the interface are available via Zenodo at https://doi.org/10.5281/zenodo.20037244 (ref. ^87^). Source data are provided with this paper.

## Code availability

Custom code used for data processing, stoichiometry calculation and Cscore computing are available via GitHub at https://doi.org/10.5281/zenodo.22802853.
