# Supplementary material for: A phosphoproteome atlas of human cell lines reveals the landscape of kinase activity
Source: Nat Struct Mol Biol. 2026 Sep 30;33(10):1561–74. doi: 10.1038/s41594-026-01877-6 (PMC13672073; doi:10.1038/s41594-026-01877-6)
Supplement: Supplementary file 1 [file 41594_2026_1877_MOESM1_ESM.pdf]

nature structural & molecular biology

Supplementary information https://doi.org/10.1038/s41594-026-01877-6

A phosphoproteome atlas of human cell lines reveals the landscape of kinase activity

In the format provided by the authors and unedited

Table of Contents

- **Supplementary figures 1 to 4:**

    1. Protein group and phosphosite counts

    2. Intensity distribution between cell-line specific phosphosites and non-cell line specific ones

    3. Overview of the number of kinase-substate relationships in the PSP and modified iKiP-DB databases

    4. Combined kinase score allows to study kinase family enrichments and specific activity changes across functional cell line clusters

- **Supplementary information 1 to 3:**

    1. Cscore calculation principle and R-based code

    2. Evaluation of the Cscore and SKEW on inhibited kinases

    3. Kinase Library Code

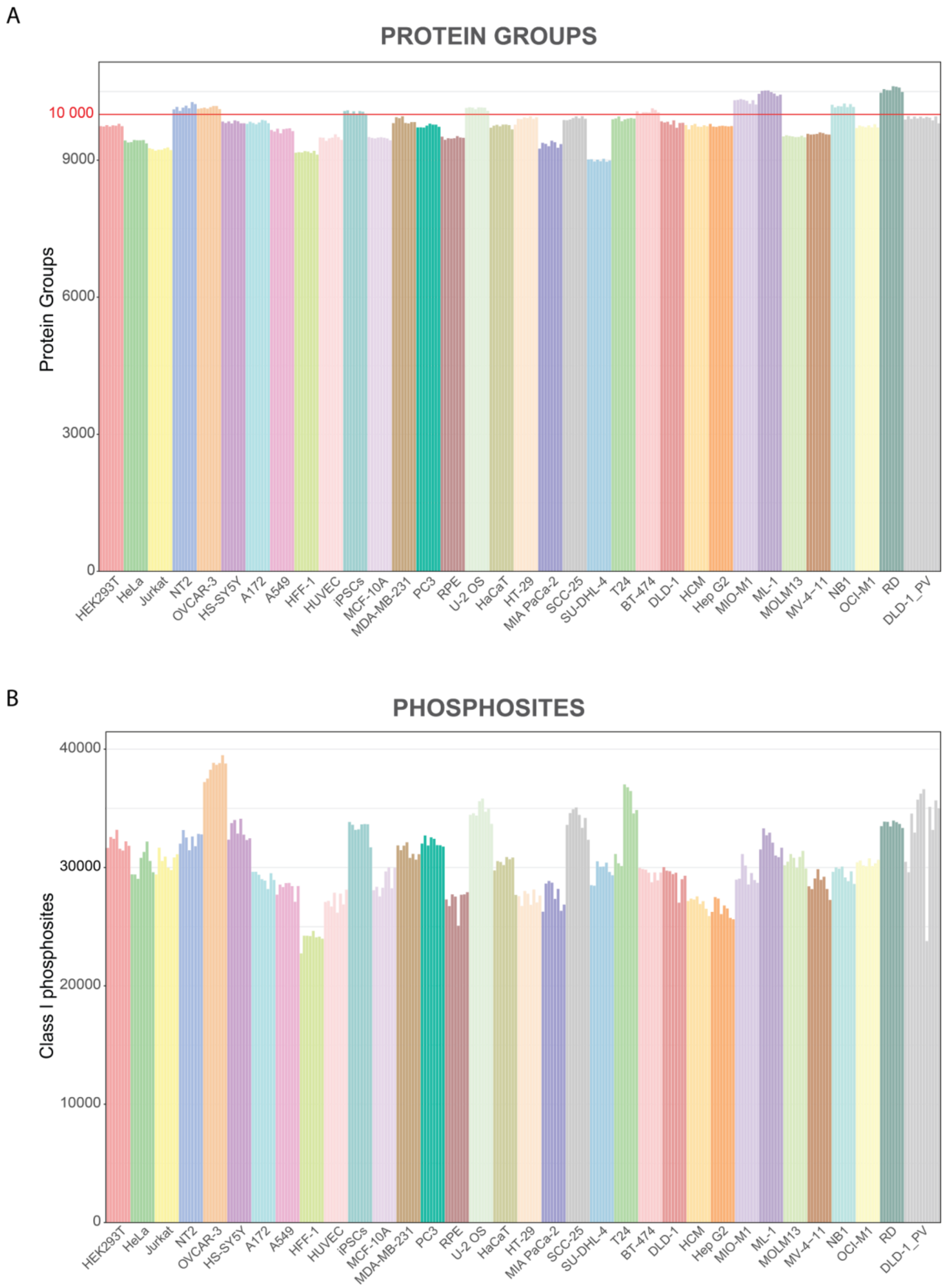

Supplementary figure 1: Protein group and phosphosite counts. A- Protein group count per raw file after filtering for valid values within a cell line (¾ valid values in at least one condition (ie. control or serum-stimulation)). B- Class 1 phosphosite count per raw file after filtering for valid values within a cell line (¾ valid values in at least one condition (ie. control or serum-stimulation)).

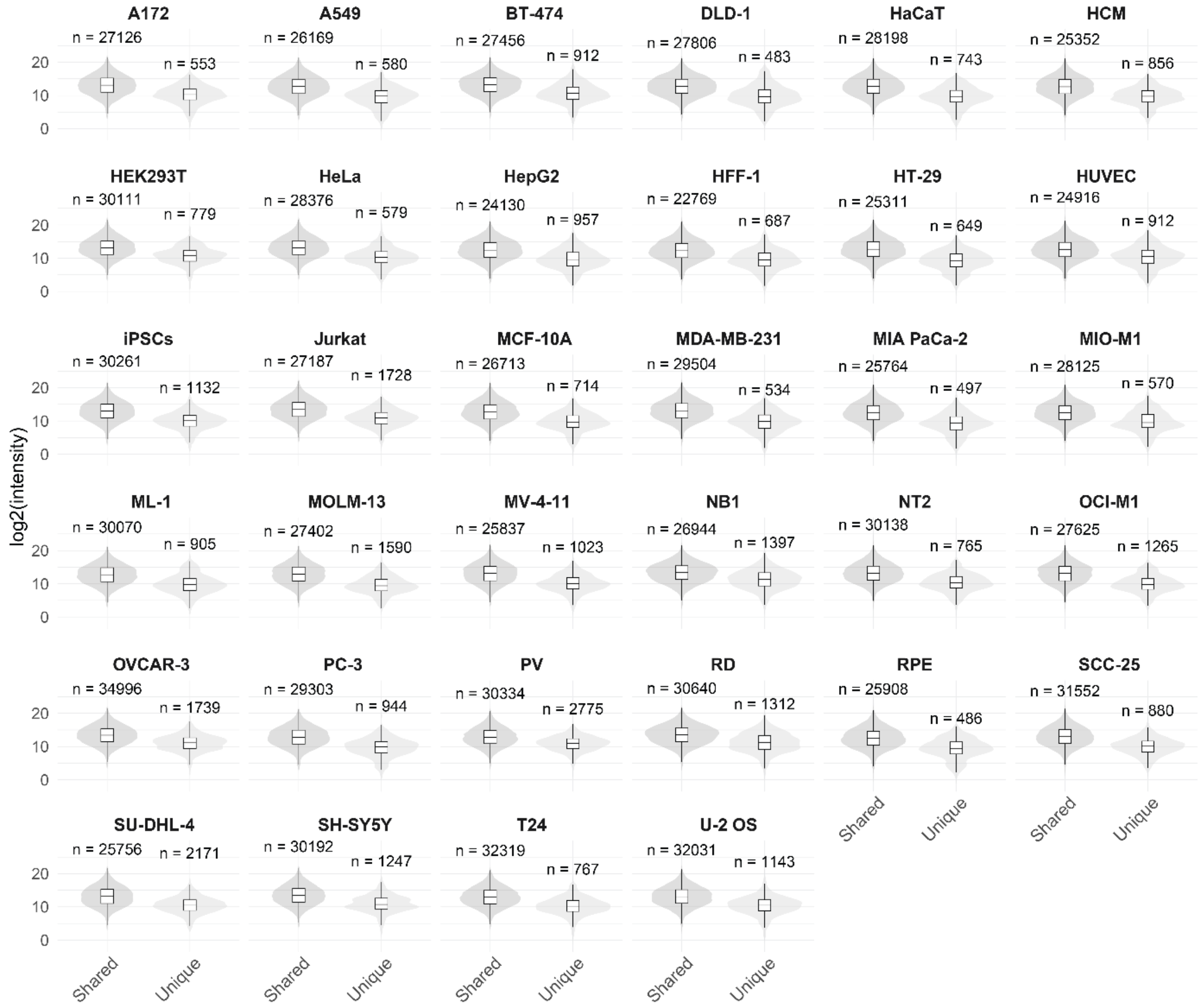

Supplementary figure 2: Intensity distribution between cell-line specific phosphosites and non-cell line specific ones. n indicate the number of phosphosites per boxplots. Box plots show the median (centre line), the first and third quartiles (box limits), and the minimum and maximum values (whiskers).

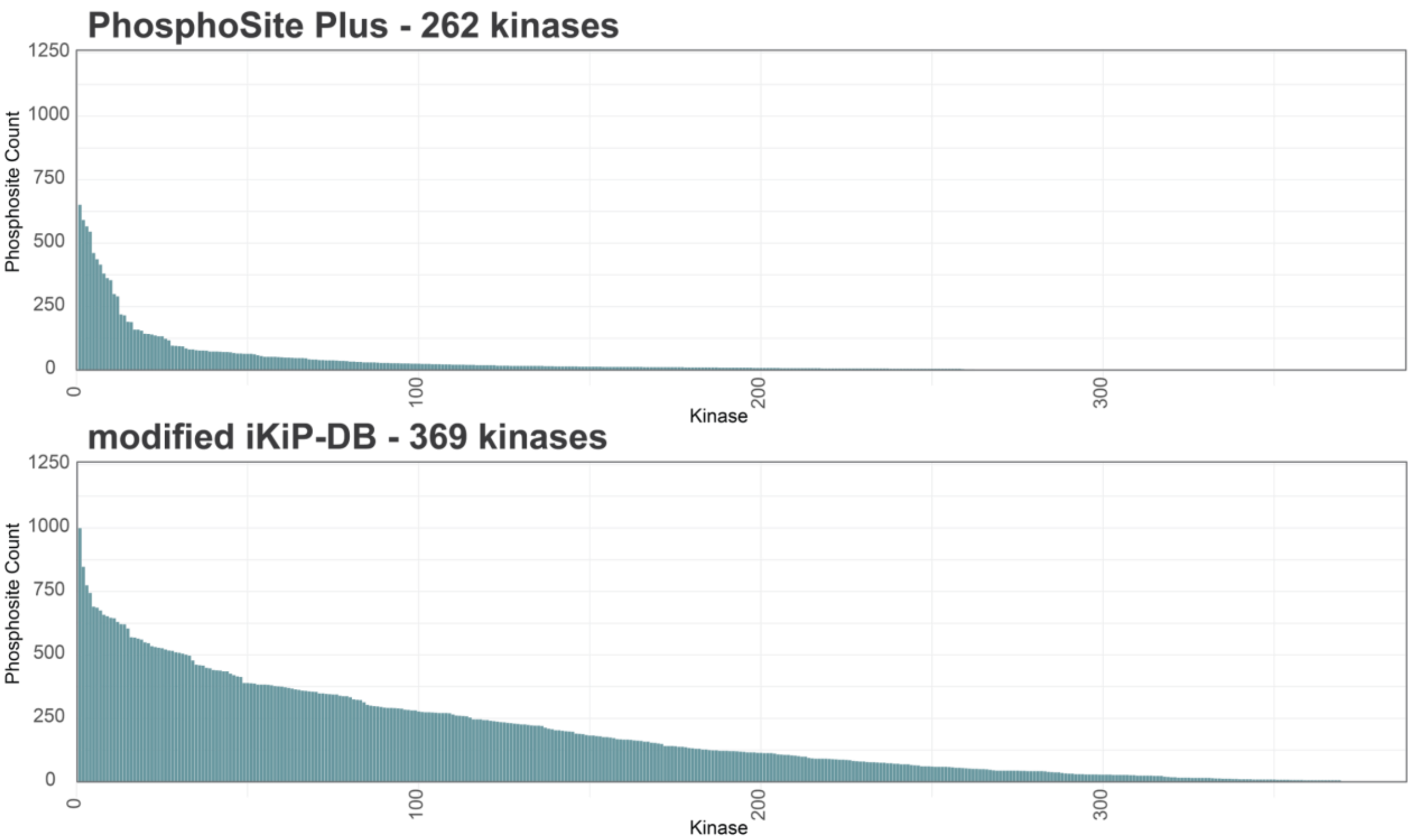

Supplementary figure 3: Overview of the number of kinase-substate relationships in the PSP and modified iKiP-DB databases. Kinases are ranked based on their number of substates in the respective database.

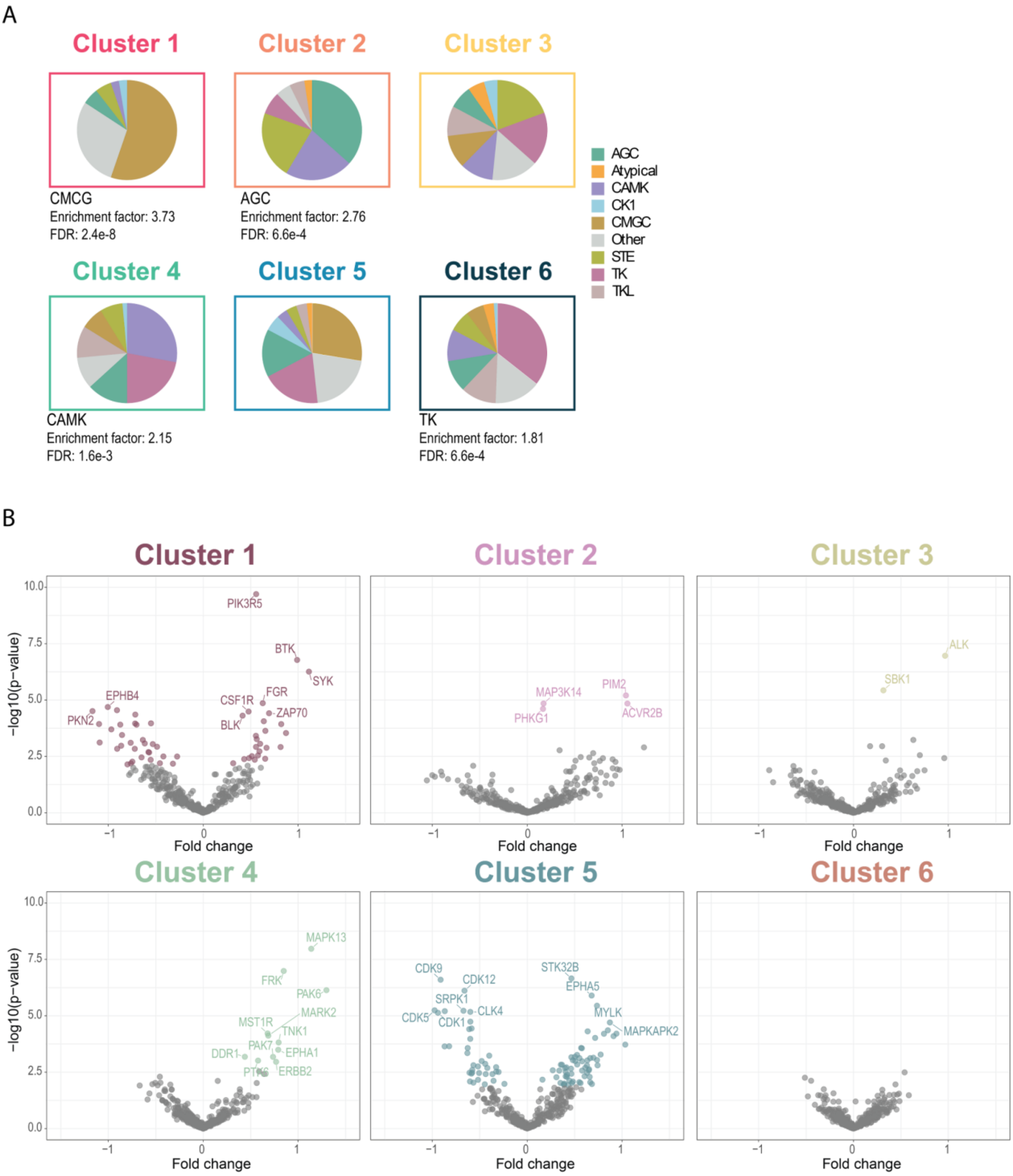

Supplementary figure 4: Combined kinase score allows to study kinase family enrichments and specific activity changes across functional cell line clusters. A- Distribution of the kinase families per cluster, which were defined in Figure 6C. Clustering the kinase activity profiles revealed six groups of kinases with similar activity patterns across the cell lines. Fisher enrichment analysis was carried out for each cluster. Benjamini-Hochberg correction was applied for FDR calculation. The first cluster was dominated by CMGC kinases and showed low activity in non-cancerous and high activity in cancerous cell lines, likely reflecting a basal, non-proliferative signaling state in non-cancerous cells. Another cluster, enriched in AGC kinases, displayed the opposite pattern, with higher activity in non-cancerous cells. CAMK kinases formed a further cluster, characterized by sporadic detection and cell line-specific activity. An additional cluster was enriched in tyrosine kinases, whose activity was highly specific to individual cell lines, reflecting selective expression and signaling. B- Volcano plot for each functional cell line cluster, which were defined in Extended Data Figure 8. Fold changes were calculated per

cluster against the background of all the other cell lines. Significance was assessed using a two-sided Student´s t-test with Benjamini-Hochberg FDR correction, and colored dots represent kinases with an activity statistically significantly changed at a FDR of 5%. Specific kinases with high activity in different functional clusters were identified, with kinases specifically involved in immune regulation (i.e. BTK, SYK, FGR) in the blood cancer cell lines, or PIM2 in the pluripotent cell cluster, which has been associated with cancer therapy resistance.

1. Cscore calculation principle and R-based code

The Cscore combines two complementary dimensions of kinase biology using a geometric-like formulation:

- **Kinase presence** (protein and phosphoprotein levels)
- **Kinase activity** (substrate regulation and motif-based inference)

It is built from five components, each computed independently and then combined into a single score. To reduce redundancy, each component is dynamically weighted based on its correlation with the others.

All calculations are performed on log2 fold changes relative to the mean across cell lines.

Prerequisites

- Kinase Uniprot ↔ gene name mapping table
- Activation site database
- Processed proteomics data
- Processed phosphoproteomics data
- PTM-SEA with modified iKiP-DB
- Kinase Library (Python environment)

Component scores

1. Sprot — kinase abundance (proteome)

- Load proteomics data with log2 fold change values
- Expand protein groups to have one row per Uniprot ID
- Subset to kinases
- Apply sigmoid transformation (scale 0–1):

$$Scaled\ score\ = \frac{1}{1 + e^{-Score}}$$

2. Sphos — kinase phospho-level intensity

- Load phosphoproteomics data with log2 fold change values
- Identify the kinases and process an enrichment type score as follows:

$$Score\ = (mean\ log2\ FC\ of\ sites\ on\ kinase\ -\ mean\ log2\ FC\ all\ sites) * \sqrt{number\ of\ site\ on\ kinase}$$

- Apply sigmoid transformation

3. Skinase-substrate — substrate-based activity (PTM-SEA)

This component captures kinase activity based on experimentally derived kinase–substrate relationships using a modified iKiP database.

- Generate PTM-SEA input tables from the phosphoproteomics data (same input as for *Sphos*). An individual table is generated per cell line.
- **Outside of the script:** Perform PTM-SEA kinase activity analysis using the R-based interface which will loop over all the generated input tables
- Read and combine all PTM-SEA output tables
- Harmonize gene names and map to Uniprot identifiers
- Compute the score as follows:

$$Score = NES * -log10(FDR)$$

- Apply a sigmoid transformation

4. Smotif — motif-based activity (Kinase Library)

This component infers kinase activity from sequence motif enrichment.

- Generate Kinase Library input tables from phosphoproteomics data. One table is generated per cell line.
- **Outside of the script:** Run motif enrichment analysis using the Kinase Library python script (https://github.com/TheKinaseLibrary/kinase-library/tree/master/src/notebooks) using motif enrichment analysis (mea) for multiple comparisons, where the code iterates through all input tables and exports enrichment results per S/T or Y per cell line. Analyses are performed separately for:
    - Serine and threonine kinases (kin_type = "ser_thr", thresh = 15)
    - Tyrosine kinases (kin_type = "tyrosine", thresh = 8)

  Note: The used code of the Kinase Library MEA for multiple comparison is available at the end of the document.

- Read and combine output tables
- Harmonize gene names and map to Uniprot identifiers
- Compute the score as:

$$Score = NES * -log10(FDR)$$

- Apply a sigmoid transformation

5. Sactivation — activation loop phosphorylation

This component captures kinase regulation via phosphorylation of activation loop sites. Activation loops were predicted using Phomics.

- Load activation site database
- Use the phosphoproteomics table (same as for *Sphos*) and identify experimentally quantified activation sites
- Compute an enrichment-like score as follows:

$$Score = (mean\ log2FC\ of\ activation\ sites - mean\ log2\ FC\ all\ sites) * \sqrt{number\ of\ activation\ sites}$$

- Apply a sigmoid transformation

Component weighting

Weights were applied dynamically to the 5 score components to based on their redundancy to reduce their contribution in the Cscore.

- Merge all component scores into a single table and convert it to long format.
- Compute the correlation matrix across components based on pairwise complete observation.
- For each component, the weight is defined using the sum of the absolute values of the correlation of a given component with all the other components successively. The weight is computed as follows:

$$wi = \frac{1}{1 + \sum_{j \neq i} |cor(i,j)|}$$

- Normalize weights so that their sum equals 1:

$$wi = \frac{wi}{\sum all\ weights}$$

- All components are being weighted by multiplying the component score by its respective weighing factor.

Cscore calculation

Each component is multiplied by its corresponding weight and combined as:

$$Cscore = \sqrt{(S_{prot} + S_{phos}) \times (S_{kinase-substrate} + S_{motif} + S_{activation})}$$

This formulation separates kinase presence (left term of the product) from kinase activity (right term of the product).

SKEW calculation

- SKEW factor represents the relative contribution of both sides of the product in the Cscore calculation. In our case it represents the relative contribution of the presence or the activity of a kinase, and is calculated as follows:

$$SKEW = \frac{2}{\pi} arctan\left(\frac{Spresence}{Sactivity}\right)$$

Output

The final table is restructured and can be exported in long or wide format, including:

1. Individual component scores
2. Weighted Cscore
3. SKEW factor

2. Evaluation of the Cscore and SKEW on inhibited kinases

The Cscore provides a combined value of kinase activity, combining the need for a kinase to be both present and active. To validate our ability to distinguish between highly expressed and highly active kinases, we have applied our Cscore and SKEW strategy to a publicly available dataset (PXD038947) (Hybrid-DIA: intelligent data acquisition integrates targeted and discovery proteomics to analyze phospho-signaling in single spheroids - PMID: 37328457) in which HeLa cells were treated with inhibitors of EGFR (Lapatinib), MEK2 (PD0325901), or PI3K (Wormannin), followed by EGF stimulation for 10 and 90 minutes (Figure R18). Kinase inhibition represents the situation the reviewer described, where a kinase is expressed but not active. In that case, we expect upon inhibition the Cscore to decrease and to be shifted towards the lower SKEW values, reflecting a shift towards presence-driven kinases.

As proteome data were unavailable for this experiment, we used our own HeLa proteome and phosphoproteome to assess the presence of given kinases by performing Z-score within our cell line for representing the relative abundance of the kinase, instead of computing fold changes against a background. Cscores were computed by combining data acquired with both targeted and DIA methods. Fold changes were computed between EGF-stimulated conditions with and without inhibitor-treatment at each time point.

The results were consistent with biological expectations (**Panel A**). Since EGFR is upstream of both the PI3K and MEK, EGFR inhibition was expected to inhibit signalling through both axes, which is indeed observed.
We can observe EGFR activity to be reduced and shifted towards lower SKEW upon inhibition both at 10 and 90 minutes. PI3K inhibition reduced the activity of its downstream kinases AKT1, AKT2, and MTOR. This pattern is also visible through upstream EGFR inhibition, while their activity remained unaffected with MEK inhibition (except for MTOR at 10 min). Of note, AKT2 remained inhibited longer than AKT1.

MEK inhibition did not produce a detectable Cscore change for MEK2 or ERK1 directly. However, reduced activity of the ERK downstream substrate RPS6KA1 was observed under both MEK and EGFR inhibition.

We followed up on the lack of inhibition observed for MEK2 and ERK1. Threonine and tyrosine activation sites on ERK1 following the TEY motif (T202 and Y204) were quantified from the targeted data. Fold changes per experiment are displayed in **panel B**. While the tyrosine phosphorylation was being strongly inhibited upon MEKi, and at 90 minutes with EGFRi (consistent with MEK being downstream of EGFR), the threonine showed higher phosphorylation levels. Since ERK1 activation requires dual phosphorylation, ERK1 is functionally inactive under MEK2 inhibition in our dataset, yet kinase activity inference methods used for the Cscore calculation (PTM-SEA, Kinase Library) reported elevated MEK2 activity, with elevated FDR, likely due to conflicting phosphorylation signals on the substrates (**Panel C**). This example highlights the limitations of the mathematical models applied to kinase activity scoring in complex biological contexts, which consequently affect the Cscore

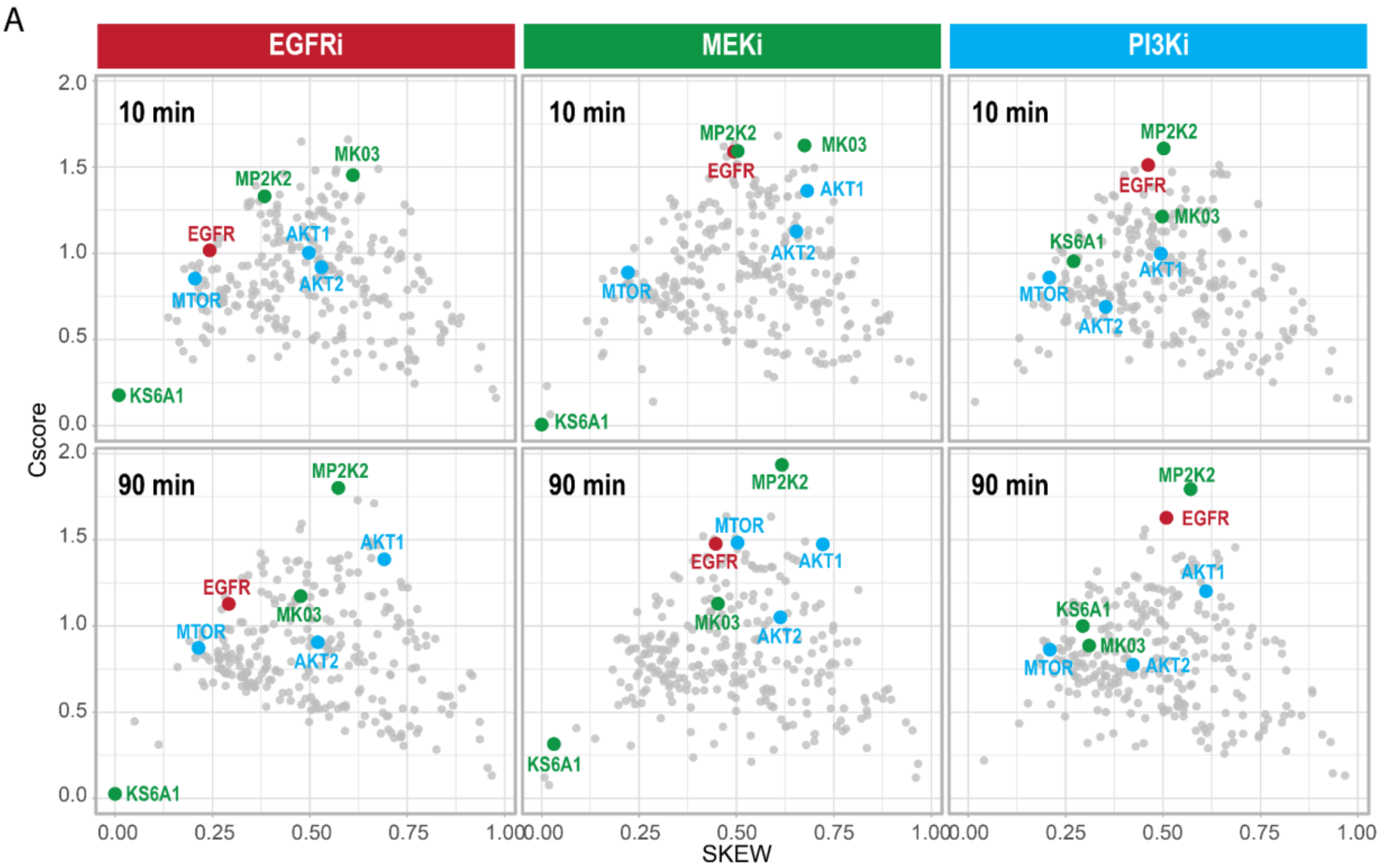

B MK03 (ERK1) log2 fold change site intensity

	EGFRi 10min	EGFRi 90min	MEKi 10min	MEKi 90min	PI3Ki 10min	PI3Ki 90min
MK03_T202	0.60	0.93	1.58	-0.56	-0.05	-1.10
MK03_Y204	-0.40	-1.70	-1.60	-1.18	-0.31	-0.66

C MP2K2 (MEK2) kinase activity

	PTM-SEA		Kinase Library	
	NES	FDR	NES	FDR
EGFRi - 10min	-1.22	0.467	1.13	0.366
EGFRi - 90min	-2.32	0.128	0.97	0.912
MEKi - 10min	1.60	0.395	1.34	0.089
MEKi - 90min	-5.71	0.009	1.22	0.371
PI3Ki - 10min	-1.77	0.305	0.91	0.841
PI3Ki - 90min	-4.30	0.011	-0.88	0.994

Ability of the Cscore to identify expressed and inactive kinases. A- Cscore and SKEW per kinase upon three kinase inhibitors at 10 minutes or 90 minutes after EGF stimulation. B- ERK1 (MK03) log2 fold changes of phosphosites requiring dual phosphorylation for activation. C- MEK2 (MP2K2) kinase activity scores and FDR using PTM-SEA with the modified iKiP-DB or the Kinase library. NES- Normalised enrichment score. FDR- False discovery rate.

3. Kinase Library Code

The MEA for multiple comparison code from the Kinase Library (https://github.com/TheKinaseLibrary/kinase-library/tree/master/src/notebooks) was adapted to the studied dataset. The code below is for serine and threonine motif enrichment analysis. For tyrosine motif enrichment analysis the following changes need to be made: kin_type = "tyrosine", thresh = 8, output_dir = './resultsY'.

Perform MEA:

```
%matplotlib inline
plt.rcParams['figure.figsize'] = [10, 5] #For Jupyter Notebook
file_list = sorted([name for name in os.listdir('./test_files/CellLines/') if not name.startswith('.')])
conds_name = ['A172', 'A549', 'BT474', 'DLD1', 'HaCat', 'HCM', 'HEK293T', 'HeLa', 'HepG2', 'hff',
'HT29', 'HUVEC', 'iPSCs', 'Jurkat', 'MCF10A', 'MDAMB235', 'MiaPaca', 'MioM1', 'ML1', 'Molm13',
'MV411', 'NB1', 'NT2', 'OCIM1', 'OVCAR3', 'RD', 'RPE', 'SUDHL4',  'SY5Y', 'T24', 'U2OS']
kin_type = 'ser_thr'
method = 'percentile_rank'
thresh = 15
rank_col = 'logFC'
motif_enrichment_results = {}
for i,cond_file in enumerate(file_list):
    cond_name = conds_name[i]
    print(f"{i}: {cond_name}")
    phospho_data = pd.read_csv(f'./test_files/CellLines/{cond_file}', sep='\t')
    phospho_data['logFC'] = -phospho_data['logFC']
    pps_data = kl.PhosphoProteomics(phospho_data, seq_col='site sequence')
    ser_thr_percentiles = pps_data.percentile(kin_type, values_only=True)
   ranked_phos = kl.RankedPhosData(dp_data=phospho_data, rank_col=rank_col, seq_col='site
sequence')
    ranked_phos.submit_percentiles(kin_type, ser_thr_percentiles, suppress_messages=True)
    mea_res = ranked_phos.mea(kin_type=kin_type, kl_method=method, kl_thresh=thresh)
    motif_enrichment_results[cond_name] = mea_res
```

Export files:

```
output_dir = './resultsST'
os.makedirs(output_dir, exist_ok=True)
for cond_name, enr_results in motif_enrichment_results.items():
    df = enr_results.enrichment_results.reset_index()
    filename = os.path.join(output_dir, f'{cond_name}_enrichment_results.csv')
    df.to_csv(filename, index=False)
```

!!! The same analysis needs to be carried out for phosphoryrosine where kin_type = 'tyrosine' and thresh = 8
