# Supplementary material for: A phosphoproteome atlas of human cell lines reveals the landscape of kinase activity
Source: Nat Struct Mol Biol. 2026 Sep 30;33(10):1561–74. doi: 10.1038/s41594-026-01877-6 (PMC13672073; doi:10.1038/s41594-026-01877-6)
Supplement: Supplementary file 2 [file 41594_2026_1877_MOESM2_ESM.pdf]

nature portfolio

Corresponding author(s): Jesper V.Olsen

Last updated by author(s): 23/07/2026

Reporting Summary

Nature Portfolio wishes to improve the reproducibility of the work that we publish. This form provides structure for consistency and transparency in reporting. For further information on Nature Portfolio policies, see our Editorial Policies and the Editorial Policy Checklist.

Statistics

For all statistical analyses, confirm that the following items are present in the figure legend, table legend, main text, or Methods section.

n/a	Confirmed	
☐	☒	The exact sample size (*n*) for each experimental group/condition, given as a discrete number and unit of measurement
☐	☒	A statement on whether measurements were taken from distinct samples or whether the same sample was measured repeatedly
☐	☒	The statistical test(s) used AND whether they are one- or two-sided<br>*Only common tests should be described solely by name; describe more complex techniques in the Methods section.*
☒	☐	A description of all covariates tested
☐	☒	A description of any assumptions or corrections, such as tests of normality and adjustment for multiple comparisons
☐	☒	A full description of the statistical parameters including central tendency (e.g. means) or other basic estimates (e.g. regression coefficient) AND variation (e.g. standard deviation) or associated estimates of uncertainty (e.g. confidence intervals)
☐	☒	For null hypothesis testing, the test statistic (e.g. *F*, *t*, *r*) with confidence intervals, effect sizes, degrees of freedom and *P* value noted<br>*Give P values as exact values whenever suitable.*
☒	☐	For Bayesian analysis, information on the choice of priors and Markov chain Monte Carlo settings
☐	☒	For hierarchical and complex designs, identification of the appropriate level for tests and full reporting of outcomes
☐	☒	Estimates of effect sizes (e.g. Cohen's *d*, Pearson's *r*), indicating how they were calculated

Our web collection on statistics for biologists contains articles on many of the points above.

Software and code

Policy information about availability of computer code

Data collection	Spectronaut (v19), DIA-NN (2-1)
Data analysis	R (4.5.2), Rokai (2.3.0), PTM-SEA, KinaseLibrary, PhosR (1.13.1), ConsensusIClusterPlus (1.74.0), String (12.0), Cytoscape (I3.10.4), ceLogo (1.3.8), KinMap.

For manuscripts utilizing custom algorithms or software that are central to the research but not yet described in published literature, software must be made available to editors and reviewers. We strongly encourage code deposition in a community repository (e.g. GitHub). See the Nature Portfolio guidelines for submitting code & software for further information.

Data

Policy information about availability of data

All manuscripts must include a data availability statement. This statement should provide the following information, where applicable:

- Accession codes, unique identifiers, or web links for publicly available datasets
- A description of any restrictions on data availability
- For clinical datasets or third party data, please ensure that the statement adheres to our policy

The mass spectrometry proteomics data have been deposited to the ProteomeXchange Consortium (http://proteomecentral.proteomexchange.org) via the PRIDE 86 partner repository with the data set identifier PXD068583.
Experimental spectral libraries, quantitative proteomics and phosphoproteomics tables, site stoichiometries table, Cscore table, list of human activation sites and

the modified version of the iKiP-DB are available on Zenodo (10.5281/zenodo.21469258). Source data are provided with this paper. Data can be visualised through our web interface: https://phosphoproteomicsatlas.github.io/. Tables visualised through the interface are hosted on Zenodo (10.5281/zenodo.20037244).

Research involving human participants, their data, or biological material

Policy information about studies with human participants or human data. See also policy information about sex, gender (identity/presentation), and sexual orientation and race, ethnicity and racism.

Reporting on sex and gender	NA
Reporting on race, ethnicity, or other socially relevant groupings	NA
Population characteristics	NA
Recruitment	NA
Ethics oversight	NA

Note that full information on the approval of the study protocol must also be provided in the manuscript.

Field-specific reporting

Please select the one below that is the best fit for your research. If you are not sure, read the appropriate sections before making your selection.

☒ Life sciences ☐ Behavioural & social sciences ☐ Ecological, evolutionary & environmental sciences

For a reference copy of the document with all sections, see nature.com/documents/nr-reporting-summary-flat.pdf

Life sciences study design

All studies must disclose on these points even when the disclosure is negative.

Sample size	33 human cell lines, and one cell line replicated with pervanadate treatment
Data exclusions	SCC-25 and PC-3 were excluded from downstream analysis due to mycoplasma contamination. DLD-1 treated cells with pervanadate were excluded from downstream analysis to not bias phosphotyrosine quantification of the other cell lines.
Replication	Replicate numbers were chosen to assess reproducibility and data completeness: four biological replicates per condition for the cell-line atlas, six technical replicates for gradient benchmarking, and five replicates per input amount and condition for the low-input experiment.
Randomization	NA
Blinding	Blinding was not considered necessary. Group identity was defined by objective conditions: cell line and stimulation.

Reporting for specific materials, systems and methods

We require information from authors about some types of materials, experimental systems and methods used in many studies. Here, indicate whether each material, system or method listed is relevant to your study. If you are not sure if a list item applies to your research, read the appropriate section before selecting a response.

Materials & experimental systems

n/a	Involved in the study	
☒	☐	Antibodies
☐	☒	Eukaryotic cell lines
☒	☐	Palaeontology and archaeology
☒	☐	Animals and other organisms
☒	☐	Clinical data
☒	☐	Dual use research of concern
☒	☐	Plants

Methods

n/a	Involved in the study	
☒	☐	ChIP-seq
☒	☐	Flow cytometry
☒	☐	MRI-based neuroimaging

Eukaryotic cell lines

Policy information about cell lines and Sex and Gender in Research

Cell line source(s)	33 human cell lines were used in this study: A172 (CRL-1620), A549 (CCL-185), DLD-1 (CCL-221), HaCaT (CVCL_0038), HEK293T (CRL-3216), HeLa (CRL-2), Hep G2 (HB-8065), HFF-1 (SCRC-1041), HT-29 (HTB-38), MDA-MB-231 (HTB-26), MIA PaCa-2 (CRL-1420), MIO-M1 (CVCL_0433), ML-1 (ACC 464), NT2 (CRL-1973), RD (CCL-136), RPE (CRL-4002), SH-SY5Y (CRL-2266), T24 (HTB-4), U-2 OS (HTB-96), BT-474 (HTB-20), Jurkat (TIB-152), MOLM-13 (ACC 554), NB1 (CVCL_1440), OVCAR-3 (HTB-161), SCC-25 (CRL-1628), PC3 (CRL-1435), MV-4-11 (CRL-9591), OCI-M1 (ACC 529), SU-DHL-4 (CRL-2957), MCF-10A (CRL-10317), HUVEC, HCM (C-12810) and Human iPSCs hi12 (CRL-2429).
Authentication	All cell lines were obtained from authenticated commercial or academic sources.
Mycoplasma contamination	All cell lines were tested for mycoplasma post data acquisition using the generated proteomics data. SCC-25 and PC-3 tested positive.
Commonly misidentified lines (See ICLAC register)	No commonly misidentified cell line was used in this study.

Plants

Seed stocks	*Report on the source of all seed stocks or other plant material used. If applicable, state the seed stock centre and catalogue number. If plant specimens were collected from the field, describe the collection location, date and sampling procedures.*
Novel plant genotypes	*Describe the methods by which all novel plant genotypes were produced. This includes those generated by transgenic approaches, gene editing, chemical/radiation-based mutagenesis and hybridization. For transgenic lines, describe the transformation method, the number of independent lines analyzed and the generation upon which experiments were performed. For gene-edited lines, describe the editor used, the endogenous sequence targeted for editing, the targeting guide RNA sequence (if applicable) and how the editor was applied.*
Authentication	*Describe any authentication procedures for each seed stock used or novel genotype generated. Describe any experiments used to assess the effect of a mutation and, where applicable, how potential secondary effects (e.g. second site T-DNA insertions, mosiacism, off-target gene editing) were examined.*
