# Supplementary material for: A phosphoproteome atlas of human cell lines reveals the landscape of kinase activity
Source: Nat Struct Mol Biol. 2026 Sep 30;33(10):1561–74. doi: 10.1038/s41594-026-01877-6 (PMC13672073; doi:10.1038/s41594-026-01877-6)
Supplement: Supplementary file 3 [file 41594_2026_1877_MOESM3_ESM.pdf]

nature portfolio

Peer Review File

A phosphoproteome atlas of human cell-lines reveals the landscape of kinase activity

Corresponding Author: Professor Jesper Olsen

Version 0:

Decision Letter:

6th Feb 2026

Dear Professor Olsen,

Thank you again for submitting your manuscript "The Human Cell Line Phosphoproteome Atlas: A Deep Empirical Resource Revealing Kinase Activity Landscapes". I apologise for the delay in responding, which resulted from the difficulty in obtaining suitable referee reports. Nevertheless, we now have comments (below) from the 3 reviewers who evaluated your paper. In light of these reports, we remain interested in your study and would like to see your response to the comments of the referees in the form of a revised manuscript.

You will see that while all referees appreciate the potential resource value of the study, they raise crucial concerns with respect to technical aspects and request both further functional validation of the Cscore and the potential biological insight imparted by the study. In particular, both Reviewer #1 and Reviewer #3 voice very important concerns about whether FDRs are affected by the merging of data by different cell-lines, about the language and conclusions made with respect to the importance of absolute stoichiometry versus relative enrichment, and note several ambiguities, issues, and at cases errors with respect to how the the data have been acquired, analysed and presented. To re-engage with the reviewers in a revised manuscript all these points must be comprehensively and convincingly addressed. Further to addressing all technical points, we would editorially expect that the Cscore would be improved in accordance to the guidance of Reviewer #3 with respect to weighting parameters which are not independent, but currently treated as independent, and the manuscript would appear more focused on only certain technical advances and at least 1-2 biologically relevant outcomes (e.g. convincingly identifying vulnerabilities). To the latter point, Reviewer #3 also recommends that orthogonal validation of the inferred therapeutic vulnerabilities identified is provided. We editorially agree that such validation with specific examples would be valuable and would notably increase the chances of successful peer-review, but we would not expect functional experiments beyond the proposed timeframe of revisions.

Please note that we are reluctant to see manuscripts undergo multiple rounds of revisions, for both the authors' and the reviewers' sake. We therefore ask you to ensure that the revisions are extensive and comprehensively address the points of the reviewers so that we can editorially decide to re-engage with them. If you deem that the referees' requests and the editorial intepretation of their concerns is too demanding, we could facilitate a transfer at Nature Communications or the EMBO Journal, upon consulting with them to gauge their interest and understanding which would be their requests for a potential revision given their different editorial bar.

Our standard extended-revisions period is 4 months. We are committed to providing a fair and constructive peer-review process and encourage you to contact us if you would like to discuss the reviews, anticipate any delay with the resubmission or would like to request additional time for performing revisions. We will be happy to consider your revision as long as nothing similar has been accepted for publication at NSMB or published elsewhere. Should your manuscript be substantially delayed without notifying us in advance and your article is eventually published, the received date would be that of the revised, not the original, version.

REPORTING AND DATA AVAILABILITY: Please provide updated files as follows with your revision. As you already know, we put great emphasis on ensuring that the methods and statistics reported in our papers are correct and accurate.

1. REPORTING SUMMARY: Please provide an updated file:

https://www.nature.com/documents/nr-reporting-summary.pdf

This form is a dynamic ‘smart pdf’ and must be downloaded and completed in Adobe Reader.

2. REPORTING OF STRUCTURAL DATA: If there are additional or modified structures in the revision, please submit the corresponding PDB validation reports with your other files, and use the figshare integration system to provide access to maps, half-maps, and models.

Manuscripts reporting new structures should contain a table summarizing structural and refinement statistics. Please include these tables for new cryo-EM or -ET (https://www.nature.com/documents/nr-tables-cryo-em.doc and modifying this file for ET), NMR (https://www.nature.com/documents/nr-tables-nmr.doc) and X-ray crystallography (https://www.nature.com/documents/nr-tables-xray.doc). To facilitate assessment of the quality of the structural data, a stereo image of a portion of the electron density map (for crystallography papers) or of the superimposed lowest energy structures (>10; for NMR papers) should be provided with the submitted manuscript. If the reported structure represents a novel overall fold, a stereo image of the entire structure (as a backbone trace) should also be provided. For cryo-EM structures, a representative micrograph showing individual particles should be provided in the figures alongside a processing workflow (please see our Editorial for more information: https://www.nature.com/articles/s41594-025-01567-9).

DATA DEPOSITION: We require deposition of coordinates (and, in the case of crystal structures, structure factors) into the Protein Data Bank with the designation of immediate release upon publication (HPUB). Electron microscopy-derived density maps and coordinate data must be deposited in EMDB and released upon publication. Deposition and immediate release of NMR chemical shift assignments are requested. To avoid delays in publication, dataset accession numbers must be supplied with the revised manuscript and appropriate release dates must be indicated at the galley proof stage.

3. REPORTING OF LIGHT MICROSCOPY DATA: For any revision that includes light microscopy data, we ask our authors to please include a completed light microscopy reporting table [https://www.nature.com/documents/Light_microscopy_reporting_table.xlsx] to ensure the methods are described thoroughly. The table will be available to reviewers and ultimately published, should the manuscript be accepted at the journal. When submitting the revised version of your manuscript, please pay close attention to our href="https://www.nature.com/nature-portfolio/editorial-policies/image-integrity">Digital Image Integrity Guidelines.

4. CODE AND COMPUTATIONAL WORK: If newly developed, unpublished code was used in this work, it must be provided with the revision and access to it must be disclosed in the Code Availability Statement in the manuscript. Please also provide the completed software submission checklist. This form is a dynamic ‘smart pdf’ and must be downloaded and completed in Adobe Reader, instead of opening it in a web browser: https://www.nature.com/documents/nr-software-policy.pdf

If molecular dynamics (MD) simulations were performed, please refer to this Editorial and provide a completed MD simulations checklist with your revision: https://www.nature.com/articles/s42003-023-04653-0

Lastly, if it applies, please complete and upload the completed machine learning checklist with your submission for re-review: https://www.nature.com/documents/machine-learning-checklist.pdf

5. SOURCE GEL IMAGES: Unprocessed scans should be provided for all gels and western blots presented in figures and should be clearly labelled with the figure number in the file title. The source gel images should be uncropped, unmodified images, with molecular weight markers. Uncropped, unmodified gel images from existing experimental repeats, in addition to the data shown in the figures, can also be presented in source data. Please provide these files as PDF with your revision.

Please ensure that all control panels for gels and western blots are appropriately described as loading or IP controls. Lastly, please ensure that all images in the paper are checked for duplication of panels and for splicing of gel lanes.

6. SOURCE NUMERICAL DATA: we urge authors to provide, in tabular form in excel files, the data underlying the graphical representations used in figures. This is to further increase transparency in data reporting, as detailed in this editorial (http://www.nature.com/nsmb/journal/v22/n10/full/nsmb.3110.html). Please provide one excel file per figure max, with one panel per tab. When submitting files, the title field should indicate which figure the source data pertains to.

7. DATA AVAILABILITY: this journal strongly supports public availability of data. All data used in accepted papers should be available via a public data repository, or alternatively, as Supplementary Information. If data can only be shared on request, please explain why in your Data Availability Statement and in the correspondence with your editor.

For some data types, deposition in a public repository is mandatory as detailed below:

https://www.nature.com/nature-research/editorial-policies/reporting-standards#availability-of-data

Finally, please ensure that you retain unprocessed data and metadata files after publication, ideally archiving data in

perpetuity, as these may be requested during the peer review and production process or after publication if any issues arise.

EXTENDED DATA FIGURES

When re-submitting your manuscript, please ensure that any supplementary figures and tables that are crucial to the manuscript's conclusions are converted into Extended Data figures and tables to increase visibility of these data. Extended Data figures and tables are online-only (present in the online PDF and full-text HTML versions of the paper), peer-reviewed display items that provide essential background to the article but are not included in the main article due to space constraints. A maximum of ten Extended Data display items (figures and tables) is permitted.

While we encourage the use of color in preparing figures, please note that this will incur a charge to partially defray the cost of printing. Information about color charges can be found at http://www.nature.com/nsmb/authors/submit/index.html#costs

Nature Structural & Molecular Biology is committed to improving transparency in authorship. As part of our efforts in this direction, we are now requesting that all authors identified as 'corresponding author' on published papers create and link their Open Researcher and Contributor Identifier (ORCID) with their account on the Manuscript Tracking System (MTS), prior to acceptance. This applies to primary research papers only. ORCID helps the scientific community achieve unambiguous attribution of all scholarly contributions. You can create and link your ORCID from the home page of the MTS by clicking on 'Modify my Springer Nature account'. For more information please visit please visit www.springernature.com/orcid.

Please use the link below to submit your revised manuscript and related files:

Link Redacted

Note: This URL links to your confidential home page and associated information about manuscripts you may have submitted, or that you are reviewing for us. If you wish to forward this email to co-authors, please delete the link to your homepage.

We look forward to seeing the revised manuscript and thank you for the opportunity to review your work.

Sincerely,

Dimitris Typas
Senior Editor
Nature Structural & Molecular Biology
ORCID: 0000-0002-8737-1319

Reviewers' Comments:

Reviewer #1 (Remarks to the Author):

Overview:

This manuscript delivers a deep empirical phosphoproteome library across 33 human cell lines and demonstrates its practical benefits for DIA phosphoproteomics, including improved depth, particularly for limited input, substantial reductions in processing time when utilizing DIA-NN for data analysis, and a new kinase activity calculation (Cscore) that takes into account the expression of kinase proteins within a given sample. The breadth of this phosphopeptide spectral library is impressive and will undoubtedly serve as a highly valuable resource for the proteomics community. However, while various descriptive characterizations are performed on the dataset, there are cases where they appear incompletely evaluated, lack quantitative metrics of evaluation that include informative controls, or result in predictable findings. Some examples of this are further described in my specific comments below, and include topics regarding specificity of phosphorylation sites across cell lines, the proposed impact of poor MS2 spectral quality in low input phosphorylation experiments, and the functional analysis across cell lines. Overall, this manuscript presents a highly valuable spectral library resource, but would be strengthened by focusing on only a selection of the topics covered currently, and including a more thorough evaluation of such focused topics.

Specific comments:

I feel this statement to be misleading: "However, extracting meaningful biological information requires datasets that are as complete and comprehensive as possible". While certainly having larger datasets can be helpful, it is false to say that they are required to extract meaningful biological information. Countless studies with lower levels of completeness (from the author's own lab included) have been able to reveal meaningful and impactful biology.

Figure 1B has "Ti-IMAC" in the figure, yet the text describes that Zr-IMAC is used, and the methods section described it at Ti-IMAC. Clearly there is an error in one or more of these descriptions/labels. Additionally, as Figure 1 is describing the combined results of both the protein abundance and phosphorylation analysis, it would likely be more accurate if this schematic also included the protein abundance measurements.

As noted in the comparison across cell lines (Figure 2D and Supplementary Figure 4), artifacts related to FDR control may be influencing these differences, and it is difficult to know to what extent that is true. To help to assess this, I would suggest a comparison of replicates within a cell line. For example, for each set of 4 replicates of a given cell line and a given treatment, evaluate the pairwise overlap in identifications. Plotting this distribution relative to the distribution of overlap across cell lines would be a valuable additional panel to Figure 2, and serve to set a benchmark for technical vs. cell line biology differences. Ideally, the distribution of differences between replicates would be lower than across cell lines, and would provide for the legitimacy of differences observed across cell lines.

Regarding the aspect of more IDs using a library as compared to library free, is this not an expected result? If I am not mistaken, this result has been previously observed by this lab (PMID: 32034161).

Figure S9B. When a dot is grey, that means it's just not part of the condition group that is colored? Is that correct?

Figure 3. Text and figures say 100 SPD method used; Figure legend says 30, 60, 80, and 120 SPD.

Figure 3A. Could you clarify the input amount? (Given as 108-9 ug)

Figure 3C. For library-based searches, does this time include library generation?

The claim that directDIA's higher completeness reflects noisier integration is plausible but not conclusively proven without an orthogonal check. Please support the interpretation that directDIA is integrating noise vs true phosphorylation by reporting additional quality metrics (peak shape, score distribution, etc) for those cases.

Figure 5. I question the general utility of this figure and section of text in the paper. My intuition is that a 10-minute serum-starvation experiment is not a compelling experiment to demonstrate the importance/need/benefit for a stoichiometry calculation to determine biologically informative phosphorylation sites. I would assume with such a short stimulation, minimal changes in total protein abundance are observed, and that similar observations would be observed by a standard quantitative comparison of phosphorylation site abundance changes? Is this true?

Figure 5G. The grey color is not defined. What do these mean?

Figure 6. I don't know how impactful this analysis is, as it largely describes expected results of the tissue or disease origin of cell lines, such as findings that "blood cancer cell lines was characterized by the overexpression of proteins associated with the hematopoietic system and immune cell activation."

Figure 7. I think it would be more clear if all references in the figure used "Cscore" instead of Kinase score, as if I am not mistaken, these are the same things?

Figure 7D. Is this the definition of "outliers" from the text: "Cell-line specific vulnerabilities were investigated through the identification of kinase-cell line pairs in which a kinase in a given cell line either showed activity substantially higher than its activity distribution across all other cell lines"?

I understand the rationale behind wanting to promote the importance of stoichiometry for prioritizing which phosphorylation sites are most functionally important, but I would encourage the authors to use less rigid language here. For example the discussion describes this as "vital" which is defined as something that is absolutely necessary or essential. I think that this overly simplifies the complexity of proteome regulation. One, it takes a gene centric approach, which we know is inadequate for a proteoform level question, where the presence of a PTM can allow a protein to take on an entirely new cellular function. Secondly, it overlooks the role of additional dimensions to function, such as the local stoichiometry of a PTM, either within a given subcellular compartment, protein complex, etc, where even small changes in stoichiometry (at a global level) may be concentrated to represent substantial changes in a local environment.

This section of the methods does not make sense to me. Why are there two different columns and two different sources described?: "All samples were analysed on the Evosep One system (Evosep Biosystems, Denmark) coupled to an Orbitrap-Astral mass spectrometer (Thermo Fisher Scientific, Germany). Standard gradients were used with commercial Evosep columns (30 SPD (samples per day) with EV1137: 15 cm x 150 µm, 1.5 µm 100 SPD with EV1109: 8 cm x 150 µm, 1.5 µm) connected to a stainless steel emitter (30 µm i.d., Evosep, EV1086), with temperature set to 40°C through an externally controlled butterfly heater (MSWil, Cat#PST-ES-BPH-20). The columns were interfaced to the mass spectrometer using an EASY-Spray source. Whisper zoom gradients were used with Aurora Rapid columns (5 cm x 75 µm, 1.7 µm) interfaced to the mass spectrometer using a Nanospray Flex source, with column temperature maintained at 50°C."

There appear to be no supplementary tables included with this manuscript. Supplemental tables should be included that contain key datasets, such as the full list of phosphorylation sites, phosphorylated peptides, proteins and their quantitative abundances across cell lines and individual replicates. Likely additional tables of key data from other figures would be helpful as well, such as the kinase activity scores across cell lines.

Please further define what was done for tail-based imputation.

For phosphorylation experiments, cross run normalization was enabled in the Spectronaut analysis, but then additional

mean centering normalization is performed in the data processing phase? Why does normalization need to be performed twice? Is this due to imputation?

Each cell line is searched separately, then merged. Does this have an effect on FDR? This is briefly mentioned in the text in relation to phospho tyrosine signaling ("it cannot be excluded that it could be an artifact related to FDR control across searches performed separately and subsequently merged") but this is not expanded on.

Signed,

Danielle Swaney
Associate Professor
UCSF

Reviewer #2 (Remarks to the Author):

This manuscript presents an exceptionally deep and technically sophisticated human cell line phosphoproteome atlas, comprising >223,000 class I phosphosites across 33 diverse human cell lines. By leveraging narrow-window DIA on the Orbitrap Astral and constructing a large empirical phosphopeptide spectral library, the authors convincingly demonstrate improvements in phosphosite coverage, site localization confidence, and computational efficiency. The work further integrates proteome and phosphoproteome data to derive a combined kinase activity score (Cscore), enabling cell line–specific signaling inference and correlation with drug sensitivity.

Overall, this is a high-quality, well-executed, and highly valuable community resource that clearly fits the Resource format of Nature Structural & Molecular Biology. The dataset will be broadly useful for signaling biology, (phospho)proteomics-centered method development, drug discovery, and translational research.

Comments:
1. The empirical phosphopeptide library generated from 33 diverse human cell lines represents a major strength of this work. To further guide future users, it would be helpful for the authors to briefly discuss the expected scope and potential limitations of the library when applied to biological contexts not represented in the panel (e.g., different disease or special tissue samples). A short discussion or summary analysis highlighting pathway, motif, or kinase-family coverage would further enhance transparency and usability.

2. The combined kinase activity score (Cscore) is a valuable conceptual advance that improves kinase activity inference, particularly for tyrosine kinases. The authors may need to provide detailed information to clarify how to combine different scoring method into Cscore. To further support its general adoption, the authors could add a short clarification regarding the relative contribution of the different score components and comment on robustness across kinases with varying levels of annotation.

3. The large-scale estimation of phosphorylation site stoichiometry is an impressive addition to the dataset. Given the inherent experimental and computational complexity of stoichiometry measurements, a short clarification on the interpretational boundaries of absolute stoichiometry values (e.g., sensitivity to measurement noise, emphasis on relative trends) would help readers appropriately contextualize these results. Additionally, it is also interesting to comment if there is any specific quantitative threshold to activate a signaling event.

Reviewer #3 (Remarks to the Author):

In the manuscript 'The Human Cell Line Phosphoproteome Atlas: A Deep Empirical Resource Revealing Kinase Activity Landscapes', by Koenig et al. the authors present an exceptionally large dataset coverage (mean of 30,498 phosphosites per run; 223,887 distinct phosphosites identified and 110,291 phosphosites quantified across 33 diverse cell lines), with appropriate and thought-out experimental design. The obtained empirical spectral library is a valuable resource demonstrating clear utility in improving identification depth, computational efficiency, and reducing false positives in directDIA approaches. The authors have also achieved a comprehensive benchmarking of the library across LC gradients and search engines. Furthermore, their novel kinase activity analysis identified key limitations of existing methods and proposed improvements in vulnerability assessment.

Major comments

• FDR control in merged results
Searches were performed separately per cell line, then merged for library generation. This raises concerns about whether the merged results maintain proper FDR control, particularly for phosphotyrosine sites where sensitivity varies across cell lines. It would be helpful if the authors could discuss in the manuscript regarding the considerations taken to ensure their FDR is indeed properly controlled.

• Cell line-specific treatments
Pervanadate treatment was applied to only DLD-1 to boost pTyr detection, but the paper does not explain why this was cell-line-specific or whether selective enrichment in one cell line biases the merged library's pTyr composition.

Also the methods section shows some cell lines were treated differently (length of serum starvation, one line not starved, etc) – does this affect the comparisons and inferences made later?

• Stoichiometry analysis
Stoichiometry was calculated for 1,580 phosphosites on average per cell line, which is only ~5% of phosphosites detected per cell line. For those sites it is valuable information but as the authors highlight in the discussion, since most sites exhibit low occupancy, stoichiometry calculations remain challenging even when there is a big change between conditions.

The analysis of kinases and signalling events in Fig 5 is done on stoichiometry data – so how much does such analysis provide additional insight into signalling upon serum stimulation, compared to just using relative quantification values, for which the data has so much higher coverage? An explicit comparison is not presented but might add helpful guidance to the community.

The logic for doing this analysis on stoichiometry data only might need some clarification. The premise is that stoichiometry is important because, as authors say, "a large stoichiometry for a dynamically regulated site strongly suggests it is functionally important." But kinase inference uses fold-change, not absolute stoichiometry, so both low and high occupancy sites would be ranked equally?

• Cell line clustering
Consensus clustering was performed on proteome data and kinase activity inference later in the study. Wouldn't additionally direct clustering on phosphosite-level data reveal signaling-specific groups independent of kinase inference assumptions, potentially uncovering kinase-independent signaling events or phosphoproteome complexity not captured at the kinase level?

• Kinase activity scoring
Kinase activity inference is a valuable part of the study, where comparing existing methods with biological expectations identifies areas where improvements are needed. However, the Cscore may require additional considerations.

The formula used for Cscore appears to treat its components - kinase protein abundance, activation loop phosphorylation, and substrate phosphorylation - as independent evidence streams, which may not be biologically correct. For example, activation loop phosphorylation could gate substrate activity rather than complementing it. The fixed combining strategy could also overweight noisy inputs or underweight informative ones, as the score components have different scales and noise properties.

To elaborate, would this method distinguish (i) an abundant but autoinhibited kinase with few substrates from (ii) a moderately expressed, activated kinase with moderate substrates? Only the latter represents genuine activity. So a near-zero score could conflate kinase absence (truly inactive), lack of activation loop phosphorylation (could be autoinhibited or unstimulated), and few detected substrates (method limitation). These are biologically distinct scenarios but relying on the score alone makes them mathematically indistinguishable.

• Drug sensitivity/vulnerability assessment
The use of kinase activity scoring to assess drug-sensitivity shows qualitative, cell line–specific patterns well. However, to claim identifying therapeutic vulnerabilities rather than just signalling correlates, the study could further benefit from orthogonal functional tests in matched conditions demonstrating that high-scoring kinases are true dependencies (e.g. CRISPR or degrader-based causal links).

• Data Accessibility
Importantly, while the raw data is deposited on PRIDE, for the wider community to benefit from this rich resource, authors should provide the analysis results details - incl. abundances, stoichiometry, kinase analysis - in a more accessible manner for the final publication, e.g. as annotated supplementary tables (or optionally as a web resource in addition).

Minor corrections

Fig2D: D- Upset plot presenting the phosphosite overlap across all 33 human cell lines analyzed and the pervanadate treatment. -> rephrase

Additionally, phosphorylation can only be calculated when a change is observed between both conditions. -> correct for 'stoichiometry can only be calculated…'

Additionally, DLD-1 was treated with pervanadate (PV) for 15 minutes using 0.5 mM and 1mM of PV. -> correct the concentration used

Version 1:

Decision Letter:

Our ref: NSMB-RS52225A

18th Jun 2026

Dear Professor Olsen,

Thank you for submitting your revised manuscript "The Human Cell Line Phosphoproteome Atlas: A Deep Empirical Resource Revealing Kinase Activity Landscapes" (NSMB-RS52225A). It has now been seen by the original referees and their comments are below. The reviewers find that the paper has improved in revision, and therefore we can accept it in principle in Nature Structural & Molecular Biology, pending revisions to satisfy the referees' final requests-the toning down in the points requested by Reviewer #1 and to a lesser degree Reviewer #3 will have to be explicit, convincing and extensive- and to comply with our editorial and formatting guidelines.

We are now performing detailed checks on your paper and will send you a checklist detailing our editorial and formatting requirements in a couple of weeks. Please do not upload the final materials and make any revisions until you receive this additional information from us.

To facilitate our work at this stage, it is important that we have a copy of the main text as a word file. If you could please send along a word version of this file as soon as possible, we would greatly appreciate it; please make sure to copy the NSMB account (cc'ed above).

Thank you again for your interest in Nature Structural & Molecular Biology Please do not hesitate to contact me if you have any questions.

Sincerely,

Dimitris Typas
Senior Editor
Nature Structural & Molecular Biology
ORCID: 0000-0002-8737-1319

Reviewer #1 (Remarks to the Author):

I appreciate the efforts by the authors to address the critiques of the first review and find this to be a significantly improved manuscript. Below are a few text edits that I would recommend:

1. While the authors have removed the "as a large stoichiometry for a dynamically regulated site strongly suggests that it is functionally important" at the beginning of section IV as suggested, they have essentially replaced the spirit of that with just longer text in: "because it reveals the actual fraction of a protein population modified at a specific site, offering a true measure of signaling activity. In contrast, relative fold-changes alone can be misleading as the same fold-change may represent either a trivial or a highly significant biological event depending on the baseline occupancy. Moreover, fold-changes are confounded by changes in total protein abundance and do not distinguish between true phosphorylation dynamics and protein expression changes. Site stoichiometry estimation, which integrates both phosphorylated and unphosphorylated forms, provides the necessary context for accurate biological interpretation, functional analysis, and systems-level modeling."

There are so many nuances to the underlying biology of this calculation that can lead to inaccurate biological interpretations of the calculations (e.g. subcellular localization specific impacts on phos state, divergent regulation of individual phos sites on the same peptide, etc.) that my advice would be more diplomatic about this, and simply state something to the effect of "stoichiometry integrates both phosphorylated and unphosphorylated forms and therefore offers a complementary context under which one can perform biological interpretation, functional analysis, and systems-level modeling."

2. "This also establishes that regulatory pY sites have very low baseline occupancy, which explains why antibodies are often needed for specific enrichment of tyrosine-phosphorylated peptides for global profiling by LC-MS (Lundby et al. 2019)." I think "establishes" is not the correct word here, this concept is already well-established, I think "supports" would be more appropriate.

3. Regarding this statement: "This highlights the challenge of interpreting drug screens, particularly when drug selectivity is not well described and off-target effects can interfere with the results". While this is certainly true, I would advise that it should be also balanced with a statement that kinase activity scores are also messy, as there is a lot of overlap in substrates between kinases. That is, both the inhibitors, and our ability to attribute phosphorylation site changes to a given kinase are complicated and imperfect.

Signed,

Danielle Swaney
UCSF

Reviewer #2 (Remarks to the Author):

I appreciate the authors' efforts in addressing all of my comments. The manuscript has been substantially improved, and I recommend publication of this valuable resource article to share with the community.

YuJu Chen

Reviewer #3 (Remarks to the Author):

The authors have done a thorough job at addressing my concerns and I am satisfied with their responses.

My final comment concerns the FDR analysis of the merged search results for this phosphoproteome atlas. FDR control is inherently challenging and the authors have clearly made a thorough effort to manage this by using indirect evaluation methods and maintaining a conservative approach. While determining the true FDR remains an open challenge that falls outside the scope of this study, it represents a broader issue that the community must eventually address. I would appreciate it if the authors could acknowledge this point in their discussion.

Reviewer #3 (Remarks on code availability):

The code on the github site is well annotated and structured according to type of analysis.

INTERNAL USE ONLY AIP modules required: ["Life Science"]

Version 2:

Decision Letter:

4th Aug 2026

Dear Professor Olsen,

We are now happy to accept your revised paper "A phosphoproteome atlas of human cell-lines reveals the landscape of kinase activity" for publication as a Resource in Nature Structural & Molecular Biology.

Acceptance is conditional on the manuscript's not being published elsewhere and on there being no announcement of this work to the newspapers, magazines, radio or television until the publication date in Nature Structural & Molecular Biology.

Over the next few weeks, your paper will be copyedited to ensure that it conforms to Nature Structural & Molecular Biology style. Once your paper is typeset, you will receive an email with a link to choose the appropriate publishing options for your paper and our Author Services team will be in touch regarding any additional information that may be required.

After the grant of rights is completed, you will receive a link to your electronic proof via email with a request to make any corrections within 48 hours. If, when you receive your proof, you cannot meet this deadline, please inform us at rjsproduction@springernature.com immediately.

You will not receive your proofs until the publishing agreement has been received through our system.

Due to the importance of these deadlines, we ask that you please let us know now whether you will be difficult to contact over the next month. If this is the case, we ask you provide us with the contact information (email, phone and fax) of someone who will be able to check the proofs on your behalf, and who will be available to address any last-minute problems.

To assist our authors in disseminating their research to the broader community, our SharedIt initiative provides all co-authors with the ability to generate a unique shareable link that will allow anyone (with or without a subscription) to read the published article. Recipients of the link with a subscription will also be able to download and print the PDF.

As soon as your article is published, you can generate your shareable link by entering the DOI of your article here: http://authors.springernature.com/share. Corresponding authors will also receive an automated email with the shareable link

Note the policy of the journal on data deposition: http://www.nature.com/authors/policies/availability.html.

Your paper will be published online soon after we receive proof corrections and will appear in print in the next available

issue. You can find out your date of online publication by contacting the production team shortly after sending your proof corrections.

You may wish to make your media relations office aware of your accepted publication, in case they consider it appropriate to organize some internal or external publicity. Once your paper has been scheduled you will receive an email confirming the publication details. This is normally 3-4 working days in advance of publication. If you need additional notice of the date and time of publication, please let the production team know when you receive the proof of your article to ensure there is sufficient time to coordinate. Further information on our embargo policies can be found here: https://www.nature.com/authors/policies/embargo.html

You can now use a single sign-on for all your accounts, view the status of all your manuscript submissions and reviews, access usage statistics for your published articles and download a record of your refereeing activity for the Nature journals.

If you have not already done so, we strongly recommend that you upload the step-by-step protocols used in this manuscript to the Protocol Exchange. Protocol Exchange is an open online resource that allows researchers to share their detailed experimental know-how. All uploaded protocols are made freely available, assigned DOIs for ease of citation and fully searchable through nature.com. Protocols can be linked to any publications in which they are used and will be linked to from your article. You can also establish a dedicated page to collect all your lab Protocols. By uploading your Protocols to Protocol Exchange, you are enabling researchers to more readily reproduce or adapt the methodology you use, as well as increasing the visibility of your protocols and papers. Upload your Protocols at www.nature.com/protocolexchange/. Further information can be found at www.nature.com/protocolexchange/about.

An online order form for reprints of your paper is available at https://www.nature.com/reprints/author-reprints.html. Please let your coauthors and your institutions' public affairs office know that they are also welcome to order reprints by this method.

Authors may need to take specific actions to achieve compliance with funder and institutional open access mandates. If your research is supported by a funder that requires immediate open access (e.g. according to Plan S principles or the NIH public access policy) then you should select the gold OA route, and we will direct you to the compliant route where possible. Because authors warrant under our subscription licensing terms that they haven’t committed to licensing any version of their article under a licence inconsistent with the terms of our agreement – including the applicable embargo period – publication under the subscription model isn’t suitable for authors whose funders require no embargo.

In approximately 10 business days you will receive an email with a link to choose the appropriate publishing options for your paper and our Author Services team will be in touch regarding any additional information that may be required.

You will not receive your proofs until the publishing agreement has been received through our system.

If you have any questions about our publishing options, costs, Open Access requirements, or our legal forms, please contact ASJournals@springernature.com

Sincerely,

Dimitris Typas
Senior Editor
Nature Structural & Molecular Biology
ORCID: 0000-0002-8737-1319

Click here if you would like to recommend Nature Structural & Molecular Biology to your librarian: http://www.nature.com/subscriptions/recommend.html#forms

Open Access This Peer Review File is licensed under a Creative Commons Attribution 4.0 International License, which permits use, sharing, adaptation, distribution and reproduction in any medium or format, as long as you give appropriate credit to the original author(s) and the source, provide a link to the Creative Commons license, and indicate if changes were made.
In cases where reviewers are anonymous, credit should be given to 'Anonymous Referee' and the source.
The images or other third party material in this Peer Review File are included in the article's Creative Commons license, unless indicated otherwise in a credit line to the material. If material is not included in the article's Creative Commons license and your intended use is not permitted by statutory regulation or exceeds the permitted use, you will need to obtain permission directly from the copyright holder.
To view a copy of this license, visit https://creativecommons.org/licenses/by/4.0/

Point-by-point responses to reviewer comments

Nature Structural & Molecular Biology submission: **NSMB-RS52225**
Title: *The Human Cell Line Phosphoproteome Atlas: A Deep Empirical Resource Revealing Kinase Activity Landscapes*

Authors: Claire Koenig, Hayoung Cho, Kristina B. Emdal, Ilaria Piga, Pierre Sabatier, Samuel Lozano-Juárez, Ana Martinez-Val and Jesper V. Olsen*

General response to reviewers and editor:

We have carefully gone through all of the comments made by the reviewers and addressed them point-by-point below. Our answers to the reviewer questions are indicated in blue text*. We would like to thank the reviewers for their valuable input and suggestions to the manuscript, which we have updated accordingly. This helped us improve our manuscript during this process.*

Reviewer #1 (Remarks to the Author):

Overview:

This manuscript delivers a deep empirical phosphoproteome library across 33 human cell lines and demonstrates its practical benefits for DIA phosphoproteomics, including improved depth, particularly for limited input, substantial reductions in processing time when utilizing DIA-NN for data analysis, and a new kinase activity calculation (Cscore) that takes into account the expression of kinase proteins within a given sample. The breadth of this phosphopeptide spectral library is impressive and will undoubtedly serve as a highly valuable resource for the proteomics community. However, while various descriptive characterizations are performed on the dataset, there are cases where they appear incompletely evaluated, lack quantitative metrics of evaluation that include informative controls, or result in predictable findings. Some examples of this are further described in my specific comments below, and include topics regarding specificity of phosphorylation sites across cell lines, the proposed impact of poor MS2 spectral quality in low input phosphorylation experiments, and the functional analysis across cell lines. Overall, this manuscript presents a highly valuable spectral library resource, but would be strengthened by focusing on only a selection of the topics covered currently, and including a more thorough evaluation of such focused topics.

1. We appreciate Reviewer #1's thorough feedback and constructive suggestions for improving the manuscript. We would like to thank the reviewer for the insightful

comments, and consequently, we have put efforts into addressing her concerns with detailed answers to the specific comments below.

Specific comments:

I feel this statement to be misleading: “However, extracting meaningful biological information requires datasets that are as complete and comprehensive as possible”. While certainly having larger datasets can be helpful, it is false to say that they are required to extract meaningful biological information. Countless studies with lower levels of completeness (from the author’s own lab included) have been able to reveal meaningful and impactful biology.

2. The reviewer is right, the statement is a bit misleading. We agree that impactful biology can often be derived from smaller datasets. We revised the statement to acknowledge that while larger datasets can provide greater coverage, completeness and context facilitating biological interpretation, meaningful biological insights can indeed be revealed from less comprehensive datasets. The sentence has been rephrased into: “However, extracting meaningful biological information can be facilitated with larger datasets providing greater coverage and completeness.”

Figure 1B has “Ti-IMAC” in the figure, yet the text describes that Zr-IMAC is used, and the methods section described it at Ti-IMAC. Clearly there is an error in one or more of these descriptions/labels. Additionally, as Figure 1 is describing the combined results of both the protein abundance and phosphorylation analysis, it would likely be more accurate if this schematic also included the protein abundance measurements.

3. Thank you for noticing our mistake. We have indeed used Ti-IMAC HP beads, and we have now corrected the text accordingly. The workflow in Figure 1 was modified to make explicit the generation of both proteomics and phosphoproteomics samples. However, the results presented in Figure 1 only represent proteome-level results, except for Figure 1D including both predicted cellular localization of the protein and phosphoproteins.

As noted in the comparison across cell lines (Figure 2D and Supplementary Figure 4), artifacts related to FDR control may be influencing these differences, and it is difficult to know to what extent that is true. To help to assess this, I would suggest a comparison of replicates within a cell line. For example, for each set of 4 replicates of a given cell line and a given treatment, evaluate the pairwise overlap in identifications. Plotting this distribution relative to the distribution of overlap across cell lines would be a valuable additional panel to Figure 2, and serve to set a benchmark for technical vs. cell line biology differences. Ideally, the distribution of differences between replicates would be lower than across cell lines, and would provide for the legitimacy of differences observed across cell lines.

4. This is an excellent suggestion. To assess the contribution of technical variability, we computed all pairwise overlaps of quantified phosphosites both within conditions (replicates) and across cell lines. For each condition (33 cell lines + PV-treated DLD-1, each in control and stimulated states), we calculated pairwise overlaps between the 4 replicates, yielding 408 comparisons representing technical variability. In parallel, we

computed overlaps across cell lines, resulting in 561 comparisons representing biological variation.

The distribution of those overlaps, either across or within cell lines, showed a higher overlap within cell lines and conditions compared to across cell lines (**Figure R1**). As a consequence, technical variability, stochasticity and FDR cannot only be responsible for the site uniqueness observed across cell lines in the upset plot. Additionally, the overlap distribution across cell lines is broader, as expected, with the wide diversity of cell lines included in our panel.

Importantly, these analyses were performed on datasets filtered for valid values, ensuring that only confidently quantified sites were considered. Altogether, these results indicate that the uniqueness in sites quantified per cell line is likely cell line-specific signalling rather than FDR artefacts.

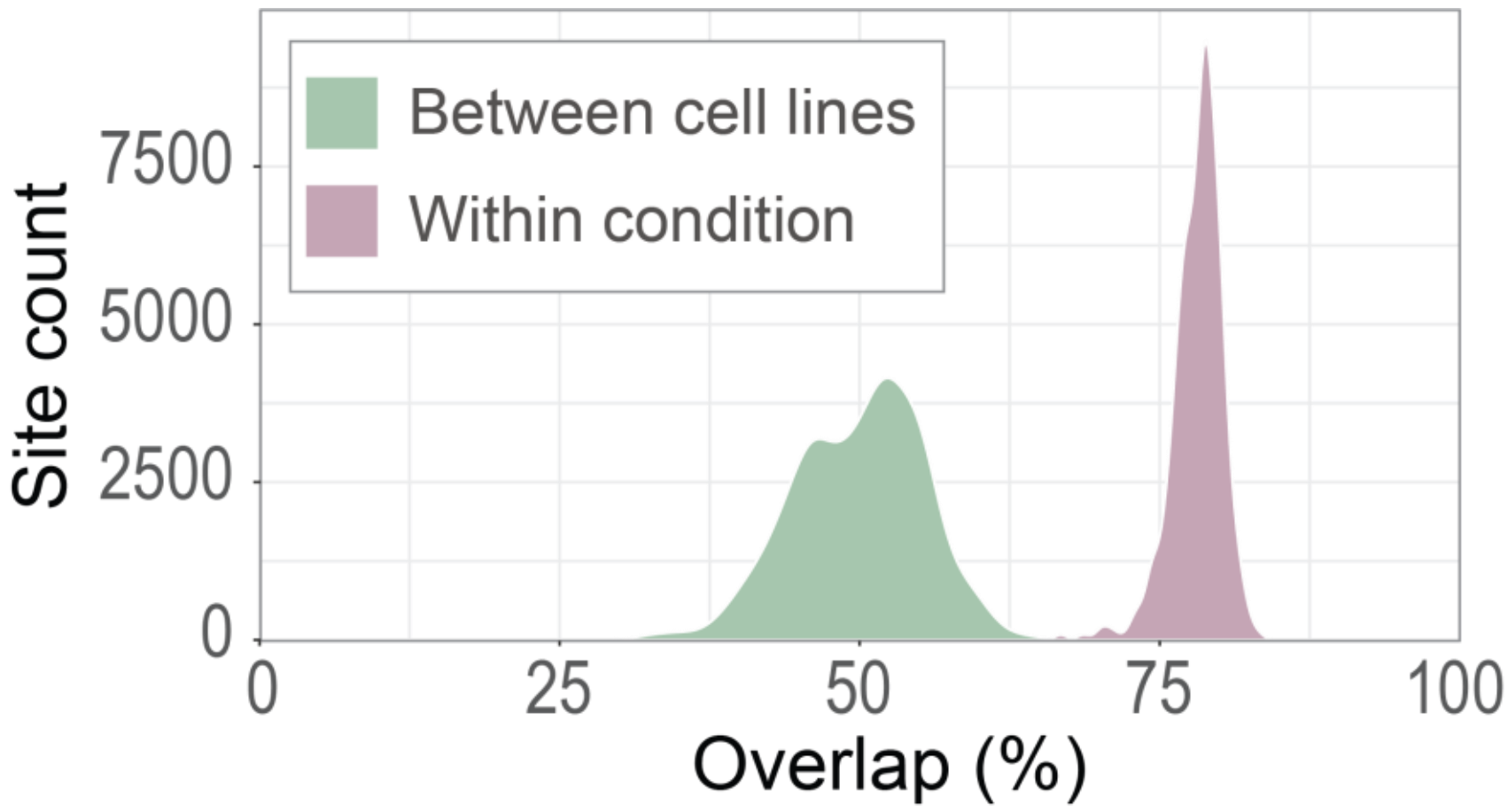

Figure R1: Distribution of pairwise overlaps either between cell lines or within conditions (cell line + treatment).

Regarding the aspect of more IDs using a library as compared to library free, is this not an expected result? If I am not mistaken, this result has been previously observed by this lab (PMID: 32034161).

5. We agree that the higher number of identifications using a library is expected and has been observed previously, including in our prior work, the original directDIA paper applied to phosphoproteomics. This paper demonstrated the possibility of analysing DIA-based phosphoproteomics data without the need for a library. However, in the past six years, since this paper was published, there has been major improvements in both the directDIA algorithm, and MS instruments allowing for narrow-window DIA. The result was expected but not obvious. Moreover, this statement does not just reflect the benefit of using a library-based search strategy; it was intended to demonstrate the completeness of the generated library vs an unbiased approach in the case where full site populations were missing from the library. To clarify this point, we will explicitly cite our previous study (PMID: 32034161) and acknowledge this in the discussion by rephrasing the sentence into:

" While the use of a spectral library for DIA-based phosphoproteomics was the standard approach before the emergence of directDIA, our results showed that our highly complete empirical phosphopeptide spectral library can still substantially improve DIA-based phosphoproteomics, without the need for fractionation (Bekker-Jensen et al. 2020)."

Figure S9B. When a dot is grey, that means it's just not part of the condition group that is colored? Is that correct?

6. Yes, it is the case. We have updated the legend for more clarity by labelling the grey dots as "All data".

Figure 3. Text and figures say 100 SPD method used; Figure legend says 30, 60, 80, and 120 SPD.

7. Thank you for noticing our error. The figure is correct, and we have used 100 SPD for the benchmark and not 60 SPD. It has now been corrected in the legend.

Figure 3A. Could you clarify the input amount? (Given as 108-9 ug)

8. We have added information on figure 3A to make it clear that 108ug peptides were used as an input for the 30 and 100 SPD gradients, and 9ug of peptides were enriched for the 80 and 120 SPD gradients.

Figure 3C. For library-based searches, does this time include library generation?

9. The search times were calculated excluding the time for library generation. It is true that the initial processing time for library generation was substantial. However, we provide the library as a resource, which means that it doesn't have to be processed again by anyone wanting to use it. We here compare the search times of a user having access to the generated library.

The claim that directDIA's higher completeness reflects noisier integration is plausible but not conclusively proven without an orthogonal check. Please support the interpretation that directDIA is integrating noise vs true phosphorylation by reporting additional quality metrics (peak shape, score distribution, etc) for those cases.

10. We agree that only reporting the proportions of the type of missingness upon EGF stimulation is not enough to claim that directDIA leads to the integration of noisier signals. We have added an extended data figure to further demonstrate this point. We are first displaying the MS2 traces of the EGFR autophosphorylation site EGFR_Y1110 across input amount and conditions (**Figure R2A**). Based on the MS traces, it is possible to visually see that EGFR_Y1110 is absent from the control and present upon EGF stimulation in the raw files. However, site quantification output tables are showing EGFR_Y1110 as present in the control in most of the directDIA results, while totally absent from the control in the library-based results. Therefore, what we can visually identify as noise led to a quantitative value for directDIA outputs.

We further looked into quality metrics to demonstrate our point. We have in **figure R2B** plotted the distribution of the log2-transformed extracted ion current (XIC) of the noise level calculated as the average sum XIC intensity outside of the peak boundary (EG.Noise). While the noise level scales up with the input amount, the noise distribution is wider and spreading on the higher range in directDIA compared to library-based. This demonstrates the quantification of signals within a higher noise background with directDIA, which combined with **Figure R2A** hints towards the integration of noise as signal rather than the detection of lower abundant features within a noisier background.

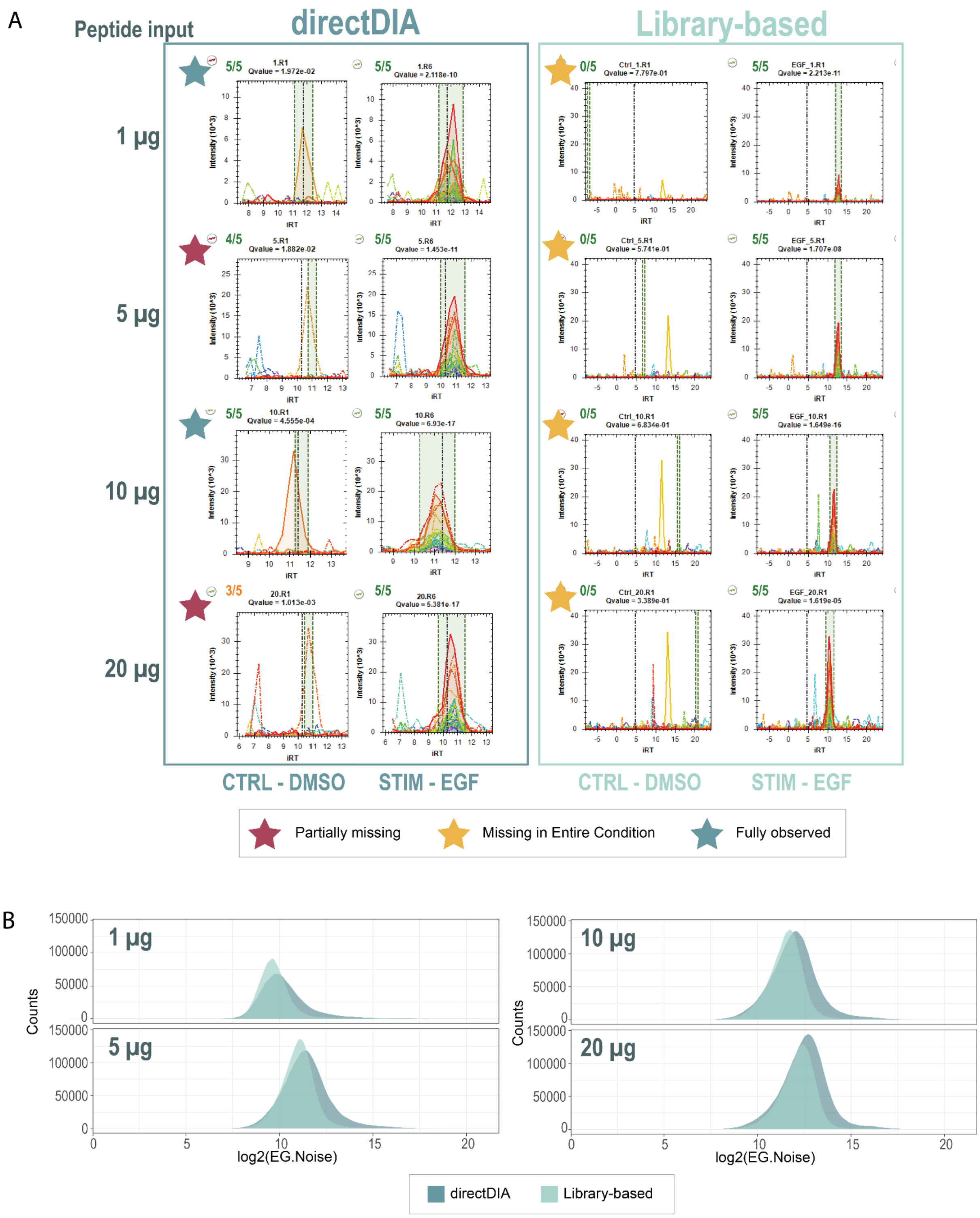

Figure R2: DirectDIA results in the integration to noisier signals compared to a library-based approach. A- MS2 traces of the main precursor covering EGFR_Y1110 in both the control and EGF stimulated conditions searched with directDIA or our spectral library. The colored stars represent the type of missingness of EGFR_Y1110 (partially missing, missing in an entire condition or fully observed). EGFR_Y1110 is an activation site and should only be detected phosphorylated after EGF stimulation. The green value represents the number of replicates out of 5 in which the site was quantified. B - Distribution of the log2 transformed estimated XIC noise level, calculated as the average sum XIC intensity outside of the peak boundary, between directDIA and the library-based approach. While the noise level scales with the input amount, the estimated noise level distribution is wider and spreading on the higher range in directDIA vs. library-based.

Figure 5. I question the general utility of this figure and section of text in the paper. My intuition is that a 10-minute serum-starvation experiment is not a compelling experiment to demonstrate the importance/need/benefit for a stoichiometry calculation to determine biologically informative phosphorylation sites. I would assume with such a short stimulation, minimal changes in total protein abundance are observed, and that similar observations would be observed by a standard quantitative comparison of phosphorylation site abundance changes? Is this true?

11. We acknowledge that a 10-minute stimulation may not fully demonstrate the utility of stoichiometry calculations. We have indeed used a short stimulation time to avoid protein abundance changes, which would complicate the stoichiometry calculation and reduce the precision. To demonstrate the utility of stoichiometry here, we have performed a similar analysis from the log2-fold-change intensities (**Figure R3 A-C**). Per cell line, limma statistical testing was applied to the control and stimulated conditions. Sites with Benjamini-Hochberg corrected p-values below 1% were considered statistically significantly regulated. The up-regulated versus down-regulated sites were used for the motif analysis and kinase enrichment analysis using Rokai, PTM-SEA, and the Kinase Library was carried out on the regulated sites only. We indeed can find similar results. However, the agreement between intensity- and stoichiometry-based regulatory analysis adds confidence in the stoichiometry calculation analysis, and thus, ultimately prove it valuable as a resource providing a site-specific baseline of absolute occupancy within a cell. This would be of high value for the community studying occupational changes for functional activation. We have added further analysis to this section highlighting a high proportion of kinases primarily facilitating phosphorylation to low occupancy values, while other kinases would primarily lead to high occupancy values (**Figure R3 D**). Additionally, we could demonstrate a general trend where pY regulatory sites would be phosphorylated with overall lower stoichiometries and still result in overall higher fold changes (**Figure R4**), highlighting their crucial regulatory functions within cells and the need for occupancy-based activation thresholds.

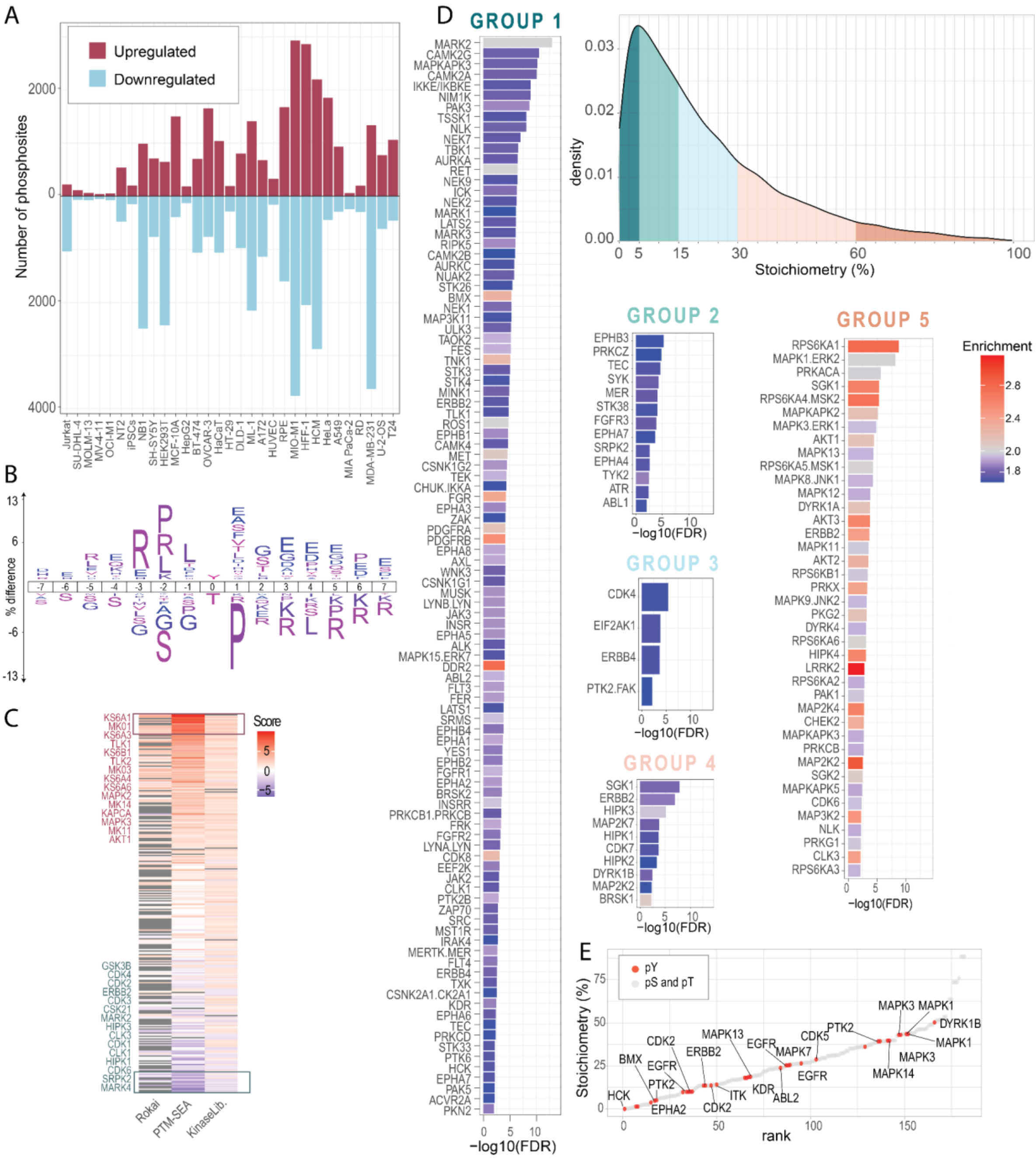

Figure R3: Site stoichiometry analysis recapitulates intensity-based results while providing absolute phosphorylation levels and revealing whether kinases drive high- or low-stoichiometry substrate phosphorylation. A- Up- and down-regulated phosphosite count per cell line upon serum stimulation using site based intensity values. Sites with Benjamini-Hochberg corrected FDR values below 1% after limma statistical testing were considered statistically significantly regulated. B- Motif analysis using IceLogo of the sequences around significantly up-regulated (top) and down-regulated (bottom) sites. C- Summary of kinase activity analysis using regulated site intensities, using Rokai, PTM-SEA with the modified iKiP-DB and the Kinase Library. Kinases scored in 2 out of 3 tools are displayed in the heatmap. D- Overall distribution of calculated site stoichiometries in the stimulated conditions divided into stoichiometry bins. Group 1 spans up to 5%, group 2 ranges from 5 to 15%, group 3 from 15 to 30%, group 4 from 30 to 60% and group 5 above 60%. Sites were annotated with the modified iKiP-DB kinase-substrate relationships and over representation analysis using Fisher's exact test was carried out per group. Displayed results are filtered for 1% FDR, enrichment scores >

1.7 and at least 7 substrates for a kinase were required per group. E- Rank plot of the stoichiometries calculated in the stimulated condition for 182 activation sites. pY are labelled in red.

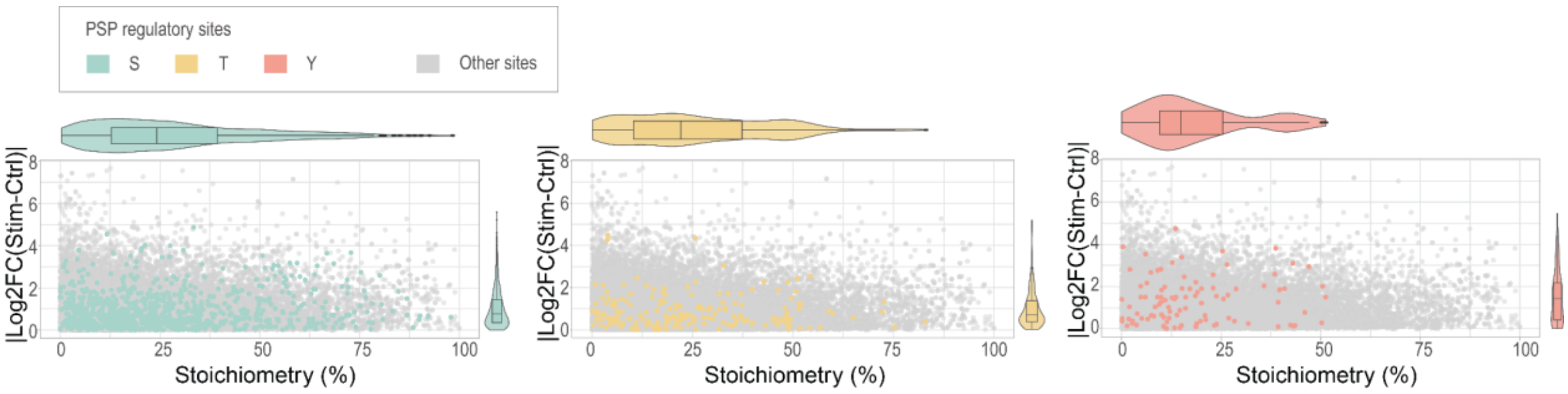

Figure R4: Absolute value of the intensity-based fold change between serum stimulation and control as a function of the stoichiometry value in the stimulated condition. Known regulatory sites from PhosphoSite Plus were colored based on the modified amino acid. Violin plots represent the overall distribution of the stoichiometry (horizontal violins) or the absolute values of the intensity-based fold change (vertical violins) for the regulatory sites.

Figure 5G. The grey color is not defined. What do these mean?

12. The grey color in Fig 5G represents missing values, where a kinase activity score could not be calculated. We have now indicated it in the legend of figure 5.

Figure 6. I don't know how impactful this analysis is, as it largely describes expected results of the tissue or disease origin of cell lines, such as findings that "blood cancer cell lines was characterized by the overexpression of proteins associated with the hematopoietic system and immune cell activation."

13. While we agree that some findings reflect expected biological patterns, we believe that identifying these patterns across a large and diverse panel of cell lines provides valuable validation of the dataset. We have performed this analysis to define functional clusters, and we have now moved the section into an extended data figure.

Figure 7. I think it would be more clear if all references in the figure used "Cscore" instead of Kinase score, as if I am not mistaken, these are the same things?

14. Yes, you are absolutely correct, and we would like to thank you for this observation. We will ensure consistent use of the term "Cscore" throughout the text and figures to avoid confusion. We have now updated the manuscript text, all figures, and corresponding figure legends to exclusively reference the Cscore.

Figure 7D. Is this the definition of "outliers" from the text: "Cell-line specific vulnerabilities were investigated through the identification of kinase-cell line pairs in which a kinase in a given cell line either showed activity substantially higher than its activity distribution across all other cell lines"?

15. Yes, you are correct, this is the way we defined outliers. It basically represents an activity which is outside of the normal distribution of that given kinase across all cell

lines within a 95% confidence interval (ie. Z-score >2). We have added that description in the legend of figure 6 (previously figure 7).

I understand the rationale behind wanting to promote the importance of stoichiometry for prioritizing which phosphorylation sites are most functionally important, but I would encourage the authors to use less rigid language here. For example the discussion describes this as "vital" which is defined as something that is absolutely necessary or essential. I think that this overly simplifies the complexity of proteome regulation. One, it takes a gene centric approach, which we know is inadequate for a proteoform level question, where the presence of a PTM can allow a protein to take on an entirely new cellular function. Secondly, it overlooks the role of additional dimensions to function, such as the local stoichiometry of a PTM, either within a given subcellular compartment, protein complex, etc, where even small changes in stoichiometry (at a global level) may be concentrated to represent substantial changes in a local environment.

16. We agree that the language in the discussion could be more nuanced. We have revised the paragraph describing stoichiometry as vital to acknowledge the limitations of a global approach and the contribution of other regulatory layers. The paragraph has been rephrased into:

"Determining phosphorylation site stoichiometry adds a crucial quantitative dimension to large-scale phosphoproteomics data. This information reveals not just where phosphorylation occurs or if it is regulated in a given condition, but how much of a protein is modified at each site. Stoichiometry data can enable researchers to distinguish between functionally relevant and incidental phosphorylation events, prioritize therapeutic targets, and build more accurate models of cellular signaling networks. While phosphorylation stoichiometry provides a direct, biologically meaningful measure of signaling activity, its functional interpretation should be considered alongside other regulatory layers such as proteoform diversity, subcellular localization, and protein complex context."

This section of the methods does not make sense to me. Why are there two different columns and two different sources described?: "All samples were analysed on the Evosep One system (Evosep Biosystems, Denmark) coupled to an Orbitrap-Astral mass spectrometer (Thermo Fisher Scientific, Germany). Standard gradients were used with commercial Evosep columns (30 SPD (samples per day) with EV1137: 15 cm x 150 µm, 1.5 µm 100 SPD with EV1109: 8 cm x 150 µm, 1.5 µm) connected to a stainless steel emitter (30 µm i.d., Evosep, EV1086), with temperature set to 40°C through an externally controlled butterfly heater (MSWil, Cat#PST-ES-BPH-20). The columns were interfaced to the mass spectrometer using an EASY-Spray source. Whisper zoom gradients were used with Aurora Rapid columns (5 cm x 75 µm, 1.7 µm) interfaced to the mass spectrometer using a Nanospray Flex source, with column temperature maintained at 50°C."

17. Thank you for highlighting this confusion. There are two sections in this method paragraph since we have used different setups based on the input amount and consecutively the gradients used. We have a first setup using a commercial Evosep column and an EASY-Spray source when using the standard high-flow Evosep gradients (ie. 30, 60, 100 SPD). For sensitive (phospho)proteomics analyses, we are using a different setup using the commercial IonOpticks columns interfaced with a nanoflex source for the low-flow Whisper Zoom gradients (ie. 80, 120 SPD). We have clarified the method section to clearly separate both setups.

There appear to be no supplementary tables included with this manuscript. Supplemental tables should be included that contain key datasets, such as the full list of phosphorylation sites, phosphorylated peptides, proteins and their quantitative abundances across cell lines and individual replicates. Likely additional tables of key data from other figures would be helpful as well, such as the kinase activity scores across cell lines.

18. We thank the reviewer for their comment. We have included to the revision the requested supplementary tables detailing key datasets such as the complete list of phosphosites, phosphorylated peptides, proteins, quantitative abundances across cell lines, and kinase activity scores. Additionally we are providing source data for the figures displayed in the manuscript.

    Furthermore, we are providing the code for the Cscore calculation in Github, and we have developed a web interface allowing to visualise our data, including protein and phosphosite abundances (as raw intensities or fold change against the background), the Cscore across cell lines with the addition of a SKEW factor representing the relative contribution of the presence vs the activity in the Cscore, and a protein sequence-based visualisation of the quantified phosphorylation site per cell line.

Please further define what was done for tail-based imputation.

19. Missing value imputation was performed in a two-step approach using the PhosR software package, comprising site- and condition-specific imputation (scImpute) and paired-tail imputation (ptImpute) both taking into account the value of the quantified phosphosites as well as the experimental design.

    The tail-based imputation was carried out using the tImpute function. tImpute carries out tail-based imputation "as implemented in Perseus", where missing values (remaining after the first round of scImpute imputation) are replaced by values from the lower end of the normal distribution of the identified phosphosites, simulating low abundance. In our case, imputation was carried out per cell line such that the numbers were drawn from the intensity distribution per cell line.

For phosphorylation experiments, cross run normalization was enabled in the Spectronaut analysis, but then additional mean centering normalization is performed in the data processing phase? Why does normalization need to be performed twice? Is this due to imputation?

20. The reviewer is absolutely right here, we performed a two-step normalization strategy. The first normalization step was performed for each cell line dataset separately, where the 8 raw files per cell line were searched and normalized using Spectronaut´s cross-run normalization. The second step of normalization was carried out outside of Spectronaut by global normalization across cell lines. It is not due to imputation, however, we have carried out imputation before global normalisation since both imputation steps are dependent on the condition-specific or global intensity distributions.

    As we can see in the **figure R5** below, the need for global normalization does not originate from the imputation.

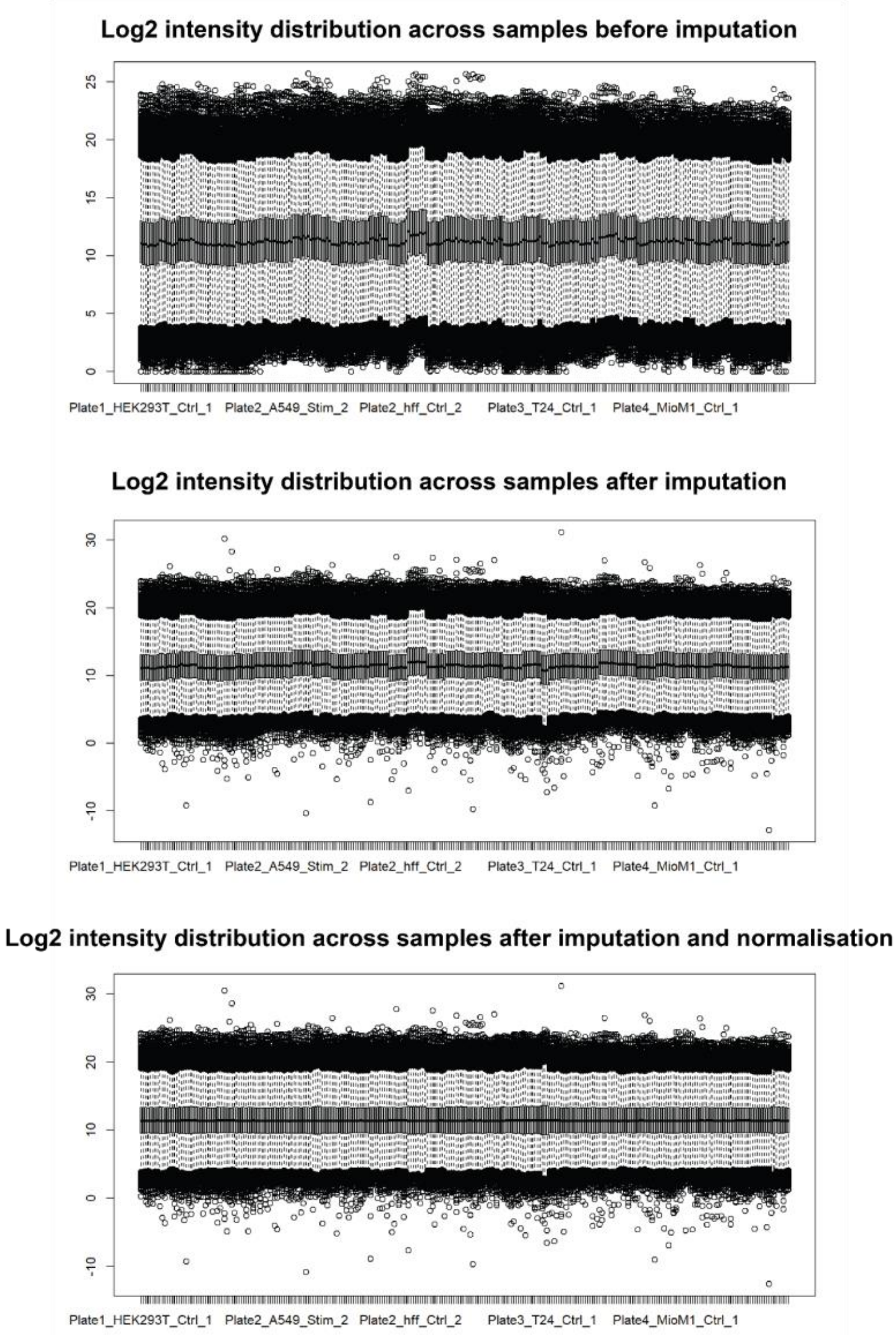

Figure R5: Log2 intensity distribution across raw files before imputation, after imputation and after global normalization.

Each cell line is searched separately, then merged. Does this have an effect on FDR? This is briefly mentioned in the text in relation to phospho tyrosine signaling ("it cannot be excluded that it could be an artifact related to FDR control across searches performed separately and subsequently merged") but this is not expanded on.

In light of the reviewer's concerns regarding FDR control when merging searches using the SNE combine functionality in Spectronaut, we have further looked into our data analysis strategy. According to Spectronaut's guidelines, proper FDR control in a combined dataset requires that all searches be performed against the same spectral library. Originally, we combined directDIA-based searches, which all have their own search-specific spectral library.
To address this, we have reprocessed the entire dataset and downstream analysis to comply with Spectronaut's guidelines by searching all files (by batches of 5 cell lines)

using the generated (phospho)peptide spectral library (built through the search archives of the directDIA-based searches), and finally, we have combined all library-based SNE into a single one using the SNE combine functionality. Overall, this approach allowed to increase the proteome and phosphoproteome coverage per file with an average of 10.2k protein groups and 40.2k class1 phosphosites per cell line, compared to 9.7k protein groups and 30.4k phosphosites in our original approach.

However, during the downstream analysis of the data using the recommended approach, we noticed some inconsistencies driven by a higher completeness of the dataset, with the quantification of proteins and phosphosites in cell lines where their expression is not biologically expected. This is illustrated using the kinases EGFR and ERBB2, only expressed in epithelial cells, and FLT3 only expressed in MOLM-13 and MV-4-11.

We can observe in **figure R6** below, that on the proteome dataset, EGFR and ERBB2 were quantified in some files from suspension cell lines (Jurkat, SU-DHL-4, MOLM-13, MV-4-11 and OCI-M1). Additionally, FLT3 could be quantified in almost all cell lines, including the 8 replicates of human cardiomyocyte (HCM). We could observe that these unexpected quantifications were carried out on a low number of precursors (1-5), while the same protein in a cell line where it was expected was performed on a higher number of precursors (>30). If we were to filter the dataset based on the number of precursors used for quantification, the average protein group count per file would drop to 7.2k.

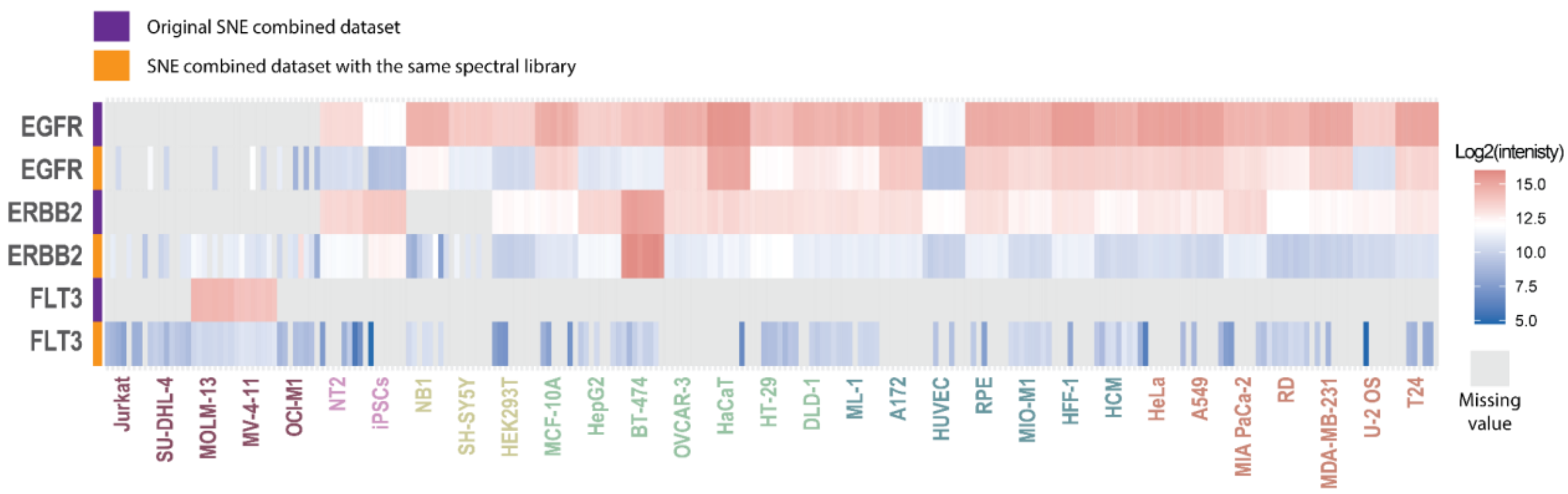

Figure R6: Heatmap of log2-transformed intensities of EGFR, ERBB2, and FLT3 across all samples, comparing the original dataset (combined directDIA searches) and the reprocessed dataset (common spectral library followed by SNE combine). Data are shown prior to filtering and normalization.

A similar pattern was observed at the phosphoproteome level (**Figure R7**). While the reprocessed dataset showed higher site coverage, it also contained phosphosite quantifications for EGFR and ERBB2 in suspension cell lines, where these proteins are not expected to be expressed. In contrast, the original dataset displayed lower completeness but cleaner, biologically consistent absence patterns.

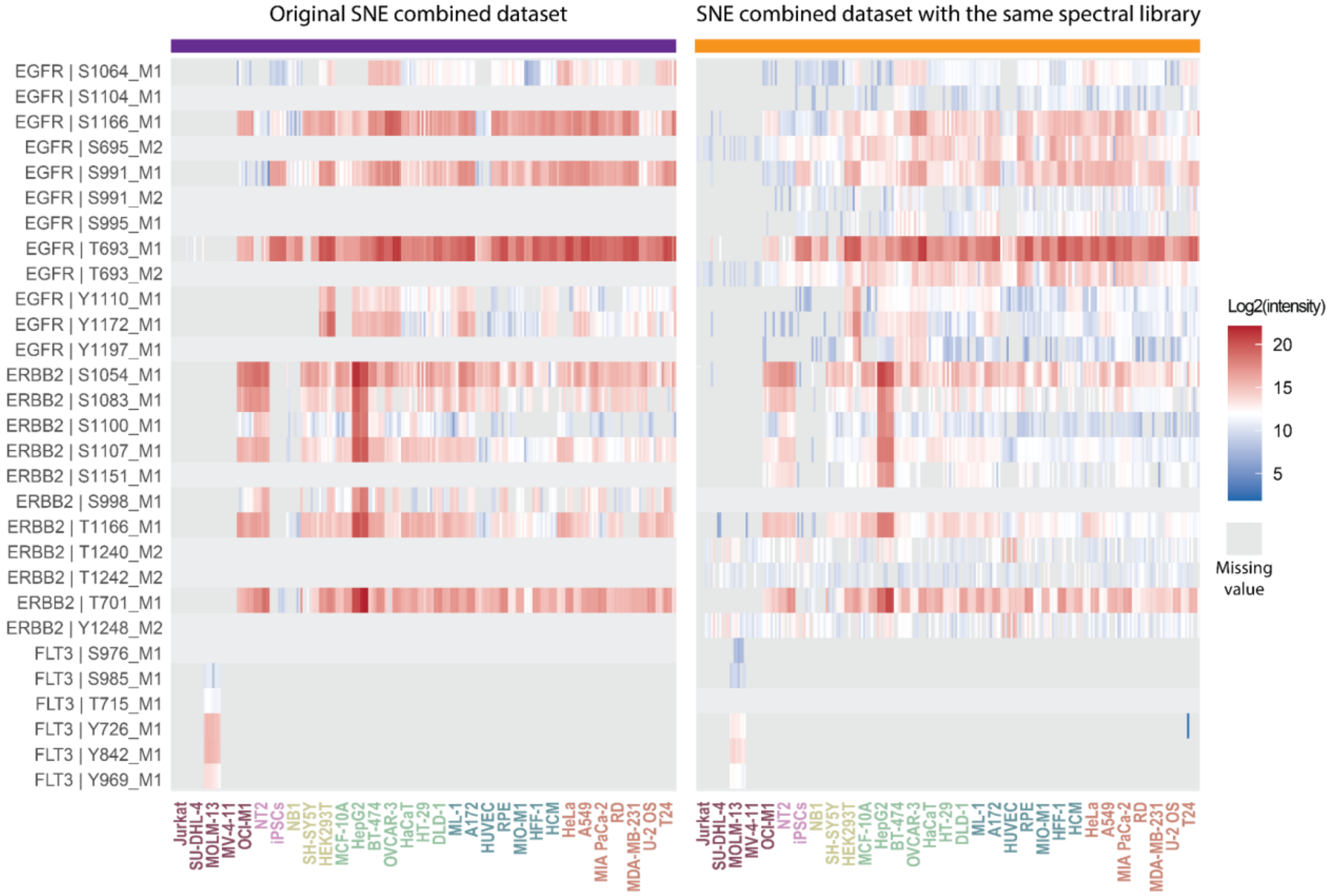

Figure R7: Heatmap of log2-transformed intensities of EGFR, ERBB2, and FLT3 phosphosites across all samples, comparing the original dataset (combined directDIA searches) and the reprocessed dataset (common spectral library followed by SNE combine). Data are shown prior to filtering, normalization, and imputation. Only sites quantified in > 130 samples for EGFR and ERBB2, and > 8 samples for FLT3 are displayed.

Based on our re-analysis, we have decided to use the original dataset generated from combined directDIA searches. Although the reprocessed dataset achieved greater depth, we conclude that this increase is largely driven by the integration of low-confidence signals. To ensure a conservative and biologically reliable analysis, we prioritized the dataset with higher specificity.

Altogether, in this manuscript, spectral libraries were generated through search archives, and FDR control is applied during the search. The downstream analysis of both proteome and phosphoproteome data was only carried out on confidently quantified protein groups and phosphosites, requiring quantification in at least 3 replicates per condition (control or stimulated) per cell line.

Signed,

Danielle Swaney
Associate Professor
UCSF

Reviewer #2 (Remarks to the Author):

Overview:

This manuscript presents an exceptionally deep and technically sophisticated human cell line phosphoproteome atlas, comprising >223,000 class I phosphosites across 33 diverse human cell lines. By leveraging narrow-window DIA on the Orbitrap Astral and constructing a large empirical phosphopeptide spectral library, the authors convincingly demonstrate improvements in phosphosite coverage, site localization confidence, and computational efficiency. The work further integrates proteome and phosphoproteome data to derive a combined kinase activity score (Cscore), enabling cell line–specific signaling inference and correlation with drug sensitivity.

Overall, this is a high-quality, well-executed, and highly valuable community resource that clearly fits the Resource format of Nature Structural & Molecular Biology. The dataset will be broadly useful for signaling biology, (phospho)proteomics-centered method development, drug discovery, and translational research.

21. We appreciate the positive feedback and helpful suggestions for improving the manuscript. You can find below our detailed answers to your comments.

Comments:

1. The empirical phosphopeptide library generated from 33 diverse human cell lines represents a major strength of this work. To further guide future users, it would be helpful for the authors to briefly discuss the expected scope and potential limitations of the library when applied to biological contexts not represented in the panel (e.g., different disease or special tissue samples). A short discussion or summary analysis highlighting pathway, motif, or kinase-family coverage would further enhance transparency and usability.

22. The reviewer is right, we should discuss further the limitations inherent to the use of a spectral library vs. a library-free approach, especially in the case of phosphoproteomics where phosphosites are present in specific cell lines, tissues or context and not only regulated through different expression levels. We have demonstrated that our library had high coverage over most curated signalling pathways (**Figure 2H**). Additionally, we have shown through the use of differentiated stem cells that within an uncontrolled cell population, not included during the library generation, our library showed enough completeness as to outperform or perform on par with library-free approaches (**Figure 3B**). Therefore, we believe that our empirical library could be employed in most contexts. However, we will add a note in the discussion highlighting that most contexts do not represent all contexts, and phosphosites requiring specific activations, or phosphosites on the non-canonical proteome would not be detectable using the library.

    "While our deep empirical spectral phosphopeptide library showed extensive completeness, phosphorylation events are highly tissue- and context-specific.

Therefore, phosphosites unique to tissues not included in our panel, or present on the non-cannonical human proteome, or phosphosites requiring specific activations would not be detectable."

Based on proteome analysis, we could demonstrate that our cell line panel contains most of the canonical human phosphoproteome and covered 501 human kinases spanning across all kinase families without bias (**Figure 1F**). Additionally, our empirical phosphopeptide library covers over 60% of the curated sites from PTMSig DB. We can observe in the data source information of Figure 2H that sites corresponding to all 49 pathways from PTMSig DB could be detected. However, only very little sites could be detected in small pathways containing as little as 6 annotated phosphosites.

2. The combined kinase activity score (Cscore) is a valuable conceptual advance that improves kinase activity inference, particularly for tyrosine kinases. The authors may need to provide detailed information to clarify how to combine different scoring method into Cscore. To further support its general adoption, the authors could add a short clarification regarding the relative contribution of the different score components and comment on robustness across kinases with varying levels of annotation.

23. We would like to thank the reviewer for this comment. We have integrated the scores based on the formula provided in the method section, which is now complemented with the script used for the Cscore calculation being available on GitHub, including documentation on how to use it.
We have slightly changed the Cscore based on Reviewer 3's comment regarding the independence of the components of the score, which had to be validated, and with the addition of a SKEW factor representing the relative contribution of the presence vs the activity of the kinase in the Cscore.
First, we assessed pairwise relationships using Spearman correlation coefficients across all kinases and cell lines (**Figure R8A**) between each component. We observed moderate correlations between kinase protein and kinase phosphorylation expressions, as well as between activity- and motif-based scores, indicating that these components capture partially overlapping but still complementary information. Moreover, correlations between abundance-related components (protein/phosphorylation expressions) and activity-related components (activity/motif) were low, indicating that both sides of the product are independent.
The strongest correlation was observed between the activation loop phosphorylation and the kinase phosphoproteomics expression, with a correlation of 0.73. This suggests partial redundancy between these two components. Consistently, both variables also exhibited the highest correlation with the overall Cscore.

To evaluate the impact of the correlation on the Cscore, we calculated the variance inflation factor (VIF) for all components (**Figure R8B**). The VIF is used to detect multicollinearity, where it quantifies how much the variance of a regression coefficient is inflated due to correlation among predictors (ie. 5 components of the score). A VIF of 1 indicates no multicolinearity, where a precictor is not correlated with any other predictor and therefore does not inflate the standard error. In our case, VIF values

ranged from 1.16 to 2.29, with the highest values observed for kinase phosphorylation (2.29) and activation loop phosphorylation (2.12). These values for phosphorylation or activation loop phosphorylation had the largest impact on the score, consistent with their higher correlation and partial redundancy. These are then likely to be overrepressented in the Cscore.
To compensate for the overrepressentation of the kinase phosphorylation and activation loop scores we have applied weights to all the components based on correlations. The weighting did not impact the correlations between components but reduced the contribution of partially redundant ones.

Comparison of the original and weighted scores showed very high correlation of 0.991719, indicating that the overall ranking is largely preserved (**Figure R8D**). Additionally, the overall score distribution remained largely unchanged after weighting, with score distributions almost overlapping (**Figure R8E**), suggesting that while the weighting introduces measurable adjustments, it does not substantially alter the global structure of the score. We have added this panel as extended data figure.

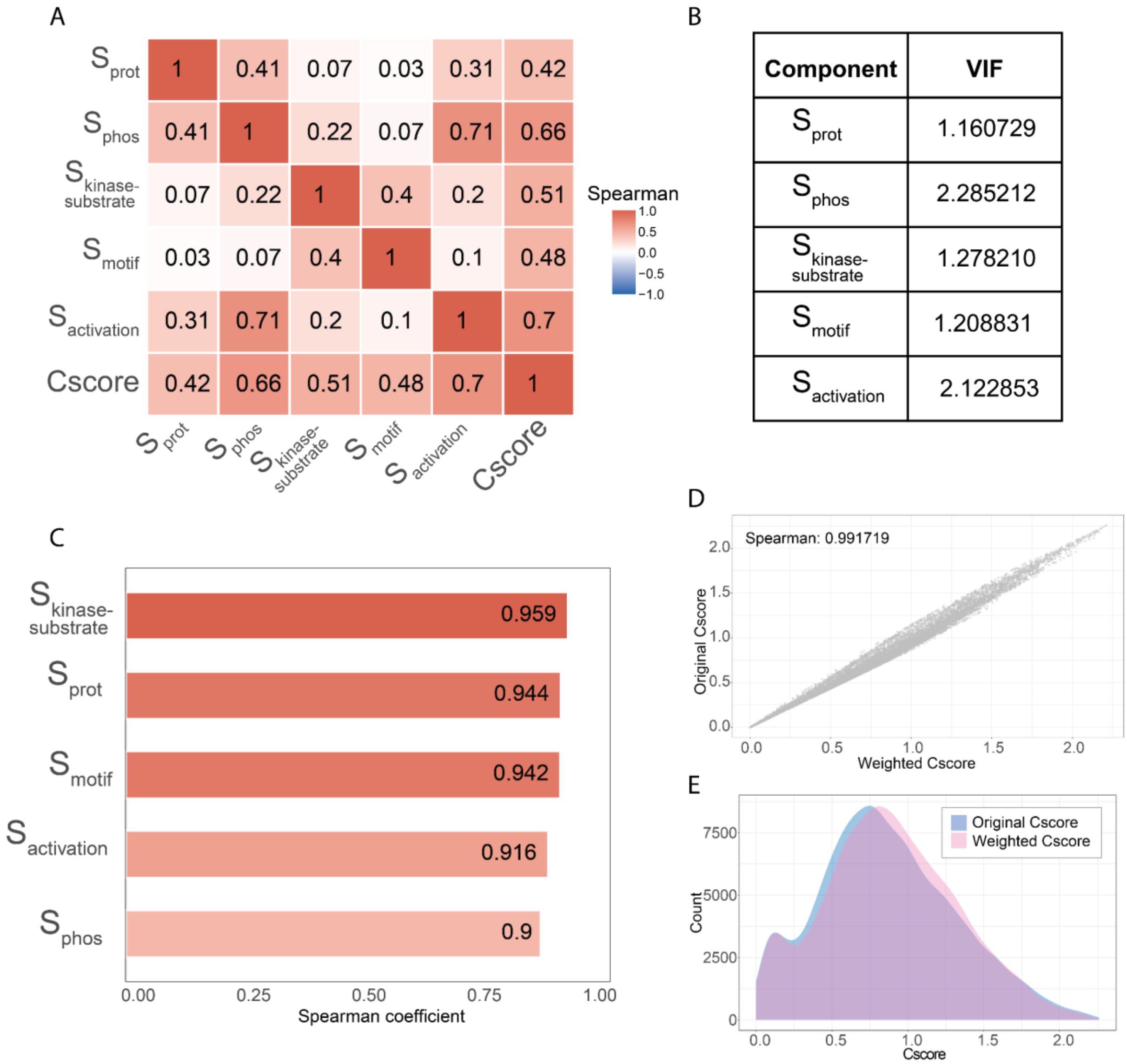

Component	VIF
S_{prot}	1.160729
S_{phos}	2.285212
$S_{kinase\text{-}substrate}$	1.278210
S_{motif}	1.208831
$S_{activation}$	2.122853

Figure R8: Evaluation of the independence of the Cscore components. A - Spearman correlation coefficients between each component of the score across all cell lines and kinases. B - Variance Inflation Ratio (VIF) computed per score component for the detection of multicolinearities. C - Spearman correlation coefficient between the original Cscore and a newly computed score excluding one component at a time. D - Correlation plot between the weighted and unweighted Cscores. E - Cscore distribution for the original and weighted Cscores.

Additionally, we have introduced a SKEW factor similar to the one computed in the INKA score, which captures the relative contributions of both components of the Cscore, namely kinase presence and activity. A SKEW close to 0 indicates a Cscore is mainly driven by the presence of the kinase, while a SKEW close to 1 represents a Cscore mainly driven by activity scores.

$$SKEW = \frac{2}{\pi} arctan\left(\frac{Spresence}{Sactivity}\right)$$

Across our dataset, the SKEW distribution shows that most Cscores are activity-driven, with the presence component acting as a filter rather than the main determinant component (**Figure R9**).

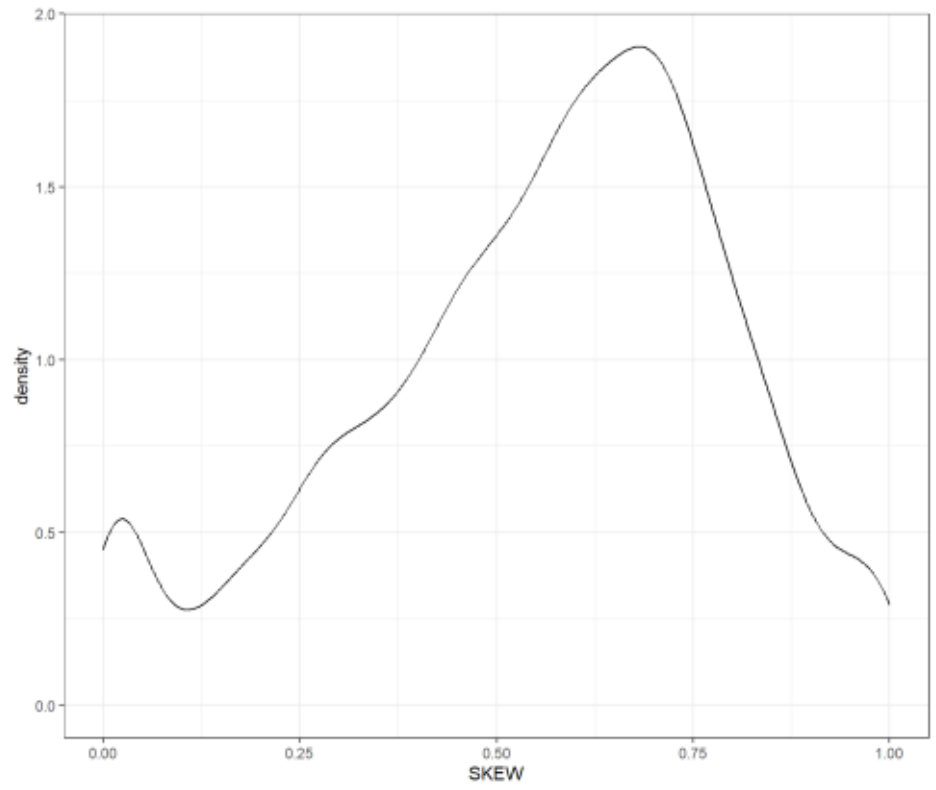

Figure 9: Distribution of the SKEW factor.

Those additions are implemented in the available script, which calculates the weights applied to each component of the Cscore dynamically, on the computed dataset.

The level of annotation of a kinase should have minimal effect on the Cscore. This was our objective while developing it. We are relying very little on Phosphosite Plus annotation. We only rely on databases in the second part of the Cscore (activity) and not for scoring the presence of the kinase. In the activity score, we depend on either experimentally generated or predicted annotations with either in vitro kinase reaction relations (iKiP-DB), motif-recognition sequences through the Kinase Library, or sites in the kinase activation domain based on predictions generated through Phomics. Bias can be present in the relative activity of kinases within a cell line (or sample) where a kinase would have fewer known, or MS-detectable, substrates which can lead to lower statistical power using PTM-SEA or the kinase library. Additionally, kinases for which activation sites could not be predicted would have a relatively lower Cscore. However,

those scores are comparable across samples. We have added this comment in the discussion.

3. The large-scale estimation of phosphorylation site stoichiometry is an impressive addition to the dataset. Given the inherent experimental and computational complexity of stoichiometry measurements, a short clarification on the interpretational boundaries of absolute stoichiometry values (e.g., sensitivity to measurement noise, emphasis on relative trends) would help readers appropriately contextualize these results. Additionally, it is also interesting to comment if there is any specific quantitative threshold to activate a signaling event.

24. We have completed the section in the discussion where we brought up the limitations in stoichiometry calculations to emphasize the sensitivity of stoichiometry measurements to noise and emphasize that relative trends are more informative than absolute values. We also highlighted the lack of clear quantitative references for handling absolute occupancy values such as the lack of clear quantitative threshold for signaling activation. We have rephrased the paragraph as follows using track changes.

"Although stoichiometry offers a valuable complementary perspective on phosphorylation dynamics and has become more feasible with increasing data depth and completeness, the approach remains limited by data sparsity and measurement precision, especially on low abundant phosphoprecursors prone to missing values and quantitative imprecision due to higher noise levels. Each stoichiometry value accumulates the error of four independent intensity measurements (phosphorylated and non-phosphorylated forms in control and stimulated samples), reducing sensitivity for identifying small occupancy changes. Consequently, low-occupancy sites that double upon stimulation may remain within measurement uncertainty and either result in an inaccurate value or the impossibility to compute one, while mid-occupancy sites appear dynamic and lead to an occupancy calculation. Since most sites exhibited low occupancy, even though computed from the high intensity phosphoprecursors, unbiased and precise stoichiometry calculation remains a challenge. Additionally, relative trends using fold changes for instance are overall more informative than absolute values. Reference data to estimate abnormally high or low site occupancies, or clear quantitative occupancy threshold for signal activation are currently lacking. Focusing on stoichiometry at a smaller scale and with emphasis on precision, through targeted approaches for instance [41], would allow to more preciselycould be helpful for unveil crucial information such as site occupancy required for functional activationspecific biological insights."

Reviewer #3 (Remarks to the Author):

Overview:

In the manuscript 'The Human Cell Line Phosphoproteome Atlas: A Deep Empirical Resource Revealing Kinase Activity Landscapes', by Koenig et al. the authors present an exceptionally large dataset coverage (mean of 30,498 phosphosites per run; 223,887 distinct phosphosites identified and 110,291 phosphosites quantified across 33 diverse cell lines), with appropriate and thought-out experimental design. The obtained empirical spectral library is a valuable resource demonstrating clear utility in improving identification depth, computational efficiency, and reducing false positives in directDIA approaches. The authors have also achieved a comprehensive benchmarking of the library across LC gradients and search engines. Furthermore, their novel kinase activity analysis identified key limitations of existing methods and proposed improvements in vulnerability assessment.

25. We thank Reviewer #3 for highlighting the valuable resource that our empirical spectral library will be for the community and for identifying critical areas for improvement. Below are responses to specific comments.

Major comments

• FDR control in merged results

Searches were performed separately per cell line, then merged for library generation. This raises concerns about whether the merged results maintain proper FDR control, particularly for phosphotyrosine sites where sensitivity varies across cell lines. It would be helpful if the authors could discuss in the manuscript regarding the considerations taken to ensure their FDR is indeed properly controlled.

26. We would like to thank the reviewer for his comment. Following Reviewer 1's suggestion, we have tried to discriminate whether the differences observed across cell lines were from biological or technical origin. We have calculated all pairwise overlaps in phosphosite identification across cell line and condition (ie. 408 overlaps representing technical variation) and across cell lines (ie. 561 overlaps representing biological variation). We have plotted the distribution of those overlaps (**Figure R10**) either across or within cell lines and we could observe a higher overlap within cell lines and conditions compared to across cell lines. Thus, technical variability, stochasticity and FDR cannot only be responsible for the site uniqueness observed across cell lines. Moreover, the overlap distribution across cell lines is broader, as expected with the cell lines included in our panel with some being very different and some being rather similar. This analysis as well as the upset plot were carried out on the dataset filtered for valid values (3\4 replicates in control or stimulation) representing consistently quantified phosphosites.

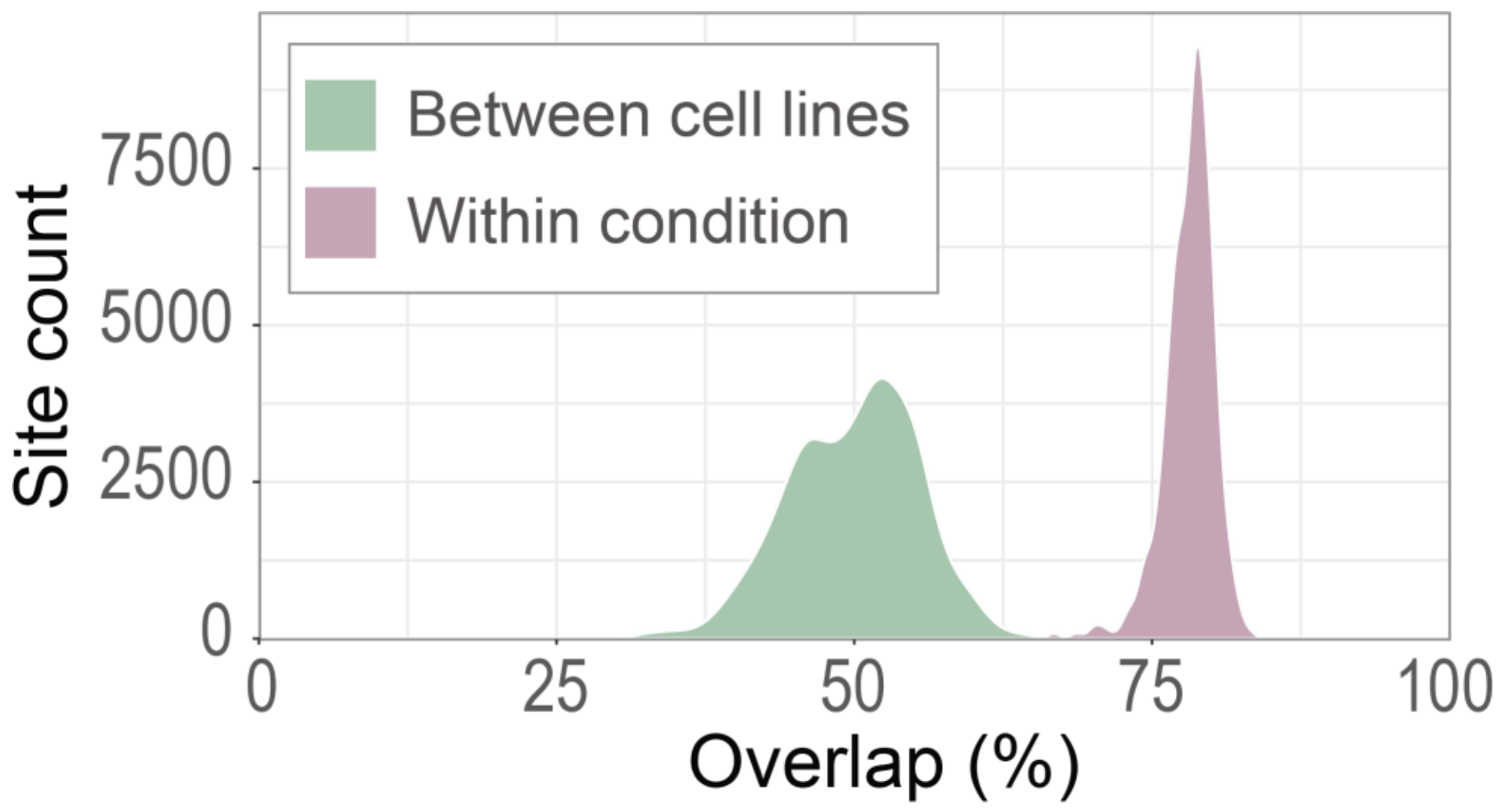

Figure R10: Distribution of pairwise overlaps either between cell lines or within conditions (cell line + treatment).

In light of the reviewer's concerns regarding FDR control when merging searches using the SNE combine functionality in Spectronaut, we have further looked into our data analysis strategy. According to Spectronaut's guidelines, proper FDR control in a combined dataset requires that all searches be performed against the same spectral library. Originally, we combined directDIA-based searches, which all have their own search-specific spectral library.
To address this, we have reprocessed the entire dataset and downstream analysis to comply with Spectronaut's guidelines by searching all files (by batches of 5 cell lines) using the generated (phospho)peptide spectral library (built through the search archives of the directDIA-based searches), and finally, we have combined all library-based SNE into a single one using the SNE combine functionality. Overall, this approach allowed to increase the proteome and phosphoproteome coverage per file with an average of 10.2k protein groups and 40.2k class1 phosphosite per cell line, compared to 9.7k protein groups and 30.4k phosphosites in our original approach.

However, during the downstream analysis of the data using the recommended approach, we noticed some inconsistencies driven by a higher completeness of the dataset, with the quantification of proteins and phosphosites in cell lines where their expression is not biologically expected. This is illustrated using the kinases EGFR and ERBB2, only expressed in epithelial cells, and FLT3 only expressed in MOLM-13 and MV-4-11.

We can observe in **figure R11** below, that on the proteome dataset, EGFR and ERBB2 were quantified in some files from suspension cell lines (Jurkat, SU-DHL-4, MOLM-13, MV-4-11 and OCI-M1). Additionally, FLT3 could be quantified in almost all cell lines, including the 8 replicates of human cardiomyocyte (HCM). We could observe that these unexpected quantifications were carried out on a low number of precursors (1-5), while the same protein in a cell line where it was expected was performed on a higher number of precursors (>30). If we were to filter the dataset based on the number of precursors used for quantification, the average protein group count per file would drop to 7.2k.

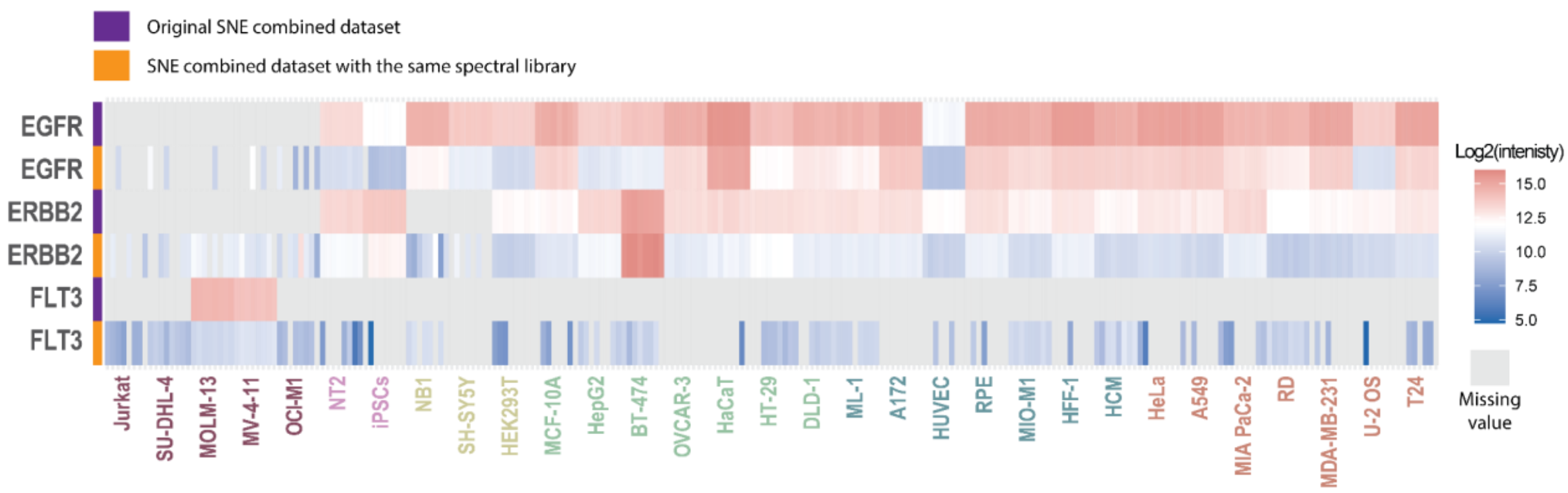

Figure R11: Heatmap of log2-transformed intensities of EGFR, ERBB2, and FLT3 across all samples, comparing the original dataset (combined directDIA searches) and the reprocessed dataset (common spectral library followed by SNE combine). Data are shown prior to filtering and normalization.

A similar pattern was observed at the phosphoproteome level (**Figure R12**). While the reprocessed dataset showed higher site coverage, it also contained phosphosite quantifications for EGFR and ERBB2 in suspension cell lines, where these proteins are not expected to be expressed. In contrast, the original dataset displayed lower completeness but cleaner, biologically consistent absence patterns.

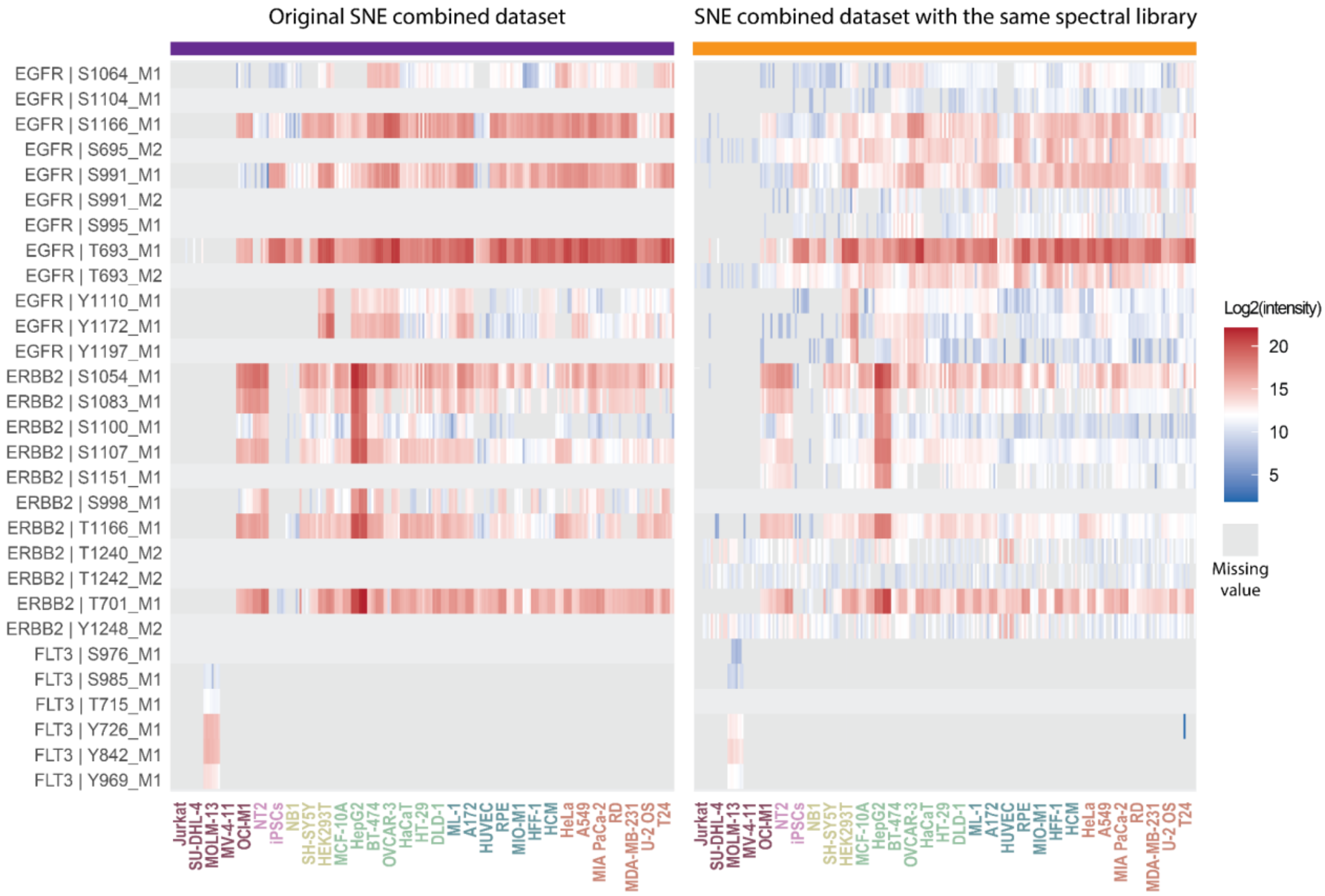

Figure R12: Heatmap of log2-transformed intensities of EGFR, ERBB2, and FLT3 phosphosites across all samples, comparing the original dataset (combined directDIA searches) and the reprocessed dataset (common spectral library followed by SNE combine). Data are shown prior to filtering, normalization, or imputation. Only sites

quantified in > 130 samples for EGFR and ERBB2, and > 8 samples for FLT3 are displayed.

Based on our reanalysis, we have decided to use the original dataset generated from combined directDIA searches. Although the reprocessed dataset achieved greater depth, we conclude that this increase is largely driven by the integration of low-confidence signals. To ensure a conservative and biologically reliable analysis, we prioritized the dataset with higher specificity.

Altogether, in this manuscript, spectral libraries were generated through search archives, and FDR control is applied during the search. The downstream analysis of of both proteome and phosphoproteome data was only carried out on confidently quantified protein groups and phosphosite, requiring quantification in at least 3 replicates per condition (control or stimulated) per cell line.

- Cell line-specific treatments

Pervanadate treatment was applied to only DLD-1 to boost pTyr detection, but the paper does not explain why this was cell-line-specific or whether selective enrichment in one cell line biases the merged library's pTyr composition.

27. We would like to apologise for the lack of clarity in our experimental design. All 33 studied cell lines were serum or factor-stimulated (depending on the possibility of serum stimulation), including DLD-1. Additionally, another batch of DLD-1 was treated with pervanadate, a potent, irreversible inhibitor of protein tyrosine phosphatases (PTPs) widely used to increase cellular tyrosine phosphorylation. Here, DLD-1 was chosen since DLD-1 cells are commonly used to study the role of PTPs in suppressing or activating oncogenic tyrosine kinases like SRC and EGFR. Aberrant phosphorylation, such as decreased PTP activity, contributes to the aggressive, metastatic characteristics of DLD-1 colon cancer cells.

    The PV-treated DLD-1 cells, as well as the serum-stimulated DLD-1 cells, were included for the library generation (to increase the amount of pY), but the PV-treated DLD-1 samples were removed from further quantitative analysis to not bias the relative quantification of pY in other cell lines. We have specified in the method section that different dishes were used for the serum stimulation and PV treatment on DLD-1.

Also the methods section shows some cell lines were treated differently (length of serum starvation, one line not starved, etc) – does this affect the comparisons and inferences made later?

28. Some cell lines were treated differently since starvation protocols are not transferable across all cell lines. Some cell lines included in our panel would not have tolerated starvation, especially for the suspension cells. Additionally, iPSCs were not grown in serum and were therefore starved and stimulated by the removal/addition of factors. Those adaptations were necessary. We believe that those necessary adjustments of protocols would have minimal effect on the downstream analysis since serum

stimulation is a mild stimulation and the panel was designed to include cell lines as diverse as possible.

• Stoichiometry analysis

Stoichiometry was calculated for 1,580 phosphosites on average per cell line, which is only ~5% of phosphosites detected per cell line. For those sites it is valuable information but as the authors highlight in the discussion, since most sites exhibit low occupancy, stoichiometry calculations remain challenging even when there is a big change between conditions.

The analysis of kinases and signalling events in Fig 5 is done on stoichiometry data – so how much does such analysis provide additional insight into signalling upon serum stimulation, compared to just using relative quantification values, for which the data has so much higher coverage? An explicit comparison is not presented but might add helpful guidance to the community.

29. To demonstrate the utility of stoichiometry here, we have performed a similar analysis from the log2-fold change intensities. Per cell line, limma statistical testing was applied to the control and stimulated conditions. Sites with a Benjamini-Hochberg corrected p-values below 1% were considered statistically significantly regulated. The up-regulated versus down-regulated sites were used for the motif analysis and kinase enrichment analysis using Rokai, PTM-SEA, and the Kinase Library was carried out on the regulated sites only. Based on the motif analysis and kinase enrichment analysis we indeed find similar results compared to using stoichiometry values in a dynamic manner **(Figure R13A-C)**. Altogether, combining both analyses brings confidence in the large scale stoichiometry calculation performed.

The logic for doing this analysis on stoichiometry data only might need some clarification. The premise is that stoichiometry is important because, as authors say, "a large stoichiometry for a dynamically regulated site strongly suggests it is functionally important." But kinase inference uses fold-change, not absolute stoichiometry, so both low and high occupancy sites would be ranked equally?

30. The above mentioned sentence is confusing and we meant that a larger stoichiometry for a dynamically regulated site strongly suggests it is functionally active. We have removed this sentence and performed further analysis using stoichiometry values as absolute and not as a trend, with the objective of providing a baseline of site stoichiometry levels within cells.

    Additionally to the previous comparison using a classical site based intensity strategy, we considered the stoichiometry values as absolute metrics and not dynamic ones. We split the stoichiometry range in distinct bins and annotated the sites using kinase-substrate relationships from the modified IKIP-DB. We then performed over representation analysis with Fisher's exact test to identify general trends between kinases leading to substrates with high phosphorylation occupancies and the ones leading to low occupancies (**Figure R13D**). Altogether we could observe that most kinases, including tyrosine kinases and aurora kinases, led to substrates phosphorylated to low occupancies, while MAPKs (ERK, JNK, p38), RSKs and CDKs

are associated with higher occupancies. Focusing on regulatory sites from PhosphoSitePlus, pS sites showed the highest occupancies while pY sites showed the lowest despite displaying the largest fold changes (**Figure 5H**), confirming that a small fraction of tyrosine-phosphorylated peptides is needed to drive crucial downstream signaling. This resource serves as a baseline for understanding site-specific occupancy thresholds activating signalling events, especially with site stoichiometries calculated over 182 activation sites (**Figure R13E**).

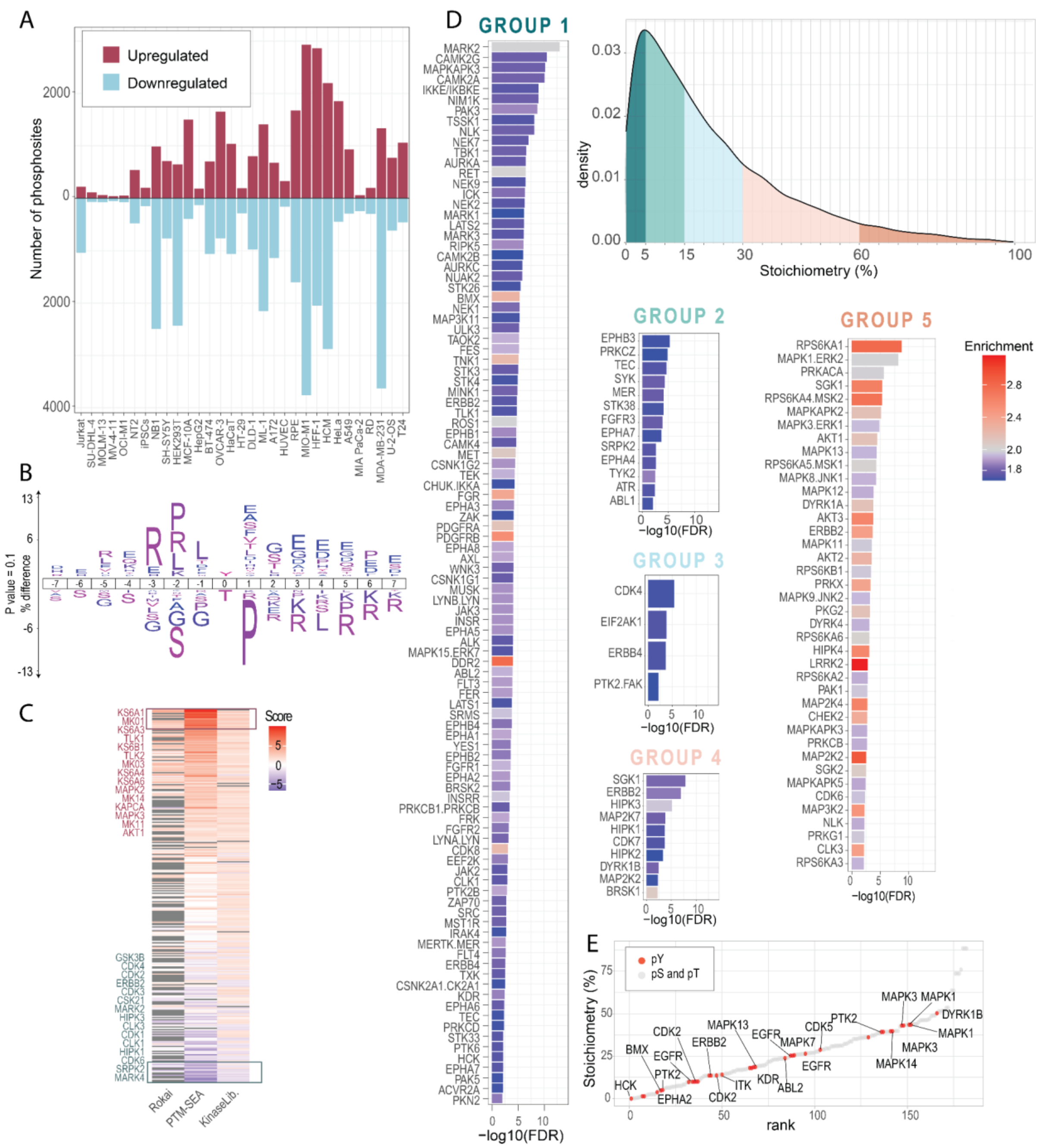

Figure R13: Site stoichiometry analysis recapitulates intensity-based results while providing absolute phosphorylation levels and revealing whether kinases drive high- or low-stoichiometry substrate phosphorylation. A- Up- and down-regulated phosphosite count per cell line upon serum stimulation using site based intensity values. Sites with

Benjamini Hochberg corrected FDR values below 1% after limma statistical testing were considered statistically significantly regulated. B- Motif analysis using IceLogo of the sequences around significantly up-regulated (top) and down-regulated (bottom) sites. C- Kinase activity analysis summary using site intensities of regulated sites, using Rokai, PTM-SEA with the modified iKiP-DB and the Kinase Library. Kinases scored in 2 out of 3 tools are displayed in the heatmap. D- Overall distribution of calculated site stoichiometries in the stimulated conditions divided into stoichiometry bins. Group 1 spans up to 5%, group 2 ranges from 5 to 15%, group 3 from 15 to 30%, group 4 from 30 to 60% and group 5 above 60%. Sites were annotated with the modified iKiP-DB kinase substrate relationships and over representation analysis using Fisher exact test was carried out per group. Displayed results are filtered for 1% FDR, enrichment scores > 1.7 and at least 7 substrates for a kinase were required per group. E- Rank plot of the stoichiometries calculated in the stimulated condition in 182 activation sites. pY are labelled in red.

• Cell line clustering

Consensus clustering was performed on proteome data and kinase activity inference later in the study. Wouldn't additionally direct clustering on phosphosite-level data reveal signaling-specific groups independent of kinase inference assumptions, potentially uncovering kinase-independent signaling events or phosphoproteome complexity not captured at the kinase level?

31. A. Clustering on phosphosite level.

The challenge in performing clustering on phosphosite level is the large presence of missing values in the dataset. As we have in this manuscript, and as Ochoa et al. (PMID: 31819260) has already demonstrated, many phosphorylation sites are tissue-specific, and therefore rightfully present in only a few cell lines. Our dataset compiles over 100,000 confidently localised and quantified phosphorylation sites, with about 30,000 sites per cell line. Consequently, the dataset contains a large amount of missing values, complicating the use of tools for downstream global functional analysis.

We have performed clustering on the phosphosite level using both a Spearman correlation and consensus clustering on i. the unfiltered dataset, ii. the dataset filtered for 70% valid values across cell lines, and iii. the dataset filtered for full completeness. Because consensus clustering does not allow missing values, we applied imputation using a fixed value drawn from the lower end of the intensity distribution.

As we can observe, both clustering strategies allow us to identify the functional clusters with a different level of precision. Overall, Consensus clustering provided better defined clusters, and altogether, the phosphoproteomics data allowed for placing MCF-10A, a non-cancerous breast cell line, as an outgroup to the non-cancerous cell line cluster. Therefore, clustering of the cell lines on the phosphosite level is possible and in agreement with the protein group level clustering.

B. Uncovering signalling dynamics across cell lines

Identifying groups of signalling-specific phosphosites would be much more challenging than focusing on kinase-dependent signalling. First, the visual identification of co-regulated phosphosites across cell lines is not possible (**Figure R14**) due to the sparsity in phosphorylation site detection across cell lines. Computational approaches such as WGCNA (Weighted Gene Co-expression Network Analysis) can allow for the

identification of co-regulated groups of phosphosites. However, this approach is computationally demanding and sensitive to missing values. To the extent of our knowledge, there is no benchmark on the performance of WGCNA algorithm based on the level of missingness or sparsity in the dataset.

We have performed WGCNA on our datasets filtered for 100% completeness and 70% completeness (it was not possible computationally on the unfiltered dataset). We can observe the identification of co-regulated phosphosites across cell lines, likely linked to functionality, where some phosphosite modules in the complete dataset (representing the core signalling) were linked to nuclear protein complexes or mRNA binding. Therefore, the dataset would have the potential to identify kinase independent signalling events, by either fine-tuning and trusting the use of WGCNA-like algorithms on sparse datasets, or focusing on specific signalling events and not performing this type of analysis in a global manner, which we judge outside of the scope of this project.

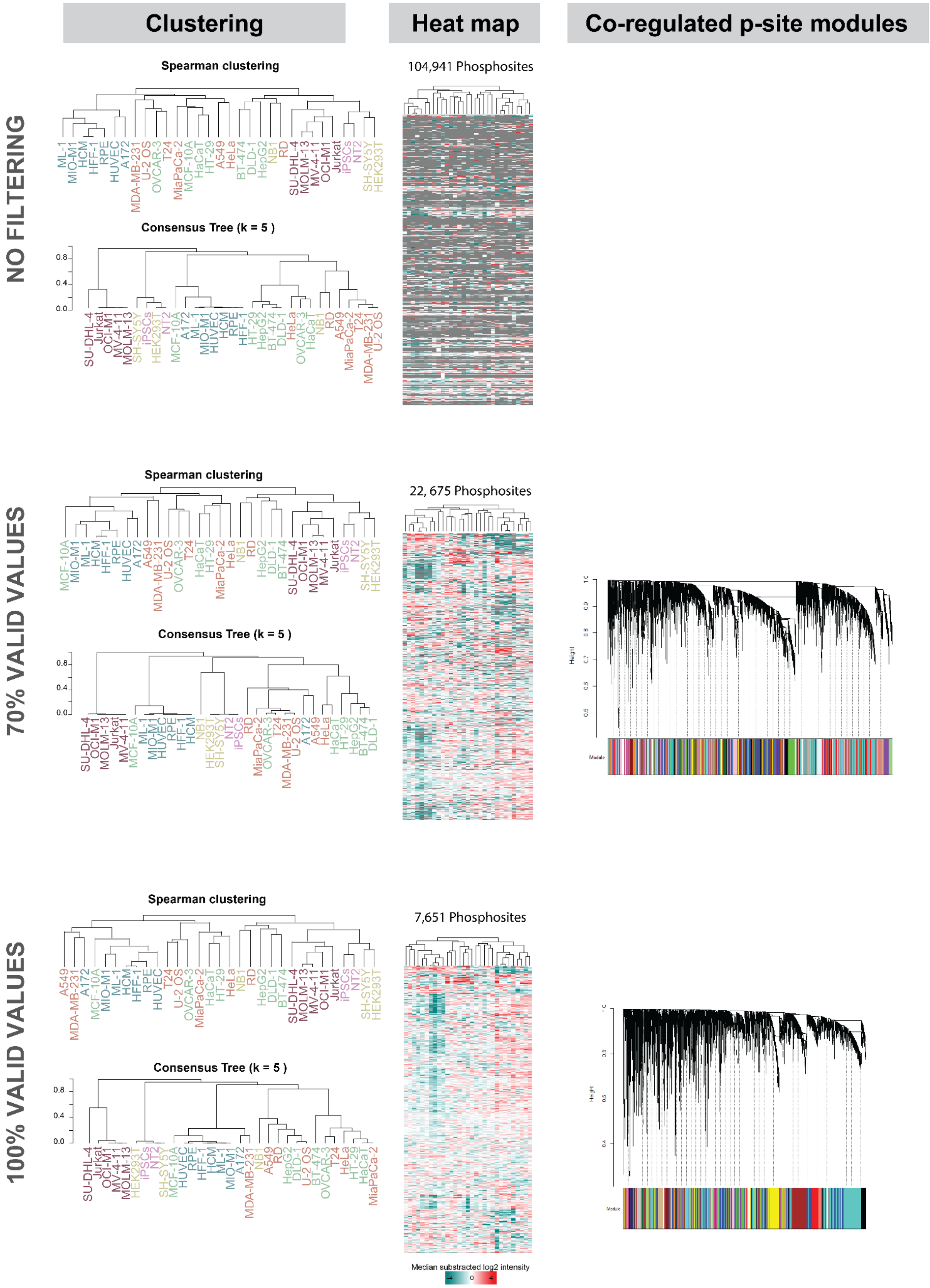

Figure R14: Clustering on the phosphosite level for the identification of signalling-specific dynamics. Spearman correlation-based clustering and consensus clustering were performed on our dataset with different levels of missingness (no filtering on the top, 70% of valid values across cell lines in the middle, and full completeness at the

bottom). Heatmaps showing the level or sparsity in the datasets are shown per dataset using spearman clustering. WGCNA was performed on the different datasets for the identification of co-regulated phosphosites, likely linked to functionalities. The analysis could computationally not be performed on the unfiltered dataset.

• Kinase activity scoring

Kinase activity inference is a valuable part of the study, where comparing existing methods with biological expectations identifies areas where improvements are needed. However, the Cscore may require additional considerations.

The formula used for Cscore appears to treat its components - kinase protein abundance, activation loop phosphorylation, and substrate phosphorylation - as independent evidence streams, which may not be biologically correct. For example, activation loop phosphorylation could gate substrate activity rather than complementing it. The fixed combining strategy could also overweight noisy inputs or underweight informative ones, as the score components have different scales and noise properties.

32. We would like to thank the reviewer for this comment. We indeed have not tested for the independence of the components of the score, which we acknowledge is essential prior to combining scores. To address this, we evaluated the degree of dependency between the score components.
First, we assessed pairwise relationships using Spearman correlation coefficients across all kinases and cell lines (**Figure R15A**) between each component. We observed moderate correlations between kinase protein and kinase phosphorylation expressions, as well as between activity- and motif-based scores, indicating that these components capture partially overlapping but still complementary information. Moreover, correlations between abundance-related components (protein/phosphorylation expressions) and activity-related components (activity/motif) were low, indicating that both sides of the product are independent.
The strongest correlation was observed between the activation loop phosphorylation and the kinase phosphoproteomics expression, with a correlation of 0.73. This suggests partial redundancy between these two components. Consistently, both variables also exhibited the highest correlation with the overall Cscore.

To evaluate the impact of the correlation on the Cscore, we calculated the variance inflation factor (VIF) for all components (**Figure R15B**). The VIF is used to detect multicollinearity, where it quantifies how much the variance of a regression coefficient is inflated due to correlation among predictors (ie. 5 components of the score). A VIF of 1 indicates no multicollinearity, where a predictor is not correlated with any other predictor and therefore does not inflate the standard error. In our case, VIF values ranged from 1.16 to 2.29, with the highest values observed for kinase phosphorylation (2.29) and activation loop phosphorylation (2.12). These values indicate moderate but acceptable multicollinearity (VIF < 5), suggesting that while some dependency exists, however, it is moderate and unlikely to drive the Cscore.

We next performed a sensitivity analysis by recalculating the Cscore while excluding one component at a time (**Figure R15C**). This analysis showed that removal of kinase

phosphorylation or activation loop phosphorylation had the largest impact on the score, consistent with their higher correlation and partial redundancy. These are then likely to be overrepressented in the Cscore.
To compensate for the overrepressentation of the kinase phosphorylation and activation loop scores, we have applied weights to all the components based on correlations. The weighting did not impact the correlations between components but reduced the contribution of partially redundant ones.

Comparison of the original and weighted scores showed very high correlation of 0.991719, indicating that the overall ranking is largely preserved (**Figure R15D**). Additionally, the overall score distribution remained largely unchanged after weighting, with score distributions almost overlapping (**Figure R15E**), suggesting that while the weighting introduces measurable adjustments, it does not substantially alter the global structure of the score. We have added this panel as extended data figure.

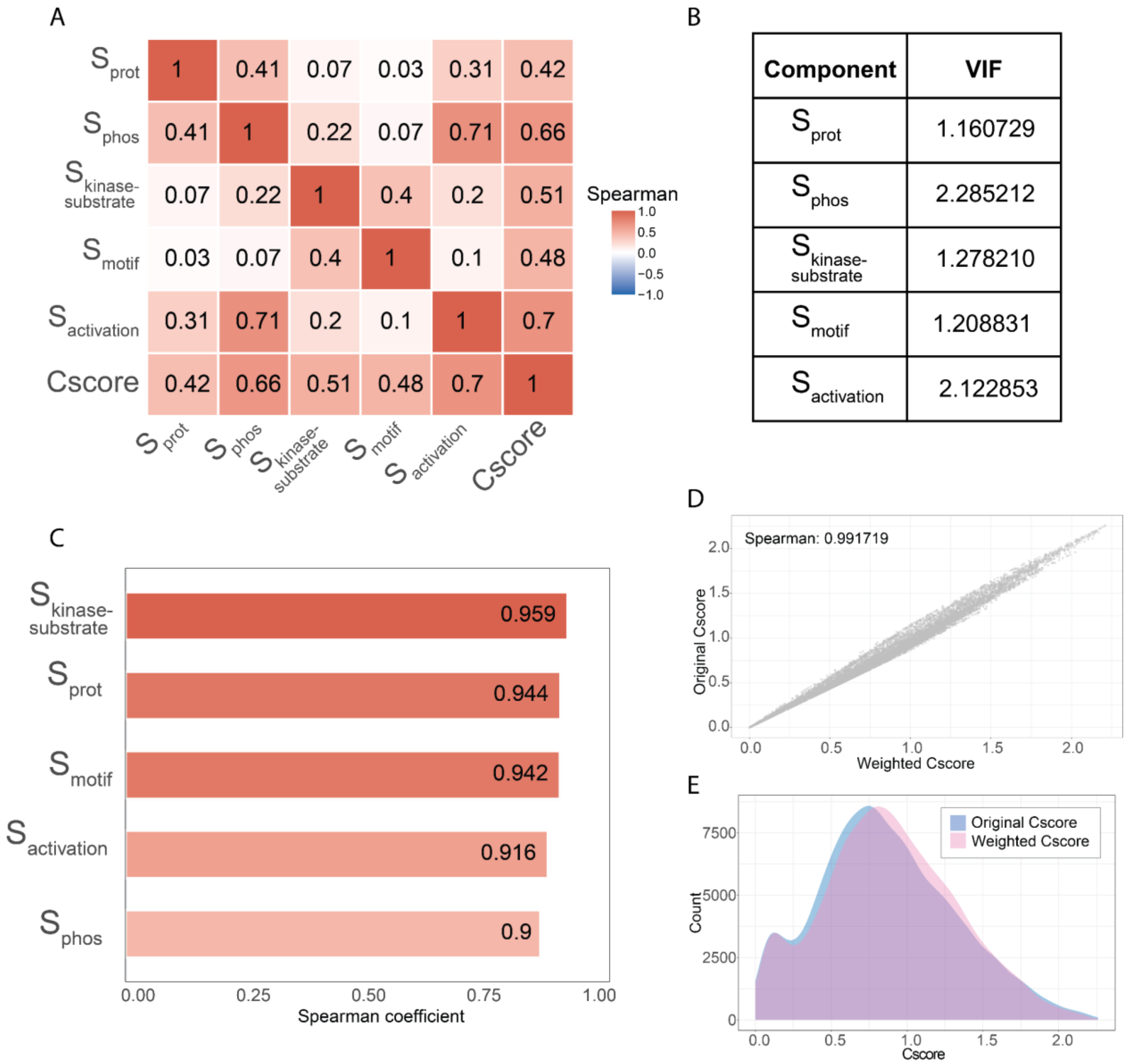

Figure R15: Evaluation of the independence of the Cscore components. A - Spearman correlation coefficients between each component of the score across all cell lines and kinases. B - Variance Inflation Ratio (VIF) computed per score components for the

detection of multicolinearities. C - Spearman correlation coefficient between the original Cscore and a newly computer score excluding one component at a time. D - Correlation plot between the weighted and unweighted Cscores. E - Cscore distribution for the original and weighted Cscores.

To elaborate, would this method distinguish (i) an abundant but autoinhibited kinase with few substrates from (ii) a moderately expressed, activated kinase with moderate substrates? Only the latter represents genuine activity. So a near-zero score could conflate kinase absence (truly inactive), lack of activation loop phosphorylation (could be autoinhibited or unstimulated), and few detected substrates (method limitation). These are biologically distinct scenarios but relying on the score alone makes them mathematically indistinguishable.

33. This is an important point. Being able to distinguish between a highly expressed or highly active kinase would be beneficial. To allow users to distinguish between these cases, we have introduced a SKEW factor, similar to the one computed in the INKA score, which captures the relative contributions of both components of the Cscore, namely presence and activity. A SKEW close to 0 indicates a Cscore is mainly driven by the presence of the kinase, while a SKEW close to 1 represents a Cscore mainly driven by activity scores.

$$SKEW = \frac{2}{\pi} arctan\left(\frac{Spresence}{Sactivity}\right)$$

Of note, the Cscore doesn't only rely on the detection of specific substrates. With the use of motif analysis for inferring kinase activity, kinase activity can be calculated even if detected substrates are not present in a kinase-substrate relationship database.

Across our dataset, the SKEW distribution shows that most Cscores are activity-driven, with the presence component acting as a filter rather than the main determinant component (**Figure R16**).

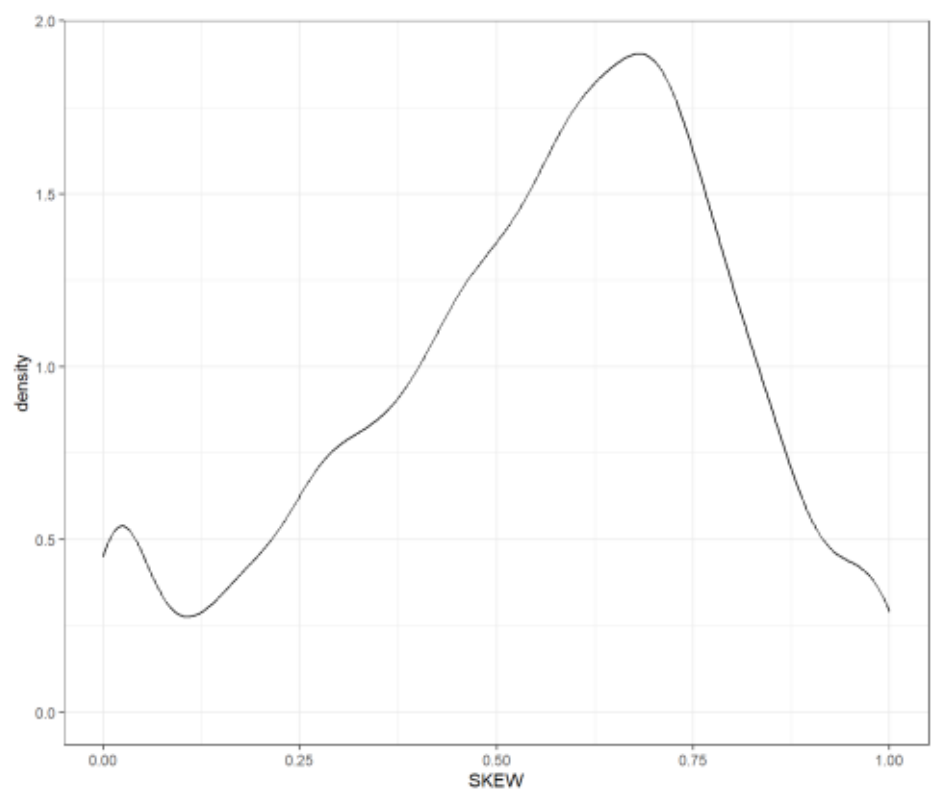

Figure R16: Distribution of the SKEW factor.

Additionally, we are allowing with our web interface the navigation through our dataset integrating the Cscore and SKEW (**Figure R17**). It is possible to visualise the Cscore across cell lines (top left), the Cscore distribution across cell lines as a function of the SKEW (top right) as to identify which cell line shows higher activity and to which extent this activity is driven by kinase expression. On the bottom panel, we added cell line-specific plots to visualise information about the kinase of interest in a specific cell line

context, allowing to visualise the relative activity and SKEW contribution (bottom left), and how the kinase ranks among other kinases in terms of activity within the cell line (bottom right).

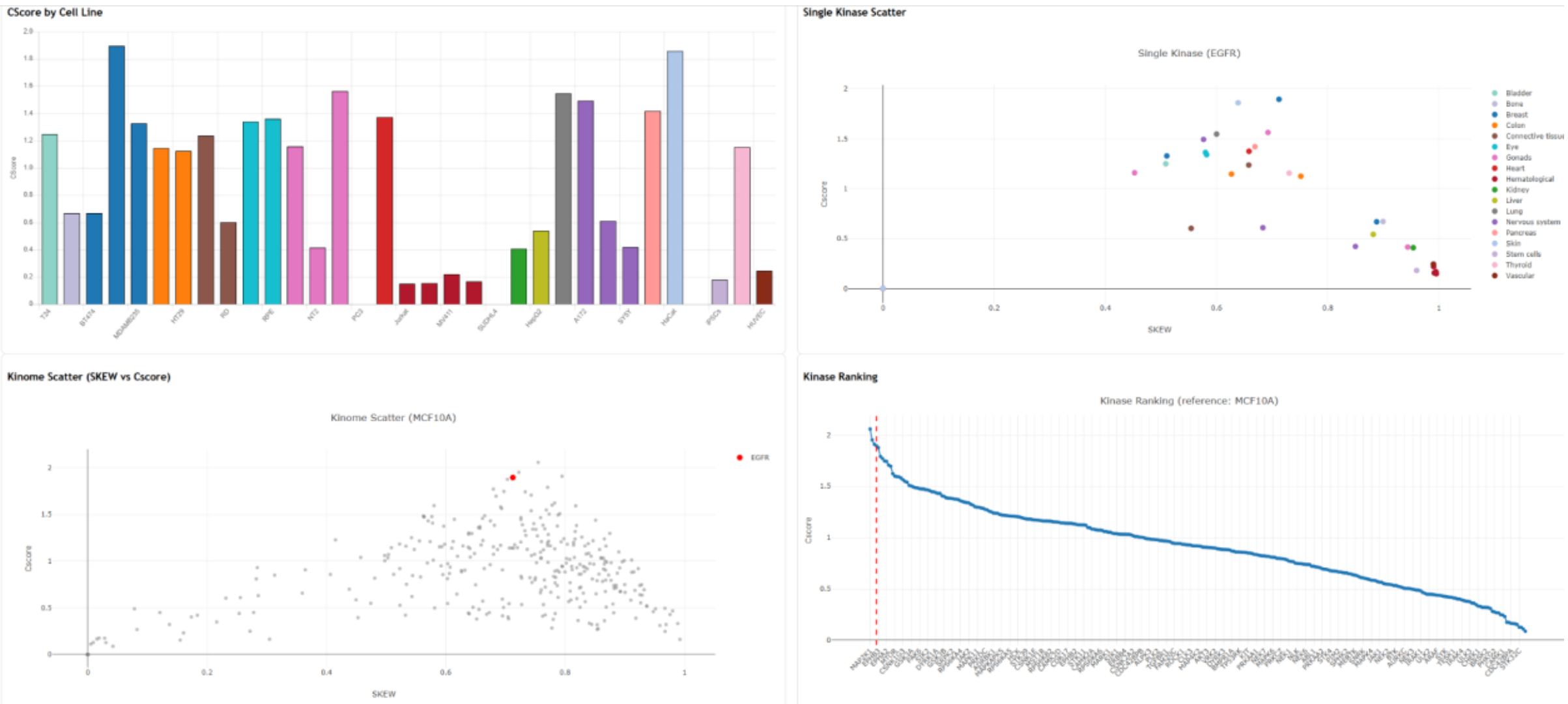

Figure R17: Screenshot of our web interface allowing to visualise our Cscore data.

To validate our ability to distinguish between highly expressed and highly active kinases, we have applied our Cscore and SKEW strategy to a publicly available dataset (PXD038947) (Hybrid-DIA: intelligent data acquisition integrates targeted and discovery proteomics to analyze phospho-signaling in single spheroids - PMID: 37328457) in which HeLa cells were treated with inhibitors of EGFR (Lapatinib), MEK2 (PD0325901), or PI3K (Wormannin), followed by EGF stimulation for 10 and 90 minutes (**Figure R18**). Kinase inhibition represents the situation the reviewer described, where a kinase is expressed but not active. In that case, we expect upon inhibition the Cscore to decrease and to be shifted towards the lower SKEW values, reflecting a shift towards presence-driven kinases.

As proteome data were unavailable for this experiment, we used our own HeLa proteome and phosphoproteome to assess the presence of given kinases by performing Z-score within our cell line for representing the relative abundance of the kinase, instead of computing fold changes against a background. Cscores were computed by combining data acquired with both targeted and DIA methods. Fold changes were computed between EGF-stimulated conditions with and without inhibitor-treatment at each time point.

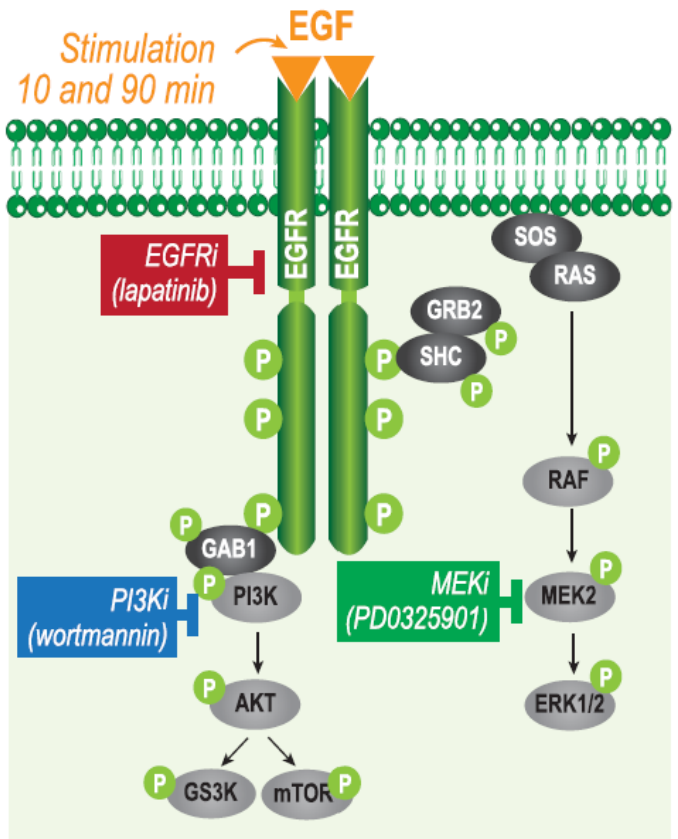

Figure R18: Illustration of the experimental design, taken from PMID: 37328457

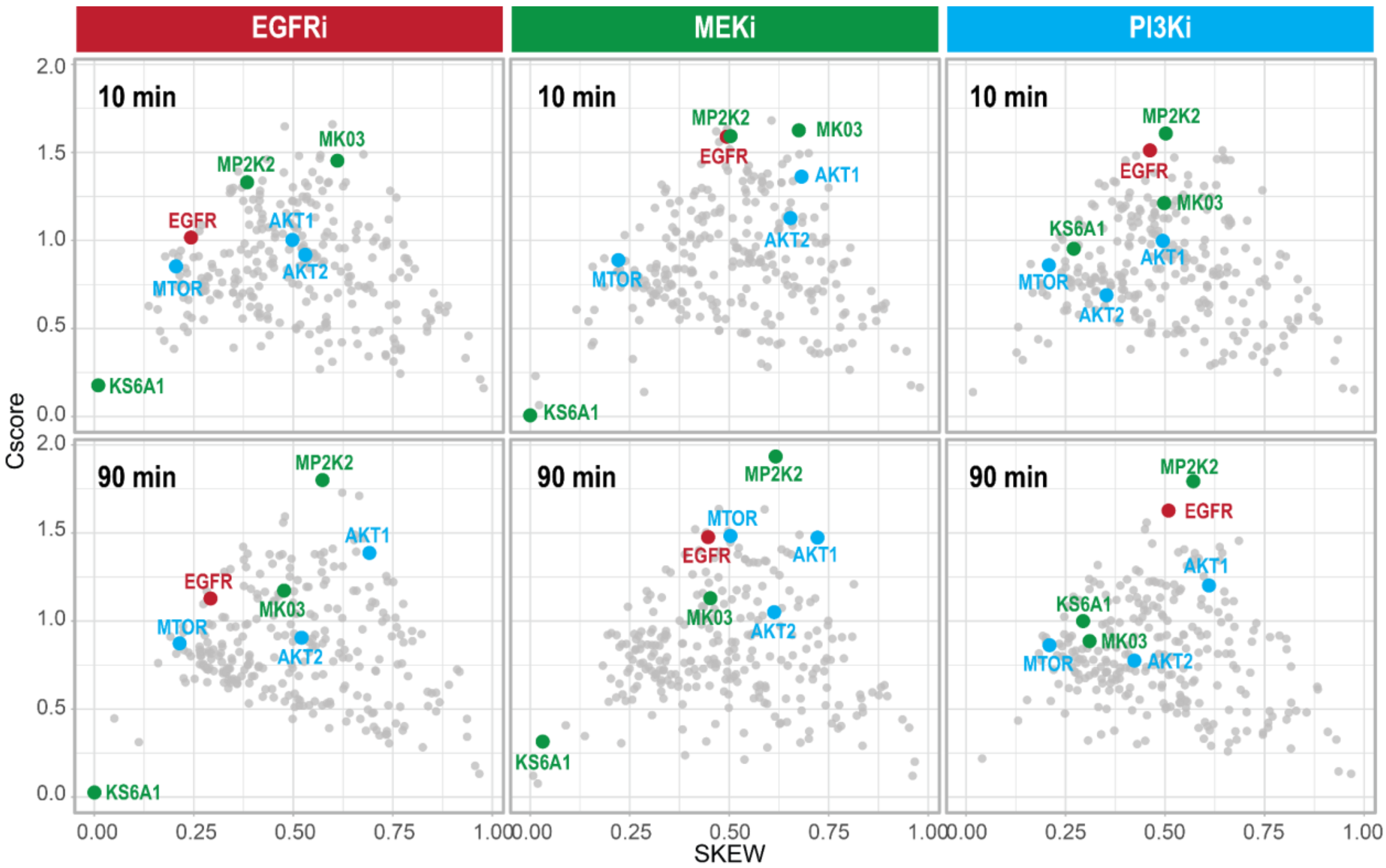

Figure R19: Cscore and SKEW per kinase upon three kinase inhibitors at 10 minutes or 90 minutes after EGF stimulation.

The results were consistent with biological expectations (**Figure R19**). Since EGFR is upstream of both the PI3K and MEK, EGFR inhibition was expected to inhibit signalling through both axes, which is indeed observed.

We can observe EGFR activity to be reduced and shifted towards lower SKEW upon inhibition both at 10 and 90 minutes. PI3K inhibition reduced the activity of its downstream kinases AKT1, AKT2, and MTOR. This pattern is also visible through upstream EGFR inhibition, while their activity remained unaffected with MEK inhibition (except for MTOR at 10 min). Of note, AKT2 remained inhibited longer than AKT1.

MEK inhibition did not produce a detectable Cscore change for MEK2 or ERK1 directly. However, reduced activity of the ERK downstream substrate RPS6KA1 was observed under both MEK and EGFR inhibition.

We followed up on the lack of inhibition observed for MEK2 and ERK1. Threonine and tyrosine activation sites on ERK1 following the TEY motif (T202 and Y204) were quantified from the targeted data. Fold changes per experiment are displayed in **Figure R20**. While the tyrosine phosphorylation was being strongly inhibited upon MEKi, and at 90 minutes with EGFRi (consistent with MEK being downstream of EGFR), the threonine showed higher phosphorylation levels. Since ERK1 activation requires dual phosphorylation, ERK1 is functionally inactive under MEK2 inhibition in our dataset, yet kinase activity inference methods used for the Cscore calculation (PTM-SEA, Kinase Library) reported elevated MEK2 activity, with elevated FDR, likely due to conflicting phosphorylation signals on the substrates **(Figure R21)**. This example highlights the limitations of the mathematical models applied to kinase activity scoring in complex biological contexts, which consequently affect the Cscore.

	PTM-SEA		Kinase Library	
	NES	FDR	NES	FDR
EGFRi - 10min	-1.22	0.467	1.13	0.366
EGFRi - 90min	-2.32	0.128	0.97	0.912
MEKi - 10min	1.60	0.395	1.34	0.089
MEKi - 90min	-5.71	0.009	1.22	0.371
PI3Ki - 10min	-1.77	0.305	0.91	0.841
PI3Ki - 90min	-4.30	0.011	-0.88	0.994

Figure R20: MEK2 (MP2K2) kinase activity scores and FDR using PTM-SEA with the modified iKiP-DB or the Kinase library. NES- Normalised enrichment score. FDR- False discovery rate.

	EGFRi 10min	EGFRi 90min	MEKi 10min	MEKi 90min	PI3Ki 10min	PI3Ki 90min
MK03_T202	0.60	0.93	1.58	-0.56	-0.05	-1.10
MK03_Y204	-0.40	-1.70	-1.60	-1.18	-0.31	-0.66

Figure R21: ERK1 (MK03) log2-fold changes of phosphosites requiring dual phosphorylation for activation.

• Drug sensitivity/vulnerability assessment

The use of kinase activity scoring to assess drug-sensitivity shows qualitative, cell line–specific patterns well. However, to claim identifying therapeutic vulnerabilities rather than just signalling correlates, the study could further benefit from orthogonal functional tests in matched conditions demonstrating that high-scoring kinases are true dependencies (e.g. CRISPR or degrader-based causal links).

34. We would like to thank the reviewer for this comment. Indeed, the kinase-cell line associations should be validated through orthogonal functional tests. For this purpose, we have looked at the CRISPR and SiRNA datasets from DepMap. We observed that

across diverse cell lines, the same kinases consistently showed high dependencies (i.e., CRISPR scores ~ –3 and siRNA scores ~ –1), including essential kinases such as PLK1, WEE1, CDC7, CDK1, and AURKA/B (**Figure R22**). These dependencies exhibited very little variation between cell lines, suggesting that those kinases are more essential for cell survival in general, rather than specific to particular cell lines.

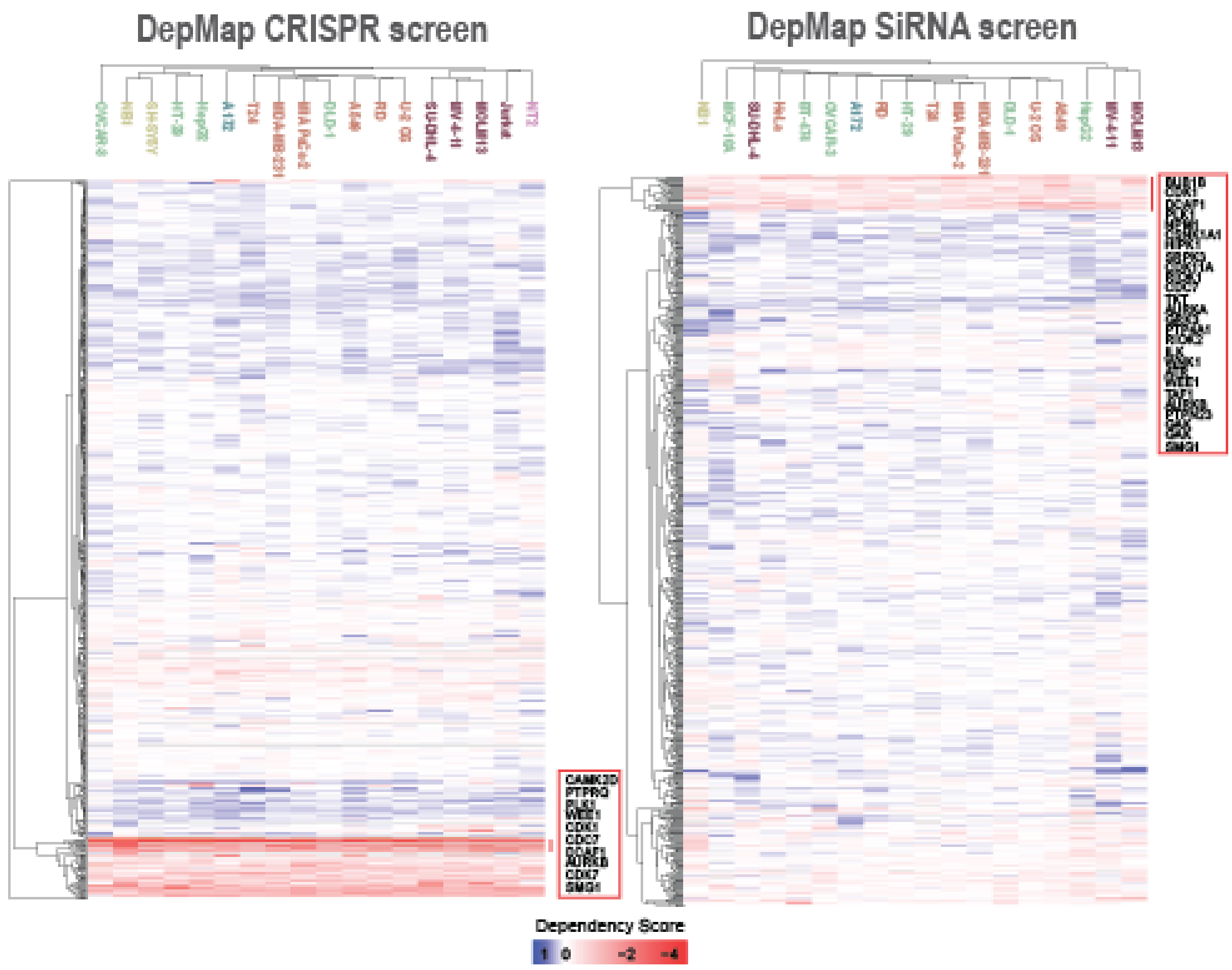

Figure R22: Validation of the functional vulnerabilities. Dependency scores from DepMap (CRISPR screens on the left and SiRNA screens on the right) show that all cell lines are highly dependent on the same kinases for survival.

We further looked into the CRISPR and SiRNA dataset of our cell line panel, regardless of the absolute value of the dependency score, where we investigated the relative dependency of each cell line against the kinases in the cell lines that showed an effect (or data available) by drug inhibition. We performed median subtraction of the dependency scores per kinase across the cell lines and represented the results as heatmap (**Figure R22**).

This analysis validated by both CRISPR knockout and SiRNA MV411 and MOLM13 as dependent on FLT3, NB1 cells as dependent on ALK, and SU-DHL-4 as dependent on SYK. Moreover, BT474's dependency on ERBB2 could also be validated through SiRNA (data not available for CRISPR).

B

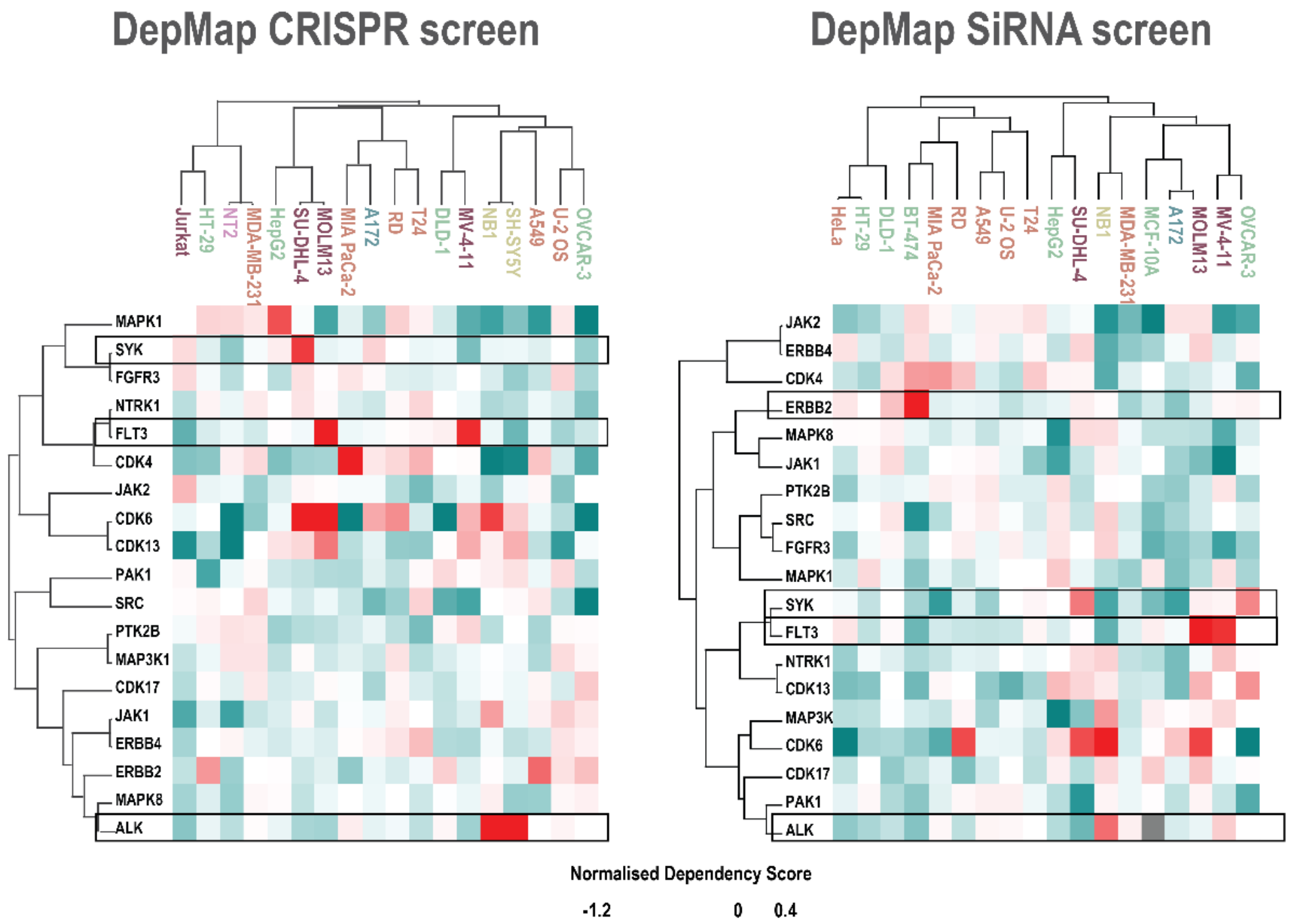

Figure R22: Validation of the functional vulnerabilities. Dependency scores from DepMap (CRISPR screens on the left and SiRNA screens on the right) after median subtraction shows higher dependencies for some of the hypothesized associations.

• Data Accessibility

Importantly, while the raw data is deposited on PRIDE, for the wider community to benefit from this rich resource, authors should provide the analysis results details - incl. abundances, stoichiometry, kinase analysis - in a more accessible manner for the final publication, e.g. as annotated supplementary tables (or optionally as a web resource in addition).

35. Absolutely, it is very important for this resource to be available to the community. Therefore, we have worked on making our results and analysis available through:
    a. Source data available as supplementary tables, regrouping all the data tables and data used for the generation of figures. Results tables will be added as supplementary tables to the manuscript (detailed in the source data overview). Alternatively, we would upload them on Zenodo for facilitating their access.
    b. Cscore calculation script available on GitHub with a user guide on how it was used on our dataset available on GitHub or in the supplementary information. Additionally, scprits used for data processing and stoichiometry calculations are also made available.
    c. Web interface to navigate through the data, including protein and phosphosite quantification, protein sequence visualisation of the phosphorylated amino acids per cell line, and visualisation of the Cscore with SKEW per kinase or per cell line accessible here: https://phosphoproteomicsatlas.github.io/

Minor corrections

Fig2D: D- Upset plot presenting the phosphosite overlap across all 33 human cell lines analyzed and the pervanadate treatment. -> rephrase

36. We have now rephrased it into "Overlap between quantified phosphosites across 33 human cell lines and pervanadate-treated DLD-1 cells, shown as an Upset plot (34 conditions in total)."

Additionally, phosphorylation can only be calculated when a change is observed between both conditions. -> correct for 'stoichiometry can only be calculated…'

37. We have now corrected it as suggested.

Additionally, DLD-1 was treated with pervanadate (PV) for 15 minutes using 0.5 mM and 1mM of PV. -> correct the concentration used.

38. We have used both PV concentrations. We had 4 replicates for the control, 4 replicates treated with 0.5mM and 4 replicates treated with 1mM. We have rephrased the method section and hope it is avoids the confusion:

    "In addition to the serum stimulated DLD-1 cells, 12 other p10 dishes of DLD-1 were treated with pervanadate (PV) for 15 minutes in quadruplicates using either DMSO, 0.5 mM or 1mM of PV."

Point-by-point responses to reviewers NSMB-RS52225A

Reviewer #1 (Remarks to the Author):

I appreciate the efforts by the authors to address the critiques of the first review and find this to be a significantly improved manuscript. Below are a few text edits that I would recommend:

1. While the authors have removed the "as a large stoichiometry for a dynamically regulated site strongly suggests that it is functionally important" at the beginning of section IV as suggested, they have essentially replaced the spirit of that with just longer text in: "because it reveals the actual fraction of a protein population modified at a specific site, offering a true measure of signaling activity. In contrast, relative fold-changes alone can be misleading as the same fold-change may represent either a trivial or a highly significant biological event depending on the baseline occupancy. Moreover, fold-changes are confounded by changes in total protein abundance and do not distinguish between true phosphorylation dynamics and protein expression changes. Site stoichiometry estimation, which integrates both phosphorylated and unphosphorylated forms, provides the necessary context for accurate biological interpretation, functional analysis, and systems-level modeling."

There are so many nuances to the underlying biology of this calculation that can lead to inaccurate biological interpretations of the calculations (e.g. subcellular localization specific impacts on phos state, divergent regulation of individual phos sites on the same peptide, etc.) that my advice would be more diplomatic about this, and simply state something to the effect of "stoichiometry integrates both phosphorylated and unphosphorylated forms and therefore offers a complementary context under which one can perform biological interpretation, functional analysis, and systems-level modeling."

We would like to thank the reviewer for this constructive feedback, but also for her valuable input during the revision process. We have adjusted the beginning of the 4th section accordingly.

2. "This also establishes that regulatory pY sites have very low baseline occupancy, which explains why antibodies are often needed for specific enrichment of tyrosine-phosphorylated peptides for global profiling by LC-MS (Lundby et al. 2019)." I think "establishes" is not the correct word here, this concept is already well-established, I think "supports" would be more appropriate.

We agree, and we should have used a different verb here. We have now replaced “establishes” with “supports”.

3. Regarding this statement: "This highlights the challenge of interpreting drug screens, particularly when drug selectivity is not well described and off-target effects can interfere with the results".

While this is certainly true, I would advise that it should be also balanced with a statement that kinase activity scores are also messy, as there is a lot of overlap in substrates between kinases. That is, both the inhibitors, and our ability to attribute phosphorylation site changes to a given kinase are complicated and imperfect.

We have here focused only on the challenges related to drug selectivity, and should have nuanced it with the challenges inherent with kinase activity calculation. We have now added the following statement:
"This highlights both the challenges of interpreting drug screens, particularly when drug selectivity is not well described and off-target effects can interfere with the results, and inferring accurate kinase activity through phosphosite abundance changes."

Signed,

Danielle Swaney
UCSF

Reviewer #2 (Remarks to the Author):

I appreciate the authors' efforts in addressing all of my comments. The manuscript has been substantially improved, and I recommend publication of this valuable resource article to share with the community.

YuJu Chen

We would like to thank you for the positive feedback and the constructive comments during the revision process, which helped making the manuscript better.

Reviewer #3 (Remarks to the Author):

The authors have done a thorough job at addressing my concerns and I am satisfied with their responses.

My final comment concerns the FDR analysis of the merged search results for this phosphoproteome atlas. FDR control is inherently challenging and the authors have clearly made a thorough effort to manage this by using indirect evaluation methods and maintaining a conservative approach. While determining the true FDR remains an open challenge that falls outside the scope of this study, it represents a broader issue that the community must eventually address. I would appreciate it if the authors could acknowledge this point in their discussion.

We would like to thank you for your valuable input during the revision process, and the help received for improving the manuscript. We have indeed faced tremendous challenges with evaluating the FDR and identifying an optimal strategy allowing for both depth and preserving the

biology of the samples. It is, as mentioned, an open community wide challenge, and we fully agree that it should be mentioned in the discussion.
We have added the following statement:
"Additionally, accurate FDR estimation in phosphoproteomics remains challenging, in part because the full extent of the human phosphoproteome remains unknown, and comprehensive ground-truth datasets for benchmarking search tools are lacking. This limitation is becoming increasingly important as the field moves toward larger-scale studies, with the use of merged datasets, making estimation of a global FDR both more difficult and more critical."

Reviewer #3 (Remarks on code availability):

The code on the github site is well annotated and structured according to type of analysis.

Thank you.
